# Highly Active Small Aminated Quinolinequinones against Drug-Resistant *Staphylococcus aureus* and *Candida albicans*

**DOI:** 10.3390/molecules27092923

**Published:** 2022-05-03

**Authors:** Hatice Yıldırım, Nilüfer Bayrak, Mahmut Yıldız, Fatıma Nur Yılmaz, Emel Mataracı-Kara, Deepak Shilkar, Venkatesan Jayaprakash, Amaç Fatih TuYuN

**Affiliations:** 1 Department of Chemistry, Engineering Faculty, Istanbul University-Cerrahpasa, Avcilar, Istanbul 34320, Turkey; hyildirim@iuc.edu.tr (H.Y.); nbayrak@istanbul.edu.tr (N.B.); 2 Department of Chemistry, Gebze Technical University, Gebze, Kocaeli 41400, Turkey; yildizm@gtu.edu.tr; 3 Department of Pharmaceutical Microbiology, Pharmacy Faculty, Istanbul University, Beyazit, Istanbul 34116, Turkey; fny.951@hotmail.com (F.N.Y.); emataraci@istanbul.edu.tr (E.M.-K.); 4 Department of Pharmaceutical Sciences & Technology, Birla Institute of Technology, Mesra, Ranchi 835 215, Jharkhand, India; deepakshilkar@live.com (D.S.); venkatesanj@bitmesra.ac.in (V.J.); 5 Department of Chemistry, Faculty of Science, Istanbul University, Fatih, Istanbul 34116, Turkey

**Keywords:** antibacterial activity, antibiofilm activity, antifungal activity, bactericidal effect, kinetic study, quinolinequinones

## Abstract

Two subseries of aminated quinolinequinones (AQQs, **AQQ1**–**16**) containing electron-withdrawing group (EWG) or electron-donating group (EDG) in aryl amine moiety were successfully synthesized. Antimicrobial activity assessment indicates that some of the AQQs (**AQQ8**–**10** and **AQQ12**–**14**) with an EDG in aryl amine exhibited strong antibacterial activity against Gram-positive bacterial strains, including Staphylococcus aureus (ATCC^®^ 29213) and Enterococcus faecalis (ATCC^®^ 29212). In contrast, **AQQ4** with an EWG in aryl amine displayed excellent antifungal activity against fungi Candida albicans (ATCC^®^ 10231) with a MIC value of 1.22 μg/mL. To explore the mode of action, the selected AQQs (**AQQ4** and **AQQ9**) were further evaluated in vitro to determine their antimicrobial activity against each of 20 clinically obtained resistant strains of Gram-positive bacteria by performing antibiofilm activity assay and time-kill curve assay. In addition, in silico studies were carried out to determine the possible mechanism of action observed in vitro. The data obtained from these experiments suggests that these molecules could be used to target pathogens in different modes of growth, such as planktonic and biofilm.

## 1. Introduction

Antibiotics, dubbed as miracle drugs, play an important role in combating infections and also determine the success of common surgical and organ transplantation procedures [1]. The acronymously named “ESKAPE” pathogens (***E****nterococcus faecium*, ***S****taphylococcus aureus*, ***K****lebsiella pneumoniae*, ***A****cinetobacter baumannii*, ***P****seudomonas aeruginosa*, and ***E****nterobacter species*) are a group of bacteria that have grown resistant to available antimicrobials and are responsible for life-threatening infections [2]. Among ESKAPE pathogens, resistant *S. aureus* remains the most notorious pathogen and is responsible for severe infections [3]. Fungal pathogens such as *Candida albicans*, *Aspergillus fumigatus*, and *Cryptococcus neoformans* are also known to cause severe fungal infections [4]. For example, one of the most common causes of infections in hospitalized immunocompromised patients is *C. albicans* [5]. Epidemiologically, the over 400,000 cases per year of systemic candidiasis have been associated with *C. albicans* at a global level [6]. Therefore, developing antibacterial and/or antifungal drugs with good inhibitory potential against these resistant pathogens remains a key demand.

**Figure 1:**
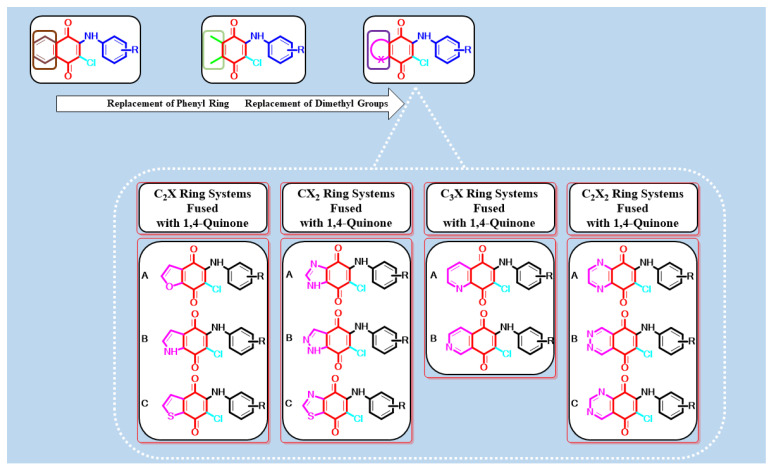
Design strategy for antimicrobial activity based on our previous studies and literature.

The 1,4-quinone core is an important structural motif in a wide range of natural products [7,8,9], pharmaceuticals [10,11,12,13,14], and bioactive molecules [15,16,17]. Over the last 20 years or so, the quinone chemistry, especially in the synthesis of novel compounds showing versatile biological properties such as antibacterial [18], anticancer [19], antichagasic [20], antifungal [21], anti-HIV [22], antimalarial [23,24], anti-mycobacterial [25], antitubercular [26], antiureolytic [27], and antiviral [28] properties, has enabled the design of efficient and general reactions, mostly for the formation of heterocyclic quinones. Because of the significance of this core, considerable efforts have been made to design, synthesize, and explore novel 1,4-quinones fused with C2X [29,30,31,32], CX2 [33,34,35,36,37], C3X [25,38], and C2X2 [39,40,41] ring systems as bicyclic heterocyclic compounds containing one or more heteroatoms such as sulfur, oxygen, and nitrogen shown in Figure 1, to explore their biological profiles. A literature survey on the rational design of antimicrobial agents highlights the importance of the bicyclic heterocyclic quinones [25,42]. From earlier studies, the presence and position of the heteroatom such as nitrogen, sulfur, or oxygen in the ring system have been found to be very important for the activity [43,44,45]. Among a considerable number of those molecules in the literature, such as cribostatin I [46], streptonigrin [47,48], and lavendamycin [49], we have particularly focused on the most important 1,4-quinone fused with C3X ring system where X is a nitrogen atom, named quinolinequinones (QQs). Since their discovery, QQs have gained widespread attention, becoming the most widely used category of antibacterial, anticancer, and/or antifungal agents in drug design [25,42,50,51].

**Figure 2:**
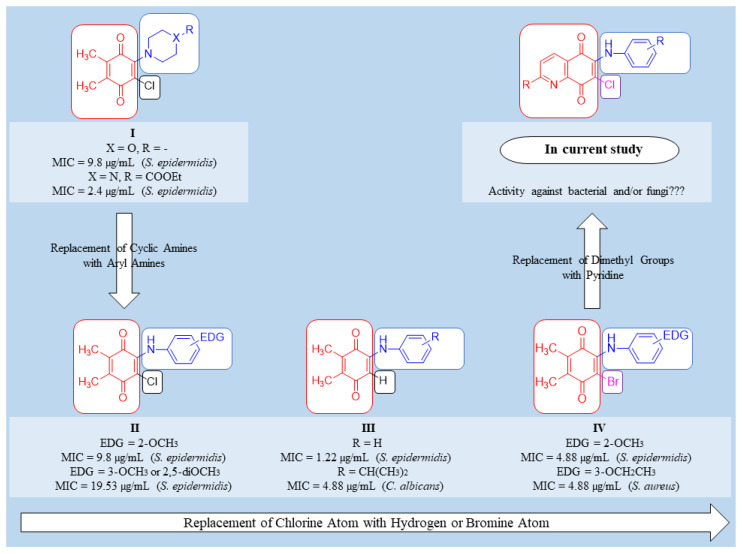
Important aminoquinones employing different groups within 1,4-quinone moiety as a side group possesses interesting biological activities.

When searching for new molecules, the exchange of one bioisostere for another is one of the most effective strategies to improve the desired biological and/or physical properties without making significant changes in chemical structure [52,53]. Among versatile small-molecule antimicrobials developed in our laboratory, the 1,4-quinone molecules fused with phenyl moiety, named 1,4-naphthoquinones, showed significant properties indicative of antimicrobial activity [54,55,56]. Accordingly, we replaced the phenyl moiety of the naphthoquinone with the two methyl groups based on the plant product Plastoquinones (PQs). Furthermore, diverse antimicrobial potencies displayed by dimethylbenzoquinones, named nonhalogenated and halogenated (chlorinated or brominated) PQ analogs, have also been described. Recent results documented by our group showed that dimethylbenzoquinones such as compound **I** (Figure 2) could be a good starting point to identify new potential molecular hits to develop more effective antimicrobial agents. In our previous studies, two important chlorinated PQ analogs of **I** were active against *S. epidermidis* [57]. While the chlorinated PQ analogs of **II** containing electron-withdrawing group (EWG) did not display any significant antimicrobial potency, the chlorinated PQ analogs of **II** containing electron-donating group (EDG) showed excellent antimicrobial activity against *S. epidermidis* [58]. To accelerate the discovery process, replacing the chlorine atom with the hydrogen atom in **III** decreased the activity, especially against Gram-positive strains [59]. The brominated PQ analogs have been obtained by replacing the chlorine atom with a bromine atom. The brominated PQ analogs (**IV**) showed a significant biological potency against bacteria and fungi [60,61]. In our recent studies on this type of 1,4-quinones, we structurally modified the main core by replacing the two methyl groups of the quinone with pyridine moiety to generate QQs. Thus, the presence of dimethyl groups within the 1,4-quinone core instead of phenyl moiety or pyridine moiety within the 1,4-quinone core instead of dimethyl groups seems to be associated with an increase in antimicrobial potency [62]. Our previous structure–activity relationship (SAR) studies and literature have clearly shown three important points: (1) Albeit the quinone molecules with dimethyl groups is important for the biological potency, the azaquinone molecules have displayed relatively higher activity, particularly against most cancer cell lines, bacteria, and fungi [55,58]. (2) Incorporation of aryl amines containing EDG or EWG group(s) into the azaquinone moiety would change the physicochemical properties and hit to a new pharmacophore with a different biological profile [62]. (3) The presence of halogen or hydrogen atoms within the quinone moiety was a considerably important factor in understanding the effects on biological profiles [59,62,63]. Based on the literature investigation and our long-term findings on the synthesis and biological activity studies of 1,4-quinones, we needed novel molecules with versatile substituent(s) within aryl amines. Accordingly, the 1,4-quinone molecules fused with pyridine, named quinolinequinones (QQs), were designed and synthesized to evaluate their antibacterial and antifungal activities in the present study. The corresponding aryl amines containing EWG or EDG were inserted into the quinone skeleton. We also have analyzed the SAR study for further studies. Considering all the available information, the most important AQQs were selected for their antibiofilm activity, potential antimicrobial activity against each of 20 clinically obtained strains of Gram-positive bacteria, and bactericidal time-kill kinetic study.

DmsD (PDB: 3CW0) is a redox enzyme maturation protein implicated in oxidoreductase maturation. It interacts with the twin-arginine leader sequence of DmsA, the catalytic subunit of DMSO reductase. Studies have reported that DmsD may help escort its substrate through a cascade of chaperone-assisted protein-folding maturation events [64]. Thioredoxin reductase 1 (PDB: 3EAN) is critical for redox regulation of protein function and is crucial in the survival of various bacterial species by helping them combat oxidative stress. Studies have shown its importance in the survival of bacteria stress *Staphylococcus aureus*, and others have also targeted the thioredoxin system [65]. Polynucleotide phosphorylase (PNPase) (PDB: 5XEX) is an exoribonuclease responsible for mRNA turnover and rRNA precursor quality control in several bacteria, including *S. aureus*. Considering the crucial role played by these proteins in the survival of *S. aureus,* we selected these proteins to perform molecular docking studies [66].

## 2. Results and Discussion

### 2.1. Chemistry

**Scheme 1:**
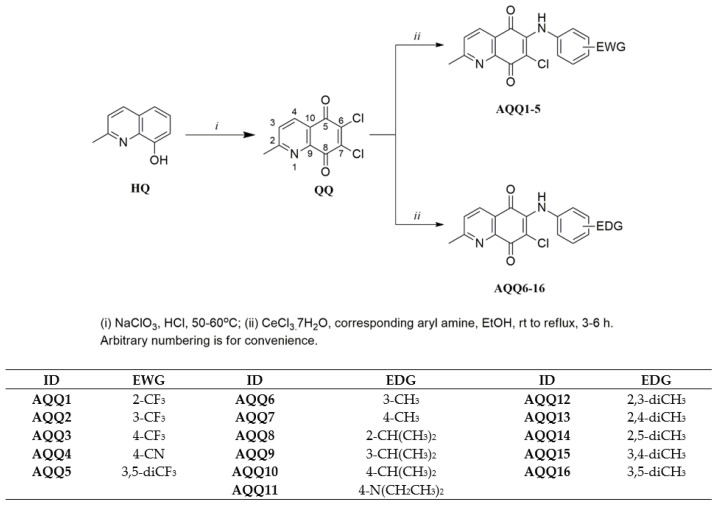
Preparation of two subseries of the AQQs.

**Figure 3:**
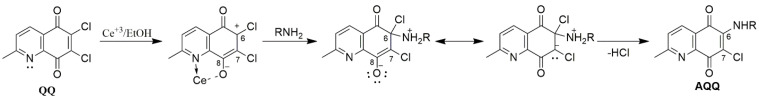
Preferential nucleophilic substitution with the addition of CeCl_3_·7H_2_O at C-6.

The target AQQs (**AQQ1**–**16**) were synthesized using the synthetic sequence illustrated in Scheme 1. While Petrow and Sturgeon reported the synthesis of QQs in five steps, we preferred the approach used by Shaikh et al. and synthesized the precursor (6,7-dichloro-2-methyl-5,8-quinolinequinone, **QQ**) in one step using the commercially available 8-hydroxy-2-methyl-quinoline (**HQ**) with sodium chlorate in concentrated HCl solution [67] with minor modifications. Since the precursor (2) has two asymmetric chlorine atoms at the C6 and C7 positions, in principle, two alternative pathways can produce the two isomers [68]. After the first step, the AQQs (**AQQ1**–**16**) containing EWG or EDG were obtained by the regioselective amination of the acceptor quinone nucleus (**QQ**) at the C6 position with corresponding aryl amines in the presence of a Lewis acid such as CeCl_3_·7H_2_O as catalyst according to the previously reported literature [69]. Particularly, the two lone pairs of electrons on the nitrogen atom of pyridine moiety and the oxygen atom of quinone moiety as shown in Figure 3 bonded to the Ce(III) ion via the chelation, reducing the electron density of C6 [68,70]. Additionally, the solvents used in this method like ethanol or water promoted the substitution with the chlorine atom linked at C6 position [68]. As expected, the observed regioselectivity for products (**AQQ1**–**16**) aligns with the report published by Ryu, Choi, and Kim [71] with perfect compatibility.

The regioselective amination of QQs at the C6 position was performed based on the nucleophilic substitution to electrophilic 1,4-quinone (Scheme 1) [69]. To synthesize the first subseries of a series of the AQQs, **AQQ1–5** were synthesized by amination of 6,7-dichloro-2-methyl-5,8-quinolinequinone (**QQ**) in the presence of CeCl_3_·7H_2_O with the corresponding aryl amines containing EWG such as trifluoromethyl and cyano groups (-CF_3_ and -CN) at different positions. The second subseries of the AQQs (**AQQ6**–**16**) were synthesized by following the same method using the corresponding aryl amines containing EDG such as alkyl and substituted amino groups (-R and -NR_2_) at different positions in ethanol. All AQQs were isolated as pure molecules by column chromatography using silica gel. The structures of the AQQs were characterized by versatile modern spectral techniques, including Fourier-transform infrared spectroscopy (FTIR), ^1^H nuclear magnetic resonance (NMR), ^13^C NMR, and high-resolution mass spectrometry (HRMS).

**Figure 4:**
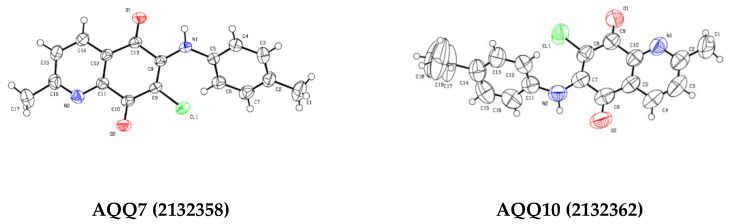
ORTEP drawings of the **AQQ7** and **AQQ10,** the atom numbering at 50% probability level.

The regioselective amination of QQs at the C6 position was confirmed by X-ray crystallographic studies [69,72]. The single-crystal X-ray crystallographic analysis of the AQQs provided the molecular structures of **AQQ7** and **AQQ10**. The ORTEP diagrams are shown in Figure 4. These QQs (**AQQ7** and **AQQ10**) were dissolved in ethanol and stored for one week for crystallization to yield good quality crystals. X-ray diffraction analyses to confirm the regioselectivity of the displacement reactions showed that **AQQ7** and **AQQ10** both contain an aromatic amino group at the 6th position.

Figure 4 presents ORTEP drawings of the **AQQ7** and **AQQ10** at 50% probability level and atom numbering of the crystals. The crystallographic and structure refinement data for the **AQQ7** and **AQQ10** are summarized in Table 1. When the **AQQ7** crystallized in the monoclinic crystal system (space group P 1 21/c 1) with the unit cell parameters a = 4.8838 (5), b = 25.878 (3), c = 11.6611 (12), α, γ = 90°, β = 98.331 (2)°, the **AQQ10** crystallized in the trigonal crystal system (space group R 3 c) with the unit cell parameters a = 28.573 (6), b = 28.573 (6), c = 10.771 (3), α, β = 90°, γ = 120°. Unit cells contain four molecules in the **AQQ17** and eighteen molecules in the **AQQ10** (Table 1). The average value of the distances between the C and N atoms of the quinoline ring in the **AQQ7** and **AQQ10** is 1.33 Å. The lengths of the C=O bonds on the benzoquinone ring of both QQs are about 1.22 Å, while the C=C bond lengths are around 1.38 Å, confirming that these are typical double bonds. The lengths of the C-Cl bonds in the compounds are 1.73 Å. The bond lengths between the sp^2^-hybridized C atoms of the benzene and quinoline ring are about 1.38 Å, confirming the aromatic character of the rings. The fact that the single bond length between the sp^3^-hybridized carbon atom of the methyl substituent in the benzene ring and the sp^2^-hybridized carbon atoms in the benzene ring is 1.49 Å, which is higher than the values between the sp^2^-hybridized carbon atoms, is also compatible with the hybridization theory (Table S1 in Supplementary Material). The C-C-C angles of the benzoquinone, quinoline, and benzene rings and the C-C-O angles of the benzoquinone ring in two QQs are very close to 120°, which supports the structures involving sp^2^-hybridized atoms. It is known that methyl groups generally adopt an ideal tetrahedral geometry with H-C-H and C-C-H bond angles of 109.5°, and the methyl group shows an angle of 109.5° in compounds (Table S2 in Supplementary Material). The torsion angles of the **AQQ7** and **AQQ10** are provided as Table S3 in the Supplementary Material. The crystal structure of the **AQQ7** is stabilized by hydrogen bonds, which are formed between the C-H groups as H-bond donors (D) and O atom as H-bond acceptors (A). The crystal structure of the **AQQ10** is stabilized by hydrogen bonds, which are formed between the N-H groups as H-bond donors (D) and O or Cl atoms as H-bond acceptors (A). All parameters of the hydrogen bonds are shown in Table S4 in Supplementary Material.

### 2.2. Antimicrobial Activity

#### 2.2.1. Determination of Minimum Inhibitory Concentrations (MIC)

Before biological testing, the purity of the AQQs was analyzed using high-performance liquid chromatography (HPLC) with hexane/2-propanol = 95:5 as the mobile phase at a flow rate of 1.0 mL/min. The purity of all analogs was ≥95%. Their chromatograms are provided as a Supplementary File (Figures S1–S16). The antibacterial activity of the AQQs was evaluated by determining their minimum inhibitory concentration (MIC) against Gram-negative and Gram-positive bacterial strains. In order to heuristically realize the antibacterial profile of the AQQs, the MIC values of these molecules summarized in Table 2 were compared with those of commercially available reference drug Ciprofloxacin. Gram-negative bacteria are reportedly more resistant to most drugs; unfortunately, these bacteria were also resistant to all of our molecules. The results indicated that the first subseries of the AQQs (**AQQ1–5**) containing EWG exhibited a low activity against Gram-positive bacterial strains with a MIC of more than 39.06 μg/mL. In contrast, the second subseries of the AQQs (**AQQ8**–**16**) containing EDG displayed a strong activity against Gram-positive bacterial strains, especially *S. aureus* with a MIC value of 1.22 μg/mL (close to that of Ciprofloxacin) and *E. faecalis* with a MIC value of 9.76 μg/mL (**AAQ9**) (the best value to that of Ciprofloxacin). Furthermore, **AQQ8** and **AQQ10** showed strong inhibitory potency against *E. faecalis* with a MIC value of 19.53 μg/mL. Especially, the MIC values of the AQQs (**AQQ8**–**10** and **AQQ12**–**14**) for *S. aureus* were close to that of Ciprofloxacin. Analyzing the antifungal activity revealed that **AQQ4** had the best inhibitory activity with a MIC value of 1.22 μg/mL (4-fold superior to that of Clotrimazole) against *C. albicans*. **AQQ4** and **AQQ9** exhibited the strongest antifungal profile among the AQQs against *C. parapsilosis* with MIC values of 2.44 and 9.76 μg/mL, respectively (Table 3). The antimicrobial activity of the AQQs is significantly affected by the electron density of the substituent(s). While the first subseries of the AQQs (**AQQ1–5**) containing EWG did not show any significant antibacterial activity against the tested bacteria, one from this subseries (**AQQ4**) displayed the highest antifungal activity against *C. albicans* and *C. parapsilosis*. In contrast, most compounds of the second subseries of the AQQs (**AQQ6**–**16**) containing EDG exhibited significant antibacterial activity against *S. aureus*.

**Figure 5:**
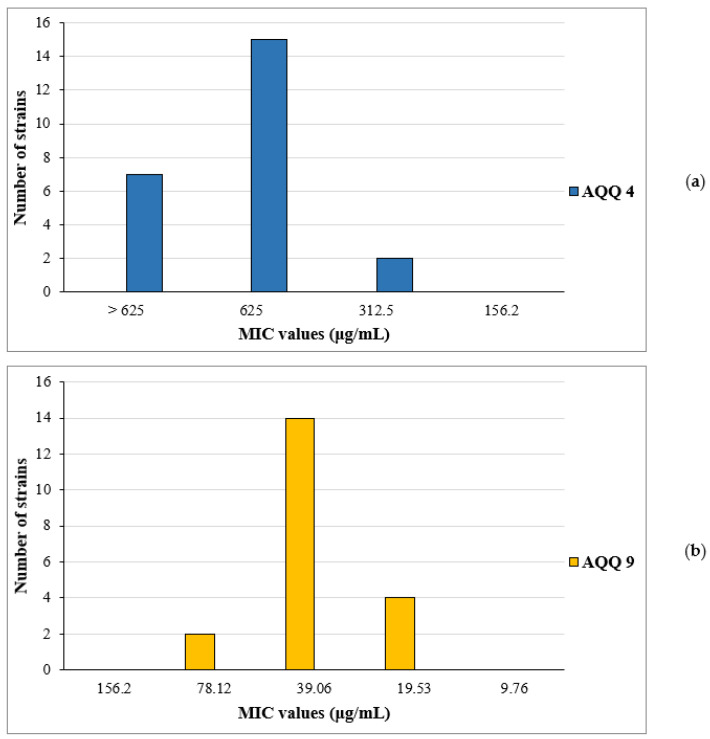
MIC distribution of **AQQ4** and **AQQ9**; (**a**) the MIC distribution of **AQQ4** against 24 clinically obtained *C. albicans* and *non-albicans* strains, (**b**) the MIC distribution of **AQQ9** against 20 clinically obtained *MRSA* isolates.

Based on the MIC results for standard American Type Culture Collection (ATCC) isolates, we also purposed to examine the potential antimicrobial activities of selected active molecules **AQQ4** and **AQQ9** against clinically obtained *C. albicans* (and also *non-albicans*) and methicillin-resistant strains of *S. aureus*, respectively. The in vitro activities of the **AQQ4** against 24 clinical isolates of *C. albicans,* and *non-albicans* are summarized in Figure 5a, and the in vitro activities of the **AQQ9** against 20 clinically methicillin-resistant *S. aureus* (*MRSA)* isolates are summarized in Figure 5b. While the in vitro activity to **AQQ4** was not effective as the MIC results of standard ATCC isolates, the in vitro antibacterial activity of **AQQ9** seemed to be good with MIC_90_ of 39.06 μg/mL.

#### 2.2.2. Structure-Activity Relationships (SARs) Study for Biological Evaluation

Herein, initially, 1,4-quinone fused with pyridine moiety containing corresponding aryl amine was used as a prototype to explore new lead molecules and obtain new antibacterial and/or antifungal agent candidates. Subsequently, the substituent(s) such as EWG (-CF_3_ and -CN) and EDG (-R and -NR_2_) at different positions (2-, 3-, or 4-) attached to the phenyl moiety within the aryl amine were analyzed. The biological testing results suggested that we could deduce some valuable insights about the SAR from Table 2 and Table 3. At first, the MIC values of **AQQ1–5** containing EWG against Gram-positive bacterial strains compared to the **AQQ6–16** containing EDG indicates that the EDG is essential for biological potency. The effect of the position of the substituent at the phenyl moiety was then investigated. However, when EDG, such as alkyl or substituted amino group(s), were inserted in the phenyl moiety of aryl amine at different positions, the activity increased significantly, particularly against *S. aureus*. We observed the best antibacterial potency against *S. aureus* when the AQQ analogs were substituted with the alkyl group(s) (weak EDG). As expected, the nature of the alkyl chain in aryl amine dramatically affected the activity. We noticed a significant increase in inhibitory activity when the length of the alkyl chain in aryl amine was changed from methyl to isopropyl group. However, the biological potency against *S. aureus* increased again after inserting the additional weak EDG (-diCH_3_) in aryl amine. We also concluded that strong EDG (-NR_2_) insertion did not significantly affect biological activity since the biological potency dramatically decreased with strong EDG. Concerning the antifungal activity, the AQQs containing EWG are more active than the AQQs containing EDG. Obviously, there is a simple correlation between antibacterial/antifungal activity and electron density in the phenyl moiety of the aryl amine.

#### 2.2.3. Time-Kill Kinetic Study

**Figure 6:**
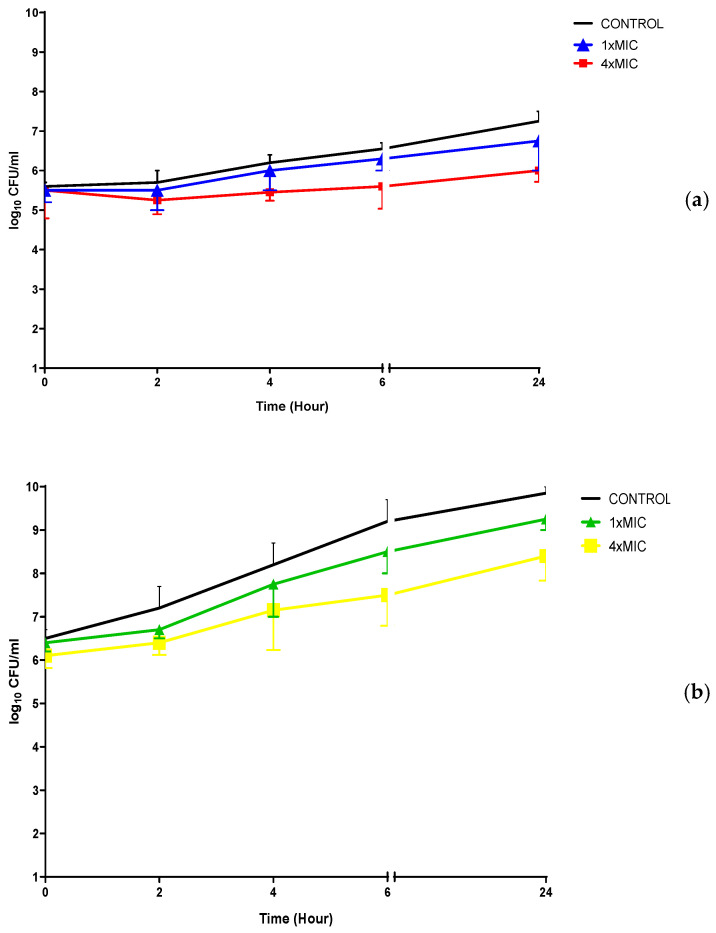
Time-kill determinations for clinically resistant (**a**) *C. albicans* and (**b**) *MRSA* isolates after treatment with **AQQ4** and **AQQ9** at 1× and 4× MIC, respectively. The *x*-axis represents the killing time, and the *y*-axis represents the logarithmic *C. albicans* and *MRSA* survival, respectively.

The two most active AQQs (**AQQ4** and **AQQ9**) were selected from the library after analyzing in vitro antimicrobial activity for further investigation of the mode of the action. Time-kill kinetic studies (TKS) for **AQQ4** and **AQQ9** were performed on one (1) clinically obtained *MRSA* and one (1) *C. albicans* isolates, and the results are given in Figure 6, respectively.

The results of the time kill curve studies did not reveal bactericidal activity (with a 3-log_10_ kill determined) for the studied strains at 1× and 4× MIC concentrations within 24 h, as shown in Figure 6. When **AQQ4** and **AQQ9** were used at 4× MIC concentrations, there was only about 1 log_10_ reduction in the viable microorganism cell count within 24 h.

#### 2.2.4. Evaluation of the In Vitro Antibiofilm Activity

Besides the development of antimicrobial resistance through the evolution of internal machinery, bacteria and fungi also develop biofilm to evade antibiotics. Biofilms are clusters of microorganisms embedded in an extracellular polymeric matrix that is self-produced, and they are commonly attached to living or nonliving surfaces and may be widespread in nature, hospital settings, and industry [73,74]. Biofilm formation contributes significantly to the morbidity and mortality associated with infectious diseases because of the persistent nature of infection and the evolution of antibiotic resistance [74].

**Figure 7:**
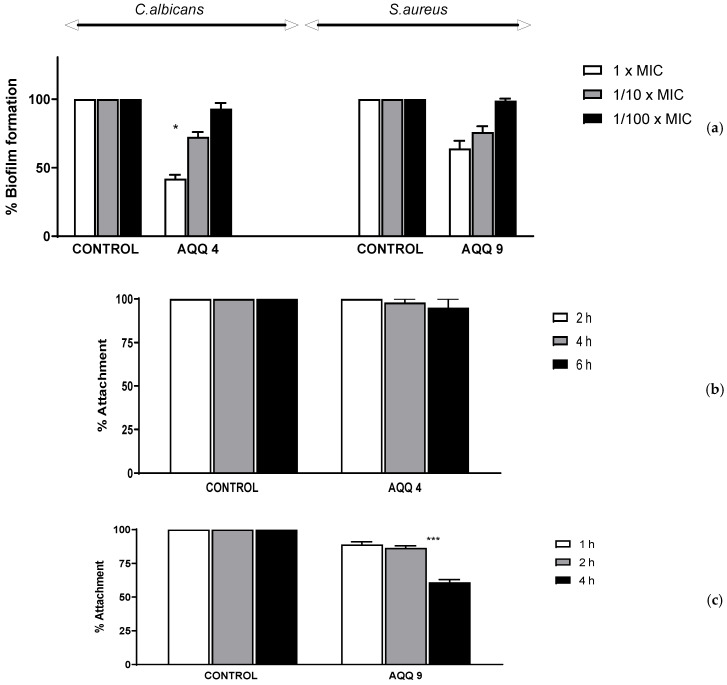
Inhibition of *C. albicans* and *MRSA*, respectively; (**a**) biofilm formation in each well contained 1×, 1/10×, or 1/100× MIC of molecules and an inoculum of 1 × 10^6^–1 × 10^7^ CFU/200 μL, incubated for 24 h at 37 °C for *C. albicans* and *MRSA*. (**b**,**c**) surface attachment to the wells contained 1/10× MIC of molecules and an inoculum of 1 × 10^6^ CFU/200 μL, incubated for 2, 4, or 6 h at 37 °C for *C. albicans* and incubated for 1, 2, or 4 h at 37 °C for *MRSA*. Control bars indicate microorganisms without molecules accepted as 100%. Six wells were used for the tested molecules. Each experiment is representative of two independent tests. All differences between the control and molecules treated biofilms were statistically significant (* not significant, *** *p* < 0.0001).

When the 1/10× MICs of tested molecules were examined for 1, 2, or 4 h at 37 °C for *MRSA*’s and for 2, 4, or 6 h at 37 °C for *C. albicans*’s, adherence to the wells of tissue culture microtiter plates, the tested agents did not inhibit biofilm attachment processes. Inhibition adhesion rates showed a time-dependent effect only for *C. albicans* with **AQQ4**. Upon evaluating the % biofilm formation of the studied strains, the rates of biofilm formation inhibition were found to be dependent on concentration; the highest inhibition rates were shown at 1× MICs for the tested molecules, as expected (Figure 7). 

#### 2.2.5. In Silico Molecular Interaction Studies

A summary of key results from the docking study is provided in Table 4. We selected the top two ligands, **AQQ4** and **AQQ9**, and performed molecular docking against the three target proteins. 

**Figure 8:**
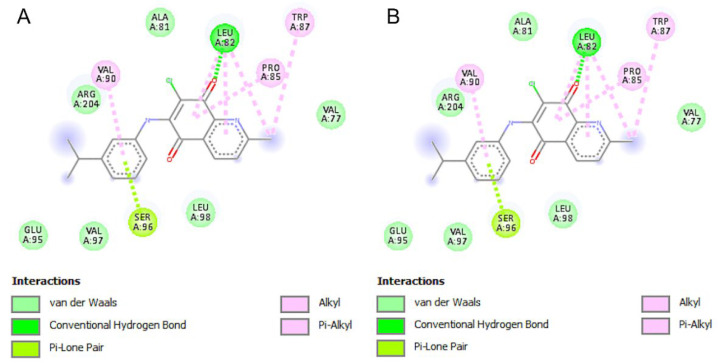
Schematic 2-D representation of protein–ligand interactions between the key residues of DmsD (PDB: 3CW0) binding pocket with (**A**) **AQQ4** (left side) and (**B**) **AQQ9** (right side).

**AQQ4** showed one hydrogen bonding interaction and 11 hydrophobic interactions in the active site of 3CW0. It appeared to occupy the proposed binding pocket with a relatively good binding energy of −5.36 Kcal/mol and an inhibition constant (Ki) of 117.62 µM; these results were relatively inferior to **AQQ9** (Table 4). **AQQ9** was predicted to form one hydrogen bond and 10 hydrophobic interactions with the pocket residues with an inhibition constant of 70.88 µM. In these redox enzyme maturation proteins, the conserved sequence motifs Asp126 and His127 have been shown to be crucial for function. We did not find a direct interaction between these motifs and our ligand (Figure 8).

**Figure 9:**
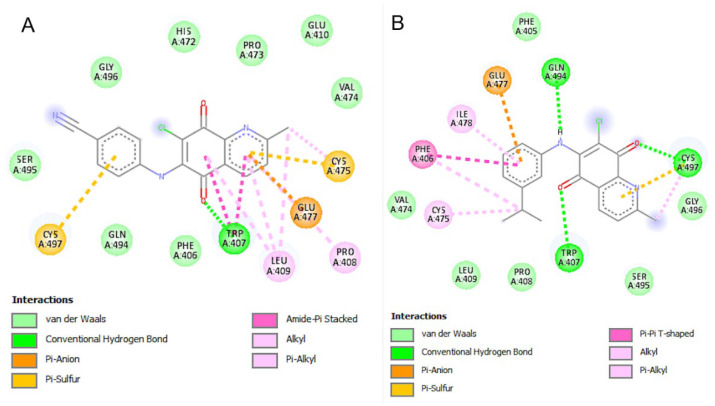
Schematic 2-D representation of protein–ligand interactions between the key residues of Thioredoxin reductase 1 (PDB: 3EAN) binding pocket with (**A**) **AQQ4** and (**B**) **AQQ9**.

Both **AQQ4** and **AQQ9** appeared to form stronger binding interactions with the active pocket of 3EAN with binding energies superior to those in 3CW0 (−7.88 and −8.43, respectively), suggesting the possibility of a better binding profile (Table 4). Additionally, the predicted inhibition constant for these compounds against 3EAN was in the sub-micromolar range (Ki = −1.69 and 0.664 µM, respectively), with **AQQ9** showing a stronger profile. Our ligand appeared to form a favorable anion-π interaction with GLU477 and hydrophobic interaction with HIS472, which are among the three key amino acids from the catalytic triad of TrxR1 (Figure 9). 

**Figure 10:**
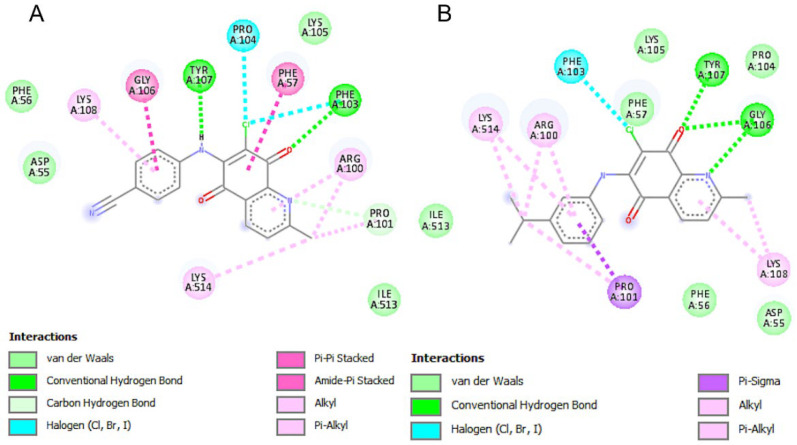
Schematic 2-D representation of protein–ligand interactions between the key residues of Polynucleotide phosphorylase (PNPase) (PDB: 5XEX) binding pocket with (**A**) **AQQ4** (left side) and (**B**) **AQQ9** (right side).

The activity profiles of **AQQ4** and **AQQ9** against 5XEX were similar to those observed in 3CW0, with low binding energies (−8.06 and −7.93 Kcal/mol, respectively) and sub-micromolar inhibition constants (1.24 and 1.53 µM, respectively) (Table 4). Although the binding energy and inhibition constant were the lowest for **AQQ9** among these compounds, **AQQ4** formed two hydrogen bonds and appeared to form a π-π stacking interaction with the LYS514 group through the quinoline (Figure 10).

The molecular docking results align with the results obtained from experimental studies where the first subseries with electron-withdrawing groups were relatively less potent than the series with electron-donating groups. Furthermore, the compounds showed good binding affinities to both 3CW0 and 5XEX, which are key to the survival of *S. aureus,* which was also noted in the in vitro assays. The molecular docking results also align well with the SAR evaluation, wherein the EDG groups were proposed to enhance the potency. The good activity in vitro of the compounds is also rationalized by the in silico findings as the tested compounds interacted with key amino acids in the binding site.

## 3. Experimental

### 3.1. Chemicals and Apparatus

All commercially available starting materials, reagents, and solvents were purchased from various commercial sources with a minimum purity of 95% and used without further purification. All reactions were monitored for completion by thin-layer chromatography (TLC) using Merck DC-plates (aluminum-based, silica gel 60 F254) purchased from Merck KGaA and visualized by UV light (254 nm). Column chromatography was performed with a silica gel 60 (63–200 µm particle size) purchased from Merck with the appropriate solvent system as eluents. Melting points (mp) were measured in a capillary tube in an electrical melting point (B-540, Büchi) and are uncorrected. FTIR spectra were recorded with Alpha T FTIR spectrometer with a single reflection diamond ATR module. ^1^H NMR spectra were recorded on Bruker NMR spectrometers at 500 MHz frequency, and ^13^C NMR were recorded on Bruker NMR spectrometers at 125 MHz frequency in the specified deuterated solvent, respectively. Chemical shifts were signified in parts per million (ppm) in CDCl_3_ or DMSO-*d*_6_ and coupling constants (J) are in hertz (Hz). High-resolution mass spectra electrospray ionization (HRMS-ESI) was obtained on a Waters SYNAPT G1 MS. The HPLC analyses were accomplished with Shimadzu/DGU-20A5 HPLC apparatus fitted with a 25-cm Chiralpac AD-H chiral column. The precursor, 6,7-dichloro-2-methyl-5,8-quinolinequinone, **QQ**, was synthesized using the reported method in the literature [67].

### 3.2. X-ray Diffraction Analysis

Data for the single crystal compounds were obtained with Bruker APEX II QUAZAR three-circle diffractometer. Indexing was performed using APEX2 [75]. Data integration and reduction were carried out with SAINT [76]. Absorption correction was performed by the multi-scan method implemented in SADABS [77]. The Bruker SHELXTL [78] software package was used for structures solution and structures refinement. Aromatic C-bound and N-bound hydrogen atoms were positioned geometrically and refined using a riding mode. Crystal structure validations and geometrical calculations were performed using the Platon software [79]. Mercury software [80] was used for the visualization of the .cif files. The crystallographic and structure refinement data are summarized in Table 1. The selected bond lengths, bond angles, torsion angles, hydrogen bond distances, and angles are given in the Supplementary Material. The crystallographic data have been deposited at the Cambridge Crystallographic Data Center, and CCDC reference numbers are 2132358 and 2132362 for **AQQ7** and **AQQ10**. The data can be obtained available free of charge from http://www.ccdc.cam.ac.uk/conts/retrieving.html or from the Cambridge Crystallographic Data Center (CCDC), 12 Union Road, Cambridge CB2 1EZ, UK; Fax: +44-(0)1223336033; email: deposit@ccdc.cam.ac.uk.

### 3.3. Procedure for the Synthesis of the Methyl Quinolinequinone (***QQ***)

#### 6,7-Dichloro-2-methyl-5,8-quinolinequinone (**QQ**)

6,7-Dichloro-2-methyl-5,8-quinolinequinone (**QQ**) [67] was prepared according to the literature from 8-hydroxy-2-methyl-quinoline and sodium chlorate in concentrated HCl in 12% yield. Mp 179–180 °C (Lit. 180–181 °C). ^1^H NMR (500 MHz, CDCl_3_) *δ* (ppm): 8.31 (d, *J* = 8.30 Hz, 1H, CH_aromatic_), 7.53 (d, *J* = 8.30 Hz, 1H, CH_aromatic_), 2.73 (s, 3H, CH_3_). ^13^C NMR (125 MHz, CDCl_3_) *δ* (ppm): 174.6, 173.5 (>C=O), 165.1, 145.3, 142.9, 141.8, 134.6, 127.2, 125.2 (C_aromatic_ and C_q_), 24.3 (CH_3_). MS (+ESI) *m*/*z* (%): 242 (100, [M + H]^+^), 240 (11, [M − H]^+^). Anal. Calcd. for C_10_H_5_Cl_2_NO_2_ (240.97).

### 3.4. General Procedure for the Synthesis of the Aminated Quinolinequinones *(**AQQ1**–**16**)*

A suspension of the **QQ** (0.250 g, 1.03 mmol) and CeCl_3_·7H_2_O (0.421 g, 1.13 mmol, 1.1 equiv) in ethanol was stirred at room temperature for 1 h. Then, to that solution of the **QQ** and CeCl_3_·7H_2_O was successively added the corresponding amines (1.13 mmol, 1.1 equiv) in ethanol and stirred for 3–16 h until consumption of the **QQ**. After evaporation of the solvent, the residue was dissolved with CH_2_Cl_2_ (50 mL), and the solution was washed sequentially with water (3 × 30 mL). The organic layer was dried over CaCl_2_, filtered, and concentrated under reduced pressure. The reaction crude was purified on a silica gel column chromatography using the eluent system to give the corresponding **AQQ**.

#### 3.4.1. 7-Chloro-2-methyl-6-((2-(trifluoromethyl)phenyl)amino)-5,8-quinolinequinone (**AQQ1**)

Obtained from the mixture (**QQ** and CeCl_3_·7H_2_O) and 2-(trifluoromethyl)aniline (0.182 g, 1.13 mmol) according to the general procedure, the title compound (**AQQ1**) was purified by column chromatography as an orange solid. Yield: 7%, 192–195 °C. Purity: 100% (Retention time: 3.66 min). FTIR (ATR) υ (cm^−1^): 3359 (NH), 3070, 3040 (CH_aromatic_), 1667 (>C=O), 1603, 1575, 1507, 1476, 1457, 1374, 1316, 1280, 1224, 1204, 1180, 1169, 1145, 1126, 1112, 1058, 1036. ^1^H NMR (500 MHz, CDCl_3_) *δ* (ppm): 8.33 (d, *J* = 8.0 Hz, 1H, CH_aromatic_), 7.71 (d, *J* = 7.6 Hz, 1H, CH_aromatic_), 7.64 (br s, 1H, NH), 7.56 (t, *J* = 7.7 Hz, 1H, CH_aromatic_), 7.52 (d, *J* = 8.0 Hz, 1H, CH_aromatic_), 7.37 (t, *J* = 7.7 Hz, 1H, CH_aromatic_), 7.14 (d, *J* = 8.0 Hz, 1H, CH_aromatic_), 2.81 (s, 3H, CH_3_). ^13^C NMR (125 MHz, CDCl_3_) *δ* (ppm): 179.5, 176.1 (>C=O), 166.2, 147.7, 140.9, 135.5, 135.0, 131.8, 127.5, 127.0, 126.5 (q, ^3^*J_C-F_* = 5.1 Hz), 126.2, 124.7 (q, ^2^*J_C-F_* = 30.1 Hz), 123.7 (q, ^1^*J_C-F_* = 273.0 Hz), 122.6, 116.9, 110.0 (C_aromatic_ and C_q_), 25.5 (CH_3_). HRMS(+ESI) *m*/*z* calcd for C_17_H_11_ClF_3_N_2_O_2_ [M + H]^+^: 367.0461; found: 367.0460.

#### 3.4.2. 7-Chloro-2-methyl-6-((3-(trifluoromethyl)phenyl)amino)-5,8-quinolinequinone (**AQQ2**)

Obtained from the mixture (QQ and CeCl_3_·7H_2_O) and 3-(trifluoromethyl)aniline (0.182 g, 1.13 mmol) according to the general procedure, the title compound (AQQ2) was purified by column chromatography as a red solid. Yield: 71%, 246–248 °C. Purity: 98% (Retention time: 3.67 min). FTIR (ATR) υ (cm^−1^): 3211 (NH), 3030 (CH_aromatic_), 1676 (>C=O), 1573, 1515, 1474, 1454, 1332, 1307, 1290, 1224, 1198, 1158, 1114, 1101, 1068, 1036. ^1^H NMR (500 MHz, CDCl_3_) δ (ppm): 8.34 (d, *J* = 8.0 Hz, 1H, CH_aromatic_), 7.67 (br s, 1H, NH), 7.56–7.45 (m, 3H, CH_aromatic_), 7.35 (s, 1H, CH_aromatic_), 7.30–7.22 (m, 1H, CH_aromatic_), 2.82 (s, 3H, CH_3_). ^13^C NMR (125 MHz, CDCl_3_) δ (ppm): 179.8, 176.1 (>C=O), 166.3, 147.8, 140.4, 137.7, 135.0, 129.0, 127.1, 124.7, 122.3, 120.7, 117.1, 110.0 (C_aromatic_ and C_q_), 25.5 (CH_3_). HRMS(+ESI) *m*/*z* calcd for C_17_H_10_ClF_3_N_2_O_2_ [M]^+^: 366.0383; found: 366.0386.

#### 3.4.3. 7-Chloro-2-methyl-6-((4-(trifluoromethyl)phenyl)amino)-5,8-quinolinequinone (**AQQ3**)

Obtained from the mixture (**QQ** and CeCl_3_·7H_2_O) and 4-(trifluoromethyl)aniline (0.182 g, 1.13 mmol) according to the general procedure, the title compound (**AQQ3**) was purified by column chromatography as a red solid. Yield: 74%, 249–251 °C. Purity: 100% (Retention time: 3.66 min). FTIR (ATR) υ (cm^−1^): 3208 (NH), 1672 (>C=O), 1651, 1571, 1520, 1494, 1409, 1370, 1320, 1289, 1224, 1162, 1107, 1064. ^1^H NMR (500 MHz, *DMSO-d_6_*) *δ* (ppm): 9.54 (br s, 1H, NH), 8.29 (d, *J* = 8.0 Hz, 1H, CH_aromatic_), 7.68 (d, *J* = 8.1 Hz, 1H, CH_aromatic_), 7.65 (d, *J* = 8.5 Hz, 2H, CH_aromatic_), 7.27 (d, *J* = 8.5 Hz, 2H, CH_aromatic_), 2.67 (s, 3H, CH_3_). ^13^C NMR (125 MHz, *DMSO-d_6_*) *δ* (ppm): 180.2, 176.2 (>C=O), 164.7, 147.6, 143.4, 142.6, 135.2, 127.5, 126.1, 125.6 (q, ^3^*J_C-F_* = 3.5 Hz), 124.9 (q, ^1^*J_C-F_* = 271.4 Hz), 123.8 (q, ^2^*J_C-F_* = 31.8 Hz), 123.1, 119.5 (C_aromatic_ and C_q_), 25.1 (CH_3_). HRMS(+ESI) *m*/*z* calcd for C_17_H_10_ClF_3_N_2_O_2_ [M]^+^: 366.0383; found: 366.0386.

#### 3.4.4. 7-Chloro-6-((4-(cyano)phenyl)amino)-2-methyl-5,8-quinolinequinone (**AQQ4**)

Obtained from the mixture (**QQ** and CeCl_3_·7H_2_O) and 4-cyanoaniline (0.133 g, 1.13 mmol) according to the general procedure, the title compound (**AQQ4**) was purified by column chromatography as a red solid. Yield: 63%, 283–284 °C. Purity: 97% (Retention time: 3.66 min). FTIR (ATR) υ (cm^−1^): 3185 (NH), 3081 (CH_aromatic_), 2227 (CN), 1673 (>C=O), 1655, 1572, 1512, 1493, 1411, 1367, 1307, 1293, 1258, 1226, 1206, 1171, 1145, 1108, 1036. ^1^H NMR (500 MHz, CDCl_3_) *δ* (ppm): 8.35 (d, *J* = 8.0 Hz, 1H, CH_aromatic_), 7.67 (d, *J* = 8.3 Hz, 2H, CH_aromatic_), 7.64 (br s, 1H, NH), 7.55 (d, *J* = 8.0 Hz, 1H, CH_aromatic_), 7.11 (d, *J* = 8.4 Hz, 2H, CH_aromatic_), 2.83 (s, 3H, CH_3_). ^13^C NMR (125 MHz, CDCl_3_) *δ* (ppm): 179.7, 176.0 (>C=O), 166.5, 147.6, 141.2, 139.9, 135.1, 132.6, 127.3, 124.8, 123.1, 119.2, 118.5, 110.4, 108.2 (C_aromatic_ and C_q_), 25.5 (CH_3_). HRMS(+ESI) *m*/*z* calcd for C_17_H_10_ClN_3_O_2_ [M]^+^: 323.0462; found: 323.0465.

#### 3.4.5. 6-((3,5-Bis(trifluoromethyl)phenyl)amino)-7-chloro-2-methyl-5,8-quinolinequinone (**AQQ5**) 

Obtained from the mixture (**QQ** and CeCl_3_·7H_2_O) and 3,5-bis(trifluoromethyl)aniline (0.259 g, 1.13 mmol) according to the general procedure, the title compound (**AQQ5**) was purified by column chromatography as an orange solid. Yield: 19%, 197–198 °C. Purity: 97% (Retention time: 3.66 min). FTIR (ATR) υ (cm^−1^): 3227 (NH), 3033 (CH_aromatic_), 2959 (CH_aliphatic_), 1675 (>C=O), 1575, 1523, 1470, 1448, 1379, 1309, 1283, 1217, 1187, 1167, 1114, 1036. ^1^H NMR (500 MHz, CDCl_3_) *δ* (ppm): 8.27 (d, *J* = 8.0 Hz, 1H, CH_aromatic_), 7.65 (br s, 1H, NH), 7.63 (s, 1H, CH_aromatic_), 7.46 (d, *J* = 8.0 Hz, 1H, CH_aromatic_), 7.40 (s, 2H, CH_aromatic_), 2.73 (s, 3H, CH_3_). ^13^C NMR (125 MHz, CDCl_3_) *δ* (ppm): 179.6, 175.9 (>C=O), 166.5, 147.6, 139.9, 138.7, 135.1, 132.0 (q, ^2^*J_C-F_* = 33.9 Hz), 127.3, 124.8, 123.1, 122.9 (q, ^1^*J_C-F_* = 273.1 Hz), 118.6, 110.0 (C_aromatic_ and C_q_), 25.5 (CH_3_). HRMS(+ESI) *m*/*z* calcd for C_18_H_10_ClF_6_N_2_O_2_ [M + H]^+^: 435.0335; found: 435.0335.

#### 3.4.6. 7-Chloro-2-methyl-6-(m-tolylamino)-5,8-quinolinequinone (**AQQ6**)

Obtained from the mixture (**QQ** and CeCl_3_·7H_2_O) and *m-*toluidine (0.121 g, 1.13 mmol) according to the general procedure, the title compound (**AQQ6**) was purified by column chromatography as a purple solid. Yield: 63%, mp 192–194 °C. Purity: 100% (Retention time: 3.77 min). FTIR (ATR) υ (cm^−1^): 3187 (NH), 3022 (CH_aromatic_), 2917, 2856 (CH_aliphatic_), 1678 (>C=O), 1644, 1570, 1557, 1517, 1485, 1463, 1368, 1319, 1287, 1225, 1160, 1141, 1101, 1041. ^1^H NMR (500 MHz, CDCl_3_) *δ* (ppm): 8.23 (d, *J* = 8.0 Hz, 1H, CH_aromatic_), 7.56 (br s, 1H, NH), 7.41 (d, *J* = 8.0 Hz, 1H, CH_aromatic_), 7.17 (t, *J* = 7.9 Hz, 1H, CH_aromatic_), 6.97 (d, *J* = 7.6 Hz, 1H, CH_aromatic_), 6.82 (d, *J* = 7.3 Hz, 2H, CH_aromatic_), 2.72 (s, 3H, CH_3_), 2.30 (s, 3H, CH_3_). ^13^C NMR (125 MHz, CDCl_3_) *δ* (ppm): 180.1, 176.2 (>C=O), 166.1, 148.1, 140.9, 138.5, 137.0, 134.9, 128.3, 126.7, 124.9, 124.7, 121.4, 115.6, 110.0 (C_aromatic_ and C_q_), 25.5, 21.4 (CH_3_). HRMS(+ESI) *m*/*z* calcd for C_17_H_13_ClN_2_O_2_ [M]^+^: 312.0666; found: 312.0670.

#### 3.4.7. 7-Chloro-2-methyl-6-(p-tolylamino)-5,8-quinolinequinone (**AQQ7**)

Obtained from the mixture (**QQ** and CeCl_3_·7H_2_O) and *p-*toluidine (0.121 g, 1.13 mmol) according to the general procedure, the title compound (**AQQ7**) was purified by column chromatography as a purple solid. Yield: 47%, mp 213–215 °C. Purity: 100% (Retention time: 3.66 min). FTIR (ATR) υ (cm^−1^): 3187 (NH), 3026 (CH_aromatic_), 2919 (CH_aliphatic_), 1675 (>C=O), 1645, 1571, 1518, 1498, 1405, 1367, 1303, 1288, 1226, 1206, 1143, 1102, 1038. ^1^H NMR (500 MHz, CDCl_3_) *δ* (ppm): 8.21 (d, *J* = 8.0 Hz, 1H, CH_aromatic_), 7.57 (br s, 1H, NH), 7.39 (d, *J* = 8.0 Hz, 1H, CH_aromatic_), 7.08 (d, *J* = 7.9 Hz, 2H, CH_aromatic_), 6.91 (d, *J* = 7.9 Hz, 2H, CH_aromatic_), 2.70 (s, 3H, CH_3_), 2.29 (s, 3H, CH_3_). ^13^C NMR (125 MHz, CDCl_3_) *δ* (ppm): 180.1, 176.1 (>C=O), 166.0, 148.1, 141.0, 135.9, 134.9, 134.5, 129.1, 126.7, 124.4, 115.0, 110.0 (C_aromatic_ and C_q_), 25.5, 21.1 (CH_3_). HRMS(+ESI) *m*/*z* calcd for C_17_H_13_ClN_2_O_2_ [M]^+^: 312.0666; found: 312.0667.

#### 3.4.8. 7-Chloro-6-((2-isopropylphenyl)amino)-2-methyl-5,8-quinolinequinone (**AQQ8**)

Obtained from the mixture (**QQ** and CeCl_3_·7H_2_O) and 2-isopropylaniline (0.153 g, 1.13 mmol) according to the general procedure, the title compound (**AQQ8**) was purified by column chromatography as a dark red oil. Yield: 62%. Purity: 96% (Retention time: 3.65 min). FTIR (ATR) υ (cm^−1^): 3326 (NH), 3070 (CH_aromatic_), 2964, 2930, 2885 (CH_aliphatic_), 1664 (>C=O), 1571, 1498, 1469, 1450, 1313, 1286, 1258, 1220, 1144, 1101, 1080, 1038. ^1^H NMR (500 MHz, CDCl_3_) *δ* (ppm): 8.29 (d, *J* = 5.2 Hz, 1H, CH_aromatic_), 7.49 (br s, 1H, NH), 7.54–7.43 (m, 1H, CH_aromatic_), 7.36–7.24 (m, 2H, CH_aromatic_), 7.17 (s, 1H, CH_aromatic_), 7.02 (d, *J* = 6.5 Hz, 1H, CH_aromatic_), 3.10–3.27 (m, 1H, CH(CH_3_)_2_), 2.78 (s, 3H, CH_3_), 1.25 (s, 6H, CH_3_). ^13^C NMR (125 MHz, CDCl_3_) *δ* (ppm): 180.0, 176.0 (>C=O), 166.0, 148.1, 143.9, 141.8, 134.9, 134.8, 127.6, 127.0, 126.7, 125.9, 125.6, 124.6, 114.2 (C_aromatic_ and C_q_), 28.6 (CH), 25.5, 22.9 (CH_3_). HRMS(+ESI) *m*/*z* calcd for C_19_H_18_ClN_2_O_2_ [M + H]^+^: 341.1057; found: 341.1057.

#### 3.4.9. 7-Chloro-6-((3-isopropylphenyl)amino)-2-methyl-5,8-quinolinequinone (**AQQ9**)

Obtained from the mixture (**QQ** and CeCl_3_·7H_2_O) and 3-isopropylaniline (0.153 g, 1.13 mmol) according to the general procedure, the title compound (**AQQ9**) was purified by column chromatography as a purple solid. Yield: 51%, 114–116 °C. Purity: 100% (Retention time: 3.74 min). FTIR (ATR) υ (cm^−1^): 3297 (NH), 3059 (CH_aromatic_), 2959, 2922, 2867 (CH_aliphatic_), 1672 (>C=O), 1568, 1513, 1479, 1440, 1370, 1310, 1282, 1217, 1143, 1102, 1036. ^1^H NMR (500 MHz, CDCl_3_) *δ* (ppm): 8.27 (d, *J* = 7.7 Hz, 1H, CH_aromatic_), 7.75 (br s, 1H, NH), 7.47 (d, *J* = 7.8 Hz, 1H, CH_aromatic_), 7.25 (t, *J* = 7.3 Hz, 1H, CH_aromatic_), 7.07 (d, *J* = 7.2 Hz, 1H, CH_aromatic_), 6.91 (s, 2H, CH_aromatic_), 2.97–2.83 (m, 1H, CH(CH_3_)_2_), 2.76 (s, 3H, CH_3_), 1.32–1.17 (m, 6H, CH_3_). ^13^C NMR (125 MHz, CDCl_3_) *δ* (ppm): 180.1, 176.1 (>C=O), 166.0, 149.3, 148.0, 140.8, 136.9, 134.9, 128.3, 126.8, 124.7, 124.1, 122.5, 121.7, 115.2 (C_aromatic_ and C_q_), 33.9 (CH), 25.4, 23.8 (CH_3_). HRMS(+ESI) *m*/*z* calcd for C_19_H_18_ClN_2_O_2_ [M + H]^+^: 341.1057; found: 341.1056.

#### 3.4.10. 7-Chloro-6-((4-isopropylphenyl)amino)-2-methyl-5,8-quinolinequinone (**AQQ10**)

Obtained from the mixture (**QQ** and CeCl_3_·7H_2_O) and 4-isopropylaniline (0.153 g, 1.13 mmol) according to the general procedure, the title compound (**AQQ10**) was purified by column chromatography as a dark red oil. Yield: 71%. Purity: 100% (Retention time: 3.61 min). FTIR (ATR) υ (cm^−1^): 3321 (NH), 3048 (CH_aromatic_), 2959, 2870 (CH_aliphatic_), 1674 (>C=O), 1651, 1571, 1518, 1420, 1304, 1283, 1223, 1198, 1139, 1099, 1034. ^1^H NMR (500 MHz, CDCl_3_) *δ* (ppm): 8.35–8.20 (m, 1H, CH_aromatic_), 7.55–7.41 (m, 2H, NH and CH_aromatic_), 7.35–7.22 (m, 2H, CH_aromatic_), 7.17 (s, 1H, CH_aromatic_), 7.02 (d, *J* = 6.6 Hz, 1H, CH_aromatic_), 3.26–3.12 (m, 1H, CH(CH_3_)_2_), 2.77 (s, 3H, CH_3_), 1.25 (s, 6H, CH_3_). ^13^C NMR (125 MHz, CDCl_3_) *δ* (ppm): 180.1, 176.1 (>C=O), 166.0, 148.0, 146.9, 141.0, 134.9, 134.8, 126.7, 126.4, 124.7, 124.4, 115.1 (C_aromatic_ and C_q_), 33.6 (CH), 25.4, 24.0 (CH_3_). HRMS(+ESI) *m*/*z* calcd for C_19_H_17_ClN_2_O_2_ [M]^+^: 340.0979; found: 340.0981.

#### 3.4.11. 7-Chloro-6-((4-(diethylamino)phenyl)amino)-2-methyl-5,8-quinolinequinone (**AQQ11**)

Obtained from the mixture (**QQ** and CeCl_3_·7H_2_O) and *N,N*-diethyl-*p*-phenylenediamine (0.186 g, 1.13 mmol) according to the general procedure, the title compound (**AQQ11**) was purified by column chromatography as a dark red oil. Yield: 68%. Purity: 95% (Retention time: 3.66 min). FTIR (ATR) υ (cm^−1^): 3317 (NH), 3074 (CH_aromatic_), 2972, 2930 (CH_aliphatic_), 1667 (>C=O), 1608, 1574, 1520, 1505, 1450, 1355, 1306, 1262, 1223, 1143, 1038. ^1^H NMR (500 MHz, CDCl_3_) *δ* (ppm): 8.27 (d, *J* = 7.9 Hz, 1H, CH_aromatic_), 7.68 (br s, 1H, NH), 7.45 (d, *J* = 7.9 Hz, 1H, CH_aromatic_), 7.04–6.89 (m, 2H, CH_aromatic_), 6.61 (s, 2H, CH_aromatic_), 3.47–3.27 (m, 4H, NCH_2_), 2.77 (s, 3H, CH_3_), 1.18 (t, *J* = 6.7 Hz, 6H, CH_3_). ^13^C NMR (125 MHz, CDCl_3_) *δ* (ppm): 180.2, 175.9 (>C=O), 165.9, 148.3, 146.3, 141.1, 134.8, 126.5, 126.2, 125.0, 124.6, 111.0 (C_aromatic_ and C_q_), 44.5 (NCH_2_), 25.4, 12.5 (CH_3_). HRMS(+ESI) *m*/*z* calcd for C_20_H_21_ClN_3_O_2_ [M + H]^+^: 370.1322; found: 370.1320.

#### 3.4.12. 7-Chloro-6-((2,3-dimethylphenyl)amino)-2-methyl-5,8-quinolinequinone (**AQQ12**)

Obtained from the mixture (**QQ** and CeCl_3_·7H_2_O) and 2,3-dimethylaniline (0.137 g, 1.13 mmol) according to the general procedure, the title compound (**AQQ12**) was purified by column chromatography as an orange solid. Yield: 67%, 195.8–197.2 °C. Purity: 97% (Retention time: 3.70 min). FTIR (ATR) υ (cm^−1^): 3328 (NH), 3089 (CH_aromatic_), 2974, 2915 (CH_aliphatic_), 1665 (>C=O), 1611, 1578, 1496, 1464, 1378, 1313, 1276, 1221, 1144, 1104, 1071. ^1^H NMR (500 MHz, CDCl_3_) *δ* (ppm): 8.25 (d, *J* = 7.9 Hz, 1H, CH_aromatic_), 7.57–7.39 (m, 2H, NH and CH_aromatic_), 7.16–6.98 (m, 2H, CH_aromatic_), 6.89 (d, *J* = 6.3 Hz, 1H, CH_aromatic_), 2.75 (s, 3H, CH_3_), 2.29 (s, 3H, CH_3_), 2.17 (s, 3H, CH_3_). ^13^C NMR (125 MHz, CDCl_3_) *δ* (ppm): 179.9, 176.0 (>C=O), 166.0, 148.0, 141.8, 137.6, 136.0, 134.8, 132.7, 128.6, 126.7, 125.1, 124.6, 124.3, 114.1 (C_aromatic_ and C_q_), 25.4, 20.4, 14.4 (CH_3_). HRMS(+ESI) *m*/*z* calcd for C_18_H_15_ClN_2_O_2_ [M]^+^: 326.0822; found: 326.0827.

#### 3.4.13. 7-Chloro-6-((2,4-dimethylphenyl)amino)-2-methyl-5,8-quinolinequinone (**AQQ13**)

Obtained from the mixture (**QQ** and CeCl_3_·7H_2_O) and 2,4-dimethylaniline (0.137 g, 1.13 mmol) according to the general procedure, the title compound (**AQQ13**) was purified by column chromatography as an orange solid. Yield: 50%, 185.5–187.4 °C. Purity: 98% (Retention time: 3.70 min). FTIR (ATR) υ (cm^−1^): 3328 (NH), 3085 (CH_aromatic_), 2918, 2848 (CH_aliphatic_), 1663 (>C=O), 1607, 1583, 1506, 1481, 1376, 1313, 1285, 1218, 1202, 1144, 1125, 1036. ^1^H NMR (500 MHz, CDCl_3_) *δ* (ppm): 8.23 (d, *J* = 7.9 Hz, 1H, CH_aromatic_), 7.49–7.38 (m, 2H, NH and CH_aromatic_), 6.99 (s, 1H, CH_aromatic_), 6.92 (dd, *J* = 21.8, 7.9 Hz, 2H, CH_aromatic_), 2.73 (s, 3H, CH_3_), 2.29 (s, 3H, CH_3_), 2.20 (s, 3H, CH_3_). ^13^C NMR (125 MHz, CDCl_3_) *δ* (ppm): 179.9, 175.9 (>C=O), 165.9, 148.0, 141.7, 136.9, 134.8, 133.7, 133.5, 131.0, 126.7, 126.5, 126.3, 124.6, 113.9 (C_aromatic_ and C_q_), 25.4, 21.0, 18.1 (CH_3_). HRMS(+ESI) *m*/*z* calcd for C_18_H_15_ClN_2_O_2_ [M]^+^: 326.0822; found: 326.0829.

#### 3.4.14. 7-Chloro-6-((2,5-dimethylphenyl)amino)-2-methyl-5,8-quinolinequinone (**AQQ14**)

Obtained from the mixture (**QQ** and CeCl_3_·7H_2_O) and 2,5-dimethylaniline (0.137 g, 1.13 mmol) according to the general procedure, the title compound (**AQQ14**) was purified by column chromatography as an orange solid. Yield: 39%, 160.1–161.7 °C. Purity: 100% (Retention time: 3.76 min). FTIR (ATR) υ (cm^−1^): 3286 (NH), 3081 (CH_aromatic_), 2919 (CH_aliphatic_), 1679 (>C=O), 1573, 1518, 1483, 1374, 1314, 1285, 1219, 1147, 1121, 1036. ^1^H NMR (500 MHz, CDCl_3_) *δ* (ppm): 8.26 (d, *J* = 7.9 Hz, 1H, CH_aromatic_), 7.51–7.38 (m, 2H, NH and CH_aromatic_), 7.07 (d, *J* = 7.3 Hz, 1H, CH_aromatic_), 6.98 (d, *J* = 7.2 Hz, 1H, CH_aromatic_), 6.83 (s, 1H, CH_aromatic_), 2.76 (s, 3H, CH_3_), 2.28 (s, 3H, CH_3_), 2.21 (s, 3H, CH_3_). ^13^C NMR (125 MHz, CDCl_3_) *δ* (ppm): 179.9, 176.0 (>C=O), 166.0, 148.1, 141.5, 135.8, 135.6, 134.8, 130.6, 130.1, 127.7, 126.7, 126.7, 124.6, 114.3 (C_aromatic_ and C_q_), 25.4, 20.9, 17.7 (CH_3_). HRMS(+ESI) *m*/*z* calcd for C_18_H_15_ClN_2_O_2_ [M]^+^: 326.0822; found: 326.0826.

#### 3.4.15. 7-Chloro-6-((3,4-dimethylphenyl)amino)-2-methyl-5,8-quinolinequinone (**AQQ15**)

Obtained from the mixture (**QQ** and CeCl_3_·7H_2_O) and 3,4-dimethylaniline (0.137 g, 1.13 mmol) according to the general procedure, the title compound (**AQQ15**) was purified by column chromatography as a purple solid. Yield: 68%, 224–225 °C. Purity: 100% (Retention time: 3.78 min). FTIR (ATR) υ (cm^−1^): 3182 (NH), 3015 (CH_aromatic_), 2918 (CH_aliphatic_), 1678 (>C=O), 1641, 1570, 1556, 1518, 1501, 1319, 1293, 1227, 1141, 1042. ^1^H NMR (500 MHz, CDCl_3_) *δ* (ppm): 8.29 (d, *J* = 8.0 Hz, 1H, CH_aromatic_), 7.64 (br s, 1H, NH), 7.48 (d, *J* = 7.9 Hz, 1H, CH_aromatic_), 7.11 (d, *J* = 7.9 Hz, 1H, CH_aromatic_), 6.89 (s, 1H, CH_aromatic_), 6.84 (d, *J* = 5.9 Hz, 1H, CH_aromatic_), 2.79 (s, 3H, CH_3_), 2.28 (s, 3H, CH_3_), 2.27 (s, 3H, CH_3_). ^13^C NMR (125 MHz, CDCl_3_) *δ* (ppm): 180.1, 176.1 (>C=O), 166.0, 148.1, 140.9, 136.9, 134.9, 134.7, 134.6, 129.5, 126.7, 125.5, 124.7, 121.8, 115.0 (C_aromatic_ and C_q_), 25.5, 19.8, 19.4 (CH_3_). HRMS(+ESI) *m*/*z* calcd for C_18_H_16_ClN_2_O_2_ [M + H]^+^: 327.0900; found: 327.0900.

#### 3.4.16. 7-Chloro-6-((3,5-dimethylphenyl)amino)-2-methyl-5,8-quinolinequinone (**AQQ16**)

Obtained from the mixture (**QQ** and CeCl_3_·7H_2_O) and 3,5-dimethylaniline (0.137 g, 1.13 mmol) according to the general procedure, the title compound (**AQQ16**) was purified by column chromatography as a purple solid. Yield: 77%, 212–213 °C. Purity: 100% (Retention time: 3.83 min). FTIR (ATR) υ (cm^−1^): 3185 (NH), 3019 (CH_aromatic_), 2912, 2863 (CH_aliphatic_), 1676 (>C=O), 1645, 1570, 1555, 1511, 1464, 1370, 1305, 1288, 1225, 1158, 1142, 1101, 1047. ^1^H NMR (500 MHz, CDCl_3_) *δ* (ppm): 8.27 (d, *J* = 7.9 Hz, 1H, CH_aromatic_), 7.64 (br s, 1H, NH), 7.47 (d, *J* = 7.9 Hz, 1H, CH_aromatic_), 6.84 (s, 1H, CH_aromatic_), 6.69 (s, 2H, CH_aromatic_), 2.77 (s, 3H, CH_3_), 2.30 (s, 6H, CH_3_). ^13^C NMR (125 MHz, CDCl_3_) *δ* (ppm): 180.1, 176.1 (>C=O), 166.0, 148.0, 140.9, 138.1, 136.8, 134.9, 127.6, 126.7, 124.7, 122.0, 115.4 (C_aromatic_ and C_q_), 25.4, 21.3, 21.3 (CH_3_). HRMS(+ESI) *m*/*z* calcd for C_18_H_16_ClN_2_O_2_ [M + H]^+^: 327.0900; found: 327.0900.

### 3.5. Biological Evaluation

#### 3.5.1. MIC Determinations

MICs of the AQQs were determined by the broth microdilution technique that conformed with the Clinical and Laboratory Institute (CLSI) [81,82] against a panel of standard ATCC microorganisms. Serial two-fold dilutions ranging from 1250 to 0.6 mg/L were prepared in Mueller Hinton Broth for the tested bacteria and RPMI-1640 medium for the yeast, respectively. The stock solutions of molecules were also prepared in DMSO. Samples were allowed to incubate at 37 °C for 18–20 h. Each experiment was performed in duplicate.

According to the antimicrobial activity results, we aimed to identify in vitro activities of the **AQQ4** and **AQQ9** against clinically obtained strains by the broth microdilution dilution technique as described by the CLSI recommendations [81,82]. For this assay, 20 nonduplicates, nosocomially acquired *MRSA* isolated from blood specimens between April and September 2017 were obtained from the Department of Infectious Diseases and Clinical Microbiology, Faculty of Medicine, Istanbul Medipol University. All strains were identified using API STAPH (bioM’erieux). Then, all the tested *S. aureus* isolates were chosen by using oxacillin susceptibility to determine the methicillin-resistant isolates approved by CLSI (MIC ≥ 4 µg/mL) [81]. Moreover, for 24 clinically obtained *C. albicans* and *non-albicans* isolates from patients diagnosed with vulvovaginal candidiasis in January and December 2016 at the Clinical Microbiology Laboratories of Group Florence Nightingale Hospitals in Turkey. The yeasts were differentiated by CHROMagar and verified by API 20C AUX (BioMerieux, France) systems. 

#### 3.5.2. Determination of Time-Kill Curves

In order to observe the dynamic profile of the activity of tested molecules (**AQQ4** and **AQQ9**), the time-kill curve (TKC) method was performed at one and four times the MIC against one (1) *C. albicans* and one (1) *MRSA* clinical strains. Molecules-free controls were included for each strain. Inocula were quantified spectrophotometrically and added to the flasks to yield a final concentration of 1 × 10^6^ CFU/mL. The test tubes containing MHB for the *MRSA* strain and RPMI-1640 medium for the *C. albicans* isolate with and without (growth control) molecules in a final volume of 10 mL were incubated in a 37 °C calibrated shaking water bath, and viable counts were determined at 0, 2, 4, 6, and 24-h intervals after inoculation, by subculturing 0.1 mL serial dilutions onto TSA plates. All tests were performed in duplicate. The lower limit of detection for the time-kill assay was 1 log_10_ CFU/mL. Bactericidal activity was defined as a ≥3 log_10_ CFU/mL decrease from the initial inoculum.

#### 3.5.3. Biofilm Attachment and Inhibition of Biofilm Formation Assays

Biofilm attachment and inhibition of biofilm formation assays were performed as previously described method with some modifications [58]. For biofilm attachment, an overnight culture of strong, biofilm-producing clinically *MRSA* and *C. albicans* strains was diluted 1/50 to obtain 1 × 10^6^–1 × 10^7^ CFU/200 mL for bacteria in TSB supplement with 1% glucose for yeast in RPMI-1640 medium. Then, all strains were added to each well of 96-well tissue culture microtiter plates with 1/10× MIC of tested molecules. The plates were allowed to incubate for 1, 2, and 4 h for *MRSA* and 2, 4, and 6h for *C. albicans* at 37 °C. The positive control was studied strains in the using medium alone. After incubation, each well was washed with phosphate-buffered saline (PBS) solution three times and measured at OD 595 nm.

To inhibit biofilm formation, each of the tested strains was incubated in their medium and molecules at 1× and 1/10× in addition to 1/100× MIC in 37 °C for 24 h in microtiter plates. Six wells were used for each molecule. The positive control was the tested strains in their medium without molecules. After incubation, each well was washed with PBS solution three times and measured at OD 595 nm.

#### 3.5.4. Molecular Docking

All compounds were sketched using PerkinElmer ChemDraw version 21.0 and prepared using Gypsum-DL on Google Colab while applying Durrant lab filters (removes molecular variants, which though technically possible, were judged improbable). The proteins were prepared using UCSD Chimera version 1.4 [83] with all the default parameters under the Dock Prep module. The conversion of compounds and protein to .pdbqt format, grid preparation, and docking parameter file generation were performed using the scripts available under MGLTools 1.5.7. Grid preparation and molecular docking were performed using AutoDockTools version 4.2.6 [84] on a Fujitsu High-performance computing (HPC) Station (64 GB RAM, Intel^®^ Xeon^®^ CPU E5-2630 v3 @ 2.40GHz). The output files were processed using the scripts available under MGLTools 1.5.7, and data visualization was performed using BIOVIA Discovery Studio 2021. Further exploration of the protein–ligand interaction was performed using Protein–Ligand Interaction Profiler (PLIP) 2.2.0 tool [85].

#### 3.5.5. Statistical Analysis

All experiments were performed in two independent assays. Two-way ANOVA-Tukey’s multiple comparison test was used to compare differences between control and antimicrobials treated biofilms. *p*-Value < 0.0001 was considered statistically significant.

## 4. Conclusions

Two subseries of the AQQs (**AQQ1**–**16**) containing EWG or EDG in aryl amine were successfully synthesized by chlorooxidation of 8-hydroxy-2-methyl-quinoline (**HQ**) with sodium chlorate in concentrated HCl solution followed by amination with corresponding aryl amines in the presence of the Lewis acid as catalyst, respectively. We performed a detailed study for their in vitro antibacterial activity against both Gram-positive and Gram-negative bacteria and antifungal activity against fungi. These two subseries of the AQQs (**AQQ1**–**16**) exhibited excellent antimicrobial activity, particularly against *S. aureus*, *E. faecalis*, and *C. albicans,* with low micromolar MIC values compared to that of positive control drugs. The AQQs, **AQQ8**–**10** and **AQQ12**–**14** with an EDG in aryl amine displayed the strongest antibacterial activity against *S. aureus*. Moreover, **AQQ8**–**10** and **AQQ14** showed the best activity against *E. faecalis*. In contrast, AQQ4 with an EWG in the aryl amine exhibited obvious antifungal activity against *C. albicans*. Further studies were required to provide more insights into the antimicrobial action based on the MIC values. Thus, in vitro studies were performed with the selected QQs (**AQQ4** and **AQQ9**) by evaluating their antibiofilm activities against both *S. aureus* and *C. albicans* and time-kill kinetic study for better understanding. To the best of our knowledge, our study is the first record to evaluate the antibiofilm and bactericidal activities of **AQQ4** and **AQQ9** against clinically resistant species. In our study, **AQQ9** was found to be effective against the tested strains with the 39.06 mg/L MIC_90_. Moreover, in accordance with the TKC studies results, this molecule seems to be effective in a dose-dependent manner (over to 1 log_10_ reduction on viable cell count at 4× MIC) against *MRSA* isolates. This promising activity of **AQQ9** allows it to be considered as an option for further studies as a therapy against infections caused by clinically resistant Staphylococcus spp. While **AQQ4** displayed no inhibitory activity against clinically resistant *C. albicans* and *non-albicans* isolates, the results obtained from antibiofilm activity studies reveal that this molecule can be considered for future studies on the dispersal of *Candida* biofilms. During the discovery of new antibacterial and/or antifungal agents for infectious diseases, we mainly focus on the 1,4-quinone moiety. A lot of bioactive agents based on quinone chemistry like naphthoquinone, dimethylbenzoquinone, and quinolinequinone were prepared and investigated for their biological evaluation. Recent publications have further emphasized the importance of recent study for new antibiotics to be developed. The quinolinequinones studied in this research possess significant antibacterial and antifungal potentials. In conclusion, the antibacterial and antifungal activity of the two subseries of the AQQs (**AQQ1**–**16**) affords them great potential to be developed as novel antibacterial and antifungal drugs.

## Figures and Tables

**Table 1:** Crystallographic data for the **AQQ7** and **AQQ10**.

Identification Code	AQQ7	AQQ10
Chemical formula	C_17_H_13_ClN_2_O_2_	C_19_H_17_ClN_2_O_2_
Formula weight (g mol^−1^)	312.74	340.79
Temperature (K)	292(2)	299(2)
Radiation λ (Å)	0.71073	0.71073
Crystal system	Monoclinic	Trigonal
Space groups, Z	P 1 21/c 1, 4	R 3 c, 18
Unit cell dimensions (Å)	a = 4.8838 (5)	a = 28.573 (6)
	b = 25.878 (3)	b = 28.573 (6)
	c = 11.6611 (12)	c = 10.771 (3)
	α, γ = 90°	α, β = 90°
	β = 98.331 (2)°	γ = 120°
Volume (Å^3^)	1458.2 (3)	7615.(4)
Crystal sizes (mm)	0.068 × 0.146 × 0.500	0.038 × 0.070 × 0.434
dcalc (g cm^−3^)	1.425	1.338
Absorption coefficient (mm^−1^)	0.270	0.239
Tmin, Tmax	0.8770, 0.9820	0.9030, 0.9910
θ_max_, deg	27.48	25.02
Goodness-of-fit on F^2^	1.014	1.041
Index ranges	−6 ≤ h ≤ 6	−33 ≤ h ≤ 34
	−33 ≤ k ≤ 33	−33 ≤ k ≤ 34
	−15 ≤ l ≤ 15	−12 ≤ l ≤ 12
Reflections collected	21278	54427
Independent reflections	3346 [R(int) = 0.0599]	2980 [R(int) = 0.0831]
Final R indices [I > 2σ(I)]	2635 data	1930 data
	R_1_ = 0.0446	R_1_ = 0.0526
	wR_2_ = 0.1219	wR_2_ = 0.1344
R indices (all data)	R_1_ = 0.0579	R_1_ = 0.0919
	wR_2_ = 0.1305	wR_2_ = 0.1589
Refinement method	Full-matrix least-squares on F^2^	Full-matrix least-squares on F^2^
Data/restraints/parameters	3346/0/202	2980/1/220
Largest diff. peak and hole (eÅ^−3^)	0.446 and −0.277	0.189 and −0.144

**Table 2:** The minimal inhibitory concentration (MIC) values of the AQQs (**AQQ1**–**16**) for antibacterial activity ^a^.

**AQQs**		Subseries (X)	Substituent(s)	Gram-Negative Bacteria (MIC, μg/mL)				Gram-Positive Bacteria (MIC, μg/mL)		
General Formula	ID			*P. aeruginosa*	*E. coli*	*K. pneumoniae*	*P. mirabilis*	*S. aureus*	*S. epidermidis*	*E. faecalis*
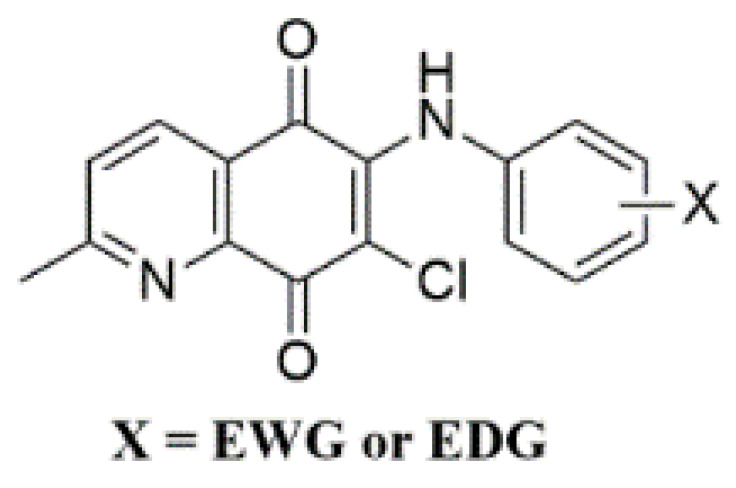	**AQQ1**	EWG	2-CF_3_	-	-	-	-	1250	1250	-
	**AQQ2**		3-CF_3_	-	-	-	-	625	78.12	312.50
	**AQQ3**		4-CF_3_	-	312.50	-	625	625	312.50	625
	**AQQ4**		4-CN	-	-	625	-	625	312.50	625
	**AQQ5**		3,5-diCF_3_	-	-	-	-	1250	39.06	-
	**AQQ6**	EDG	3-CH_3_	-	-	-	-	1250	78.12	625
	**AQQ7**		4-CH_3_	-	312.50	-	-	625	78.12	625
	**AQQ8**		2-CH(CH_3_)_2_	-	-	-	-	1.22	-	19.53
	**AQQ9**		3-CH(CH_3_)_2_	-	-	-	-	1.22	-	9.76
	**AQQ10**		4-CH(CH_3_)_2_	-	-	-	-	1.22	-	19.53
	**AQQ11**		4-N(CH_2_CH_3_)_2_	-	-	-	-	19.53	-	312.50
	**AQQ12**		2,3-diCH_3_	-	-	-	-	1.22	-	625
	**AQQ13**		2,4-diCH_3_	-	-	-	-	1.22	-	625
	**AQQ14**		2,5-diCH_3_	-	-	-	-	1.22	-	39.06
	**AQQ15**		3,4-diCH_3_	-	-	-	-	625	-	156.25
	**AQQ16**		3,5-diCH_3_	-	-	-	-	19.53	-	625
**Ciprofloxacin**				0.125	0.007	0.125	0.007	0.25	0.25	0.25

^a^ “-” means no activity.

**Table 3:** The minimal inhibitory concentration (MIC) values of the AQQs (**AQQ1**–**16**) for antifungal activity ^a^.

AQQs		Subseries (X)	Substituent(s)	Fungi (MIC, μg/mL)		
General Formula	ID			*C. albicans*	*C. parapsilosis*	*C. tropicalis*
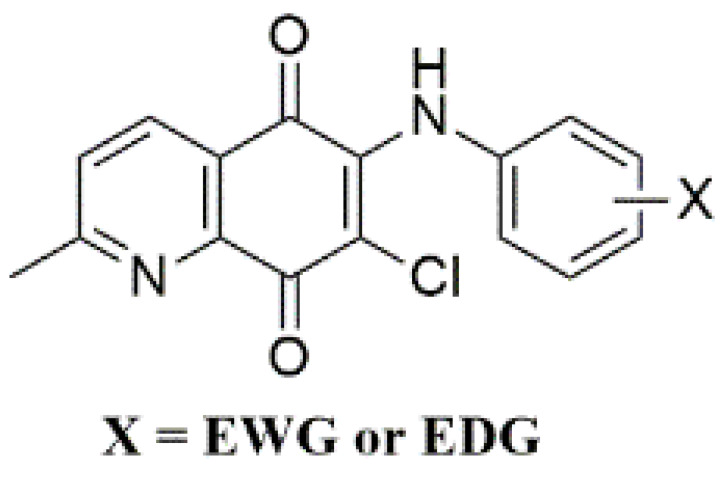	**AQQ1**	EWG	2-CF_3_	312.50	312.50	-
	**AQQ2**		3-CF_3_	312.50	312.50	-
	**AQQ3**		4-CF_3_	312.50	156.25	-
	**AQQ4**		4-CN	1.22	2.44	-
	**AQQ5**		3,5-diCF_3_	156.25	312.50	-
	**AQQ6**	EDG	3-CH_3_	156.25	78.12	-
	**AQQ7**		4-CH_3_	156.25	156.25	-
	**AQQ8**		2-CH(CH_3_)_2_	312.50	78.12	156.25
	**AQQ9**		3-CH(CH_3_)_2_	312.50	9.76	312.50
	**AQQ10**		4-CH(CH_3_)_2_	-	312.50	-
	**AQQ11**		4-N(CH_2_CH_3_)_2_	312.50	-	312.50
	**AQQ12**		2,3-diCH_3_	312.50	312.50	-
	**AQQ13**		2,4-diCH_3_	312.50	312.50	-
	**AQQ14**		2,5-diCH_3_	312.50	19.53	312.50
	**AQQ15**		3,4-diCH_3_	-	312.50	-
	**AQQ16**		3,5-diCH_3_	-	312.50	-
**Clotrimazole**				4.88	-	-
**Amphotericin B**				-	0.50	1.00

^a^ “-” means no activity.

**Table 4:** A summary of molecular docking studies for **AQQ4** and **AQQ9** against the three protein targets.

Protein	Compound	Binding Energy (Kcal/mol)	Inhibition Constant (Ki) (in µM)	No. of H-Bonds	Amino Acids Interactions
**3CW0**	**AQQ4**	−5.36	117.62	1 (LEU82)	11 (ARG204, VAL97, PRO85, ALA81, VAL77, PHE76, LEU98, SER96, GLU95, TRP87, VAL90)
	**AQQ9**	−5.66	70.88	1 (LEU82)	10 (VAL90, PRO85, PRO83, ARG204, LEU98, ALA81, TRP87, VAL77, TRP80, LEU82)
**3EAN**	**AQQ4**	−7.88	1.69	2 (GLU477, TRP407)	13 (HIS472, ILE478, PHE406, GLN494, GLY496, SER495, ASN418, GLU410, PRO408, VAL474, PRO473, LEU409, CYS475)
	**AQQ9**	−8.43	0.664	1 (TRP407)	14 (HIS472, PRO408, LEU409, GLU477, PRO473, CYS475, GLU410, VAL474, PHE406, CYS497, SER495, GLY496, GLN494, THR412)
**5XEX**	**AQQ4**	−8.06	1.24	2 (PRO101, PHE103)	11 (ILE513, LYS105, ASP55, LYS108, GLY106, TYR107, PHE57, PRO104, ARG100, LYS514, ILE513)
	**AQQ9**	−7.93	1.53	1 (PHE103)	12 (PRO104, TYR107, GLY106, ARG100, PHE57, LEU102, PRO101, GLN146, ASP516, ILE515, LYS514, LYS105)

## Data Availability

Data is contained within the article and Supplementary Material.

## Supplementary Materials

The following supporting information can be downloaded at: https://www.mdpi.com/article/10.3390/molecules27092923/s1, Table S1: Selected bond lengths (Å) with e.s.d in parantheses for the **AQQ7** and **AQQ10**; Table S2: Selected bond angles (°) with e.s.d in parantheses for the **AQQ7** and **AQQ10**; Table S3: Selected torsion angles (°) with e.s.d in parantheses for the **AQQ7** and **AQQ10**; Table S4: Hydrogen bond distances (Å) and angles (°) for the **AQQ7** and **AQQ10**; Figure S1: Purity chromatogram of the **AQQ1**; Figure S2: Purity chromatogram of the **AQQ2**; Figure S3: Purity chromatogram of the **AQQ3**; Figure S4: Purity chromatogram of the **AQQ4**; Figure S5: Purity chromatogram of the **AQQ5**; Figure S6: Purity chromatogram of the **AQQ6**; Figure S7: Purity chromatogram of the **AQQ7**; Figure S8: Purity chromatogram of the **AQQ8**; Figure S9: Purity chromatogram of the **AQQ9**; Figure S10: Purity chromatogram of the **AQQ10**; Figure S11: Purity chromatogram of the **AQQ11**; Figure S12: Purity chromatogram of the **AQQ12**; Figure S13: Purity chromatogram of the **AQQ13**; Figure S14: Purity chromatogram of the **AQQ14**; Figure S15: Purity chromatogram of the **AQQ15**; Figure S16: Purity chromatogram of the **AQQ16**; Figure S17: ^1^H NMR (500 MHz) spectrum of the **AQQ1** in CDCl_3_-*d*_1_; Figure S18: ^13^C NMR (125 MHz) spectrum of the **AQQ1** in CDCl_3_-*d*_1_; Figure S19: ^1^H NMR (500 MHz) spectrum of the **AQQ2** in CDCl_3_-*d*_1_; Figure S20: ^13^C NMR (125 MHz) spectrum of the **AQQ2** in CDCl_3_-*d*_1_; Figure S21: ^1^H NMR (500 MHz) spectrum of the **AQQ3** in DMSO-*d*_6_; Figure S22: ^13^C NMR (125 MHz) spectrum of the **AQQ3** in DMSO-*d*_6_; Figure S23: ^1^H NMR (500 MHz) spectrum of the **AQQ4** in CDCl_3_-*d*_1_; Figure S24: ^13^C NMR (125 MHz) spectrum of the **AQQ4** in CDCl_3_-*d*_1_; Figure S25: ^1^H NMR (500 MHz) spectrum of the **AQQ5** in CDCl_3_-*d*_1_; Figure S26: ^13^C NMR (125 MHz) spectrum of the **AQQ5** in CDCl_3_-*d*_1_; Figure S27: ^1^H NMR (500 MHz) spectrum of the **AQQ6** in CDCl_3_-*d*_1_; Figure S28: ^13^C NMR (125 MHz) spectrum of the **AQQ6** in CDCl_3_-*d*_1_; Figure S29: ^1^H NMR (500 MHz) spectrum of the **AQQ7** in CDCl_3_-*d*_1_; Figure S30: ^13^C NMR (125 MHz) spectrum of the **AQQ7** in CDCl_3_-*d*_1_; Figure S31: ^1^H NMR (500 MHz) spectrum of the **AQQ8** in CDCl_3_-*d*_1_; Figure S32: ^13^C NMR (125 MHz) spectrum of the **AQQ8** in CDCl_3_-*d*_1_; Figure S33: ^1^H NMR (500 MHz) spectrum of the **AQQ9** in CDCl_3_-*d*_1_; Figure S34: ^13^C NMR (125 MHz) spectrum of the **AQQ9** in CDCl_3_-*d*_1_; Figure S35: ^1^H NMR (500 MHz) spectrum of the **AQQ10** in CDCl_3_-*d*_1_; Figure S36: ^13^C NMR (125 MHz) spectrum of the **AQQ10** in CDCl_3_-*d*_1_; Figure S37: ^1^H NMR (500 MHz) spectrum of the **AQQ11** in CDCl_3_-*d*_1_; Figure S38: ^13^C NMR (125 MHz) spectrum of the **AQQ11** in CDCl_3_-*d*_1_; Figure S39: ^1^H NMR (500 MHz) spectrum of the **AQQ12** in CDCl_3_-*d*_1_; Figure S40: ^13^C NMR (125 MHz) spectrum of the **AQQ12** in CDCl_3_-*d*_1_; Figure S41: ^1^H NMR (500 MHz) spectrum of the **AQQ13** in CDCl_3_-*d*_1_; Figure S42: ^13^C NMR (125 MHz) spectrum of the **AQQ13** in CDCl_3_-*d*_1_; Figure S43: ^1^H NMR (500 MHz) spectrum of the **AQQ14** in CDCl_3_-*d*_1_; Figure S44: ^13^C NMR (125 MHz) spectrum of the **AQQ14** in CDCl_3_-*d*_1_; Figure S45: ^1^H NMR (500 MHz) spectrum of the **AQQ15** in CDCl_3_-*d*_1_; Figure S46: ^13^C NMR (125 MHz) spectrum of the **AQQ15** in CDCl_3_-*d*_1_; Figure S47: ^1^H NMR (500 MHz) spectrum of the **AQQ16** in CDCl_3_-*d*_1_; Figure S48: ^13^C NMR (125 MHz) spectrum of the **AQQ16** in CDCl_3_-*d*_1_.

## Abbreviations

ATCC	American Type Culture Collection
CCDC	Cambridge Crystallographic Data Center
CLSI	Clinical and Laboratory Institute
EDG	Electron-donating group
EWG	Electron-withdrawing group
HPC	High-performance computing
HPLC	High-performance liquid chromatography
MIC	Minimum inhibitory concentration
NMR	Nuclear magnetic resonance
PBS	Phosphate-buffered saline
SAR	Structure-activity relationship
TKC	Time-kill curve
TKS	Time-kill kinetic studies
TLC	Thin-layer chromatography

## References

[1] Boudreau M.A., Ding D., Meisel J.E., Janardhanan J., Spink E., Peng Z., Qian Y., Yamaguchi T., Testero S.A., O’Daniel P.I. (2020). Structure–Activity Relationship for the Oxadiazole Class of Antibacterials. ACS Med. Chem. Lett..

[2] Boucher H.W., Talbot G.H., Bradley J.S., Edwards J.E., Gilbert D., Rice L.B., Scheld M., Spellberg B., Bartlett J. (2009). Bad Bugs, No Drugs: No ESKAPE! An Update from the Infectious Diseases Society of America. Clin. Infect. Dis..

[3] Rossolini G.M., Arena F., Pecile P., Pollini S. (2014). Update on the antibiotic resistance crisis. Curr. Opin. Pharmacol..

[4] Liu N., Tu J., Dong G., Wang Y., Sheng C. (2018). Emerging New Targets for the Treatment of Resistant Fungal Infections. J. Med. Chem..

[5] Mayer F.L., Wilson D., Hube B. (2013). Candida albicans pathogenicity mechanisms. Virulence.

[6] Singh D.K., Tóth R., Gácser A. (2020). Mechanisms of Pathogenic Candida Species to Evade the Host Complement Attack. Front. Cell. Infect. Microbiol..

[7] Gullett N.P., Amin A.R., Bayraktar S., Pezzuto J.M., Shin D.M., Khuri F.R., Aggarwal B.B., Surh Y.-J., Kucuk O. (2011). Cancer prevention with natural compounds. Semin. Oncol..

[8] El-Dakhakhany M. (1963). Studies on the chemical constitution of Egyptian *Nigella sativa* L. seeds. Planta Med..

[9] Sugiura K. (1961). Antitumor activity of mitomycin C. Cancer Chemother. Rep..

[10] Hoyt M.T., Palchaudhuri R., Hergenrother P.J. (2010). Cribrostatin 6 induces death in cancer cells through a reactive oxygen species (ROS)-mediated mechanism. Investig. New Drugs.

[11] Shrestha J.P., Subedi Y.P., Chen L., Chang C.-W.T. (2015). A mode of action study of cationic anthraquinone analogs: A new class of highly potent anticancer agents. MedChemComm.

[12] Cummings J., Spanswick V.J., Tomasz M., Smyth J.F. (1998). Enzymology of mitomycin C metabolic activation in tumour tissue: Implications for enzyme-directed bioreductive drug development. Biochem. Pharmacol..

[13] Cummings J., Spanswick V.J., Ritchie A.A., Smyth J.F. (1998). Pharmacological determinants of the antitumour activity of mitomycin C—Implications for enzyme directed drug development. Ann. Oncol..

[14] Primeau A.J., Rendon A., Hedley D., Lilge L., Tannock I.F., Koido S., Hara E., Homma S., Torii A., Toyama Y. (2005). The Distribution of the Anticancer Drug Doxorubicin in Relation to Blood Vessels in Solid Tumors. Clin. Cancer Res..

[15] Patel O.P., Beteck R.M., Legoabe L.J. (2021). Antimalarial application of quinones: A recent update. Eur. J. Med. Chem..

[16] Di Marco N.I., Páez P.L., Lucero-Estrada C.S.M., Pungitore C.R. (2021). Naphthoquinones inhibit formation and viability of Yersinia enterocolitica biofilm. World J. Microbiol. Biotechnol..

[17] Silakari P., Silakari O., Piplani P. (2021). Systematic in silico Design, Synthesis, and Biological Studies of Some Novel 1,4-Benzoquinone Derivatives for the Prospective Management of Cognitive Decline. ACS Chem. Neurosci..

[18] Egu S.A., Ibezim A., Onoabedje E.A., Okoro U.C. (2017). Biological and in silico Evaluation of Quinolinedione and Naphthoquinone Derivatives as Potent Antibacterial Agents. ChemistrySelect.

[19] Keyari C.M., Kearns A.K., Duncan N.S., Eickholt E.A., Abbott G., Beall H.D., Diaz P. (2013). Synthesis of New Quinolinequinone Derivatives and Preliminary Exploration of their Cytotoxic Properties. J. Med. Chem..

[20] Vázquez K., Espinosa-Bustos C., Soto-Delgado J., Tapia R.A., Varela J., Birriel E., Segura R., Pizarro J., Cerecetto H., González M. (2015). New aryloxy-quinone derivatives as potential anti-Chagasic agents: Synthesis, trypanosomicidal activity, electrochemical properties, pharmacophore elucidation and 3D-QSAR analysis. RSC Adv..

[21] Ryu C.-K., Shim J.-Y., Yi Y.-J., Choi I.H., Chae M.J., Han J.-Y., Jung O.-J. (2004). Synthesis and antifungal activity of 5,8-quinazolinedione derivatives modified at positions 6 and 7. Arch. Pharmacal Res..

[22] Alfadhli A., Mack A., Harper L., Berk S., Ritchie C., Barklis E. (2016). Analysis of quinolinequinone reactivity, cytotoxicity, and anti-HIV-1 properties. Bioorg. Med. Chem..

[23] Johnson-Ajinwo O.R., Ullah I., Mbye H., Richardson A., Horrocks P., Li W.-W. (2018). The synthesis and evaluation of thymoquinone analogues as anti-ovarian cancer and antimalarial agents. Bioorg. Med. Chem. Lett..

[24] Pingaew R., Prachayasittikul V., Worachartcheewan A., Nantasenamat C., Prachayasittikul S., Ruchirawat S., Prachayasittikul V. (2015). Novel 1,4-naphthoquinone-based sulfonamides: Synthesis, QSAR, anticancer and antimalarial studies. Eur. J. Med. Chem..

[25] Santoso K.T., Menorca A., Cheung C.Y., Cook G.M., Stocker B.L., Timmer M.S.M. (2019). The synthesis and evaluation of quinolin-equinones as anti-mycobacterial agents. Bioorg. Med. Chem..

[26] Dey D., Ray R., Hazra B. (2014). Antitubercular and Antibacterial Activity of Quinonoid Natural Products Against Multi-Drug Resistant Clinical Isolates. Phytother. Res..

[27] Nain-Perez A., Barbosa L.C., Rodríguez-Hernández D., Mota Y.C., Silva T.F., Ramalho T.C., Modolo L.V. (2019). Antiureolytic Activity of Substituted 2,5-Diaminobenzoquinones. Chem. Biodivers..

[28] Fröhlich T., Reiter C., Saeed M.E.M., Hutterer C., Hahn F., Leidenberger M., Friedrich O., Kappes B., Marschall M., Efferth T. (2018). Synthesis of Thymoquinone–Artemisinin Hybrids: New Potent Antileukemia, Antiviral, and Antimalarial Agents. ACS Med. Chem. Lett..

[29] Ryu C.-K., Lee S.-Y., Kim N.Y., Hong J.A., Yoon J.H., Kim A. (2011). Synthesis and antifungal evaluation of 6-hydroxy-1H-carbazole-1,4(9H)-diones. Bioorg. Med. Chem. Lett..

[30] Ryu C.-K., Kim A., Im H.A., Kim J.Y. (2012). Synthesis and antifungal activity of 1-thia-4b-aza-cyclopenta[b]fluorene-4,10-diones. Bioorg. Med. Chem. Lett..

[31] Espinosa-Bustos C., Vázquez K., Varela J., Cerecetto H., Paulino M., Segura R., Pizarro J., Vera B., González M., Zarate A.M. (2020). New aryloxy-quinone derivatives with promising activity on *Trypanosoma cruzi*. Arch. Pharm..

[32] López-Lira C., Tapia R.A., Herrera A., Lapier M., Maya J.D., Soto-Delgado J., Oliver A.G., Lappin A.G., Uriarte E. (2021). New benzimidazolequinones as trypanosomicidal agents. Bioorg. Chem..

[33] Ko J.H., Yeon S.W., Ryu J.S., Kim T.-Y., Song E.-H., You H.-J., Park R.-E., Ryu C.-K. (2006). Synthesis and biological evaluation of 5-arylamino-6-chloro-1H-indazole-4,7-diones as inhibitors of protein kinase B/Akt. Bioorg. Med. Chem. Lett..

[34] Chung K.-H., Hong S.-Y., You H.-J., Park R.-E., Ryu C.-K. (2006). Synthesis and biological evaluation of 5-arylamino-1H-benzo[d]imidazole-4,7-diones as inhibitor of endothelial cell proliferation. Bioorg. Med. Chem..

[35] Ryu C.-K., Kang H.-Y., Lee S.K., Nam K.A., Hong C.Y., Ko W.-G., Lee B.-H. (2000). 5-Arylamino-2-methyl-4,7-dioxobenzothiazoles as inhibitors of cyclin-dependent kinase 4 and cytotoxic agents. Bioorg. Med. Chem. Lett..

[36] Ryu C.-K., Lee R.-Y., Kim N.Y., Kim Y.H., Song A.L. (2009). Synthesis and antifungal activity of benzo[d]oxazole-4,7-diones. Bioorg. Med. Chem. Lett..

[37] Hong S.-Y., Chung K.-H., You H.-J., Choi I.H., Chae M.J., Han J.-Y., Jung O.-J., Kang S.-J., Ryu C.-K. (2004). Synthesis and biological evaluation of benzimidazole-4,7-diones that inhibit vascular smooth muscle cell proliferation. Bioorg. Med. Chem. Lett..

[38] Valderrama J.A., Delgado V., Sepúlveda S., Benites J., Theoduloz C., Calderon P.B., Muccioli G.G. (2016). Synthesis and Cytotoxic Activity on Human Cancer Cells of Novel Isoquinolinequinone–Amino Acid Derivatives. Molecules.

[39] Ryu C.-K., Park R.-E., Ma M.-Y., Nho J.-H. (2007). Synthesis and antifungal activity of 6-arylamino-phthalazine-5,8-diones and 6,7-bis(arylthio)-phthalazine-5,8-diones. Bioorg. Med. Chem. Lett..

[40] Chung H.-J., Jung O.-J., Chae M.J., Hong S.-Y., Chung K.-H., Lee S.K., Ryu C.-K. (2005). Synthesis and biological evaluation of quinoxaline-5,8-diones that inhibit vascular smooth muscle cell proliferation. Bioorg. Med. Chem. Lett..

[41] Yoo H.W., Lee Y.S., Suh M.E., Kim D.J., Park S.W. (1998). Cytotoxic effects of quinoxaline derivatives on human cancer cell lines. Arch. Pharm..

[42] Kadela-Tomanek M., Bębenek E., Chrobak E., Boryczka S. (2019). 5,8-Quinolinedione Scaffold as a Promising Moiety of Bioactive Agents. Molecules.

[43] Ryu C.-K., Lee S.-K., Han J.-Y., Jung O.-J., Lee J.Y., Jeong S.H. (2005). Synthesis and antifungal activity of 5-arylamino-4,7-dioxobenzo[b]thiophenes. Bioorg. Med. Chem. Lett..

[44] Ryu C.K., Kang H.Y., Yi Y.J., Shin K.H., Lee B.H. (2000). Synthesis and antifungal activities of 5/6-arylamino-4,7-dioxobenzothiazoles. Bioorg. Med. Chem. Lett..

[45] Choi S.Y., Shin J.H., Ryu C.K., Nam K.Y., No K.T., Choo H.Y.P. (2006). The development of 3D-QSAR study and recursive par-titioning of heterocyclic quinone derivatives with antifungal activity. Bioorg. Med. Chem..

[46] Jordão A.K., Novais J., Leal B., Escobar A.C., dos Santos H.M., Castro H.C., Ferreira V. (2013). Synthesis using microwave irradiation and antibacterial evaluation of new N,O-acetals and N,S-acetals derived from 2-amino-1,4-naphthoquinones. Eur. J. Med. Chem..

[47] Lown J.W., Begleiter A., Johnson D., Morgan A.R. (1976). Studies related to antitumor antibiotics. Part V. Reactions of mitomycin C with DNA examined by ethidium fluorescence assay. Can. J. Biochem..

[48] Gould S.J., Weinreb S.M. (1982). Streptonigrin. Fortschr. Chem. Org. Nat./Prog. Chem. Org. Nat. Prod..

[49] Balitz D.M., Bush J.A., Bradner W.T., Doyle T.W., O’Herron F.A., Nettleton D.E. (1982). Isolation of lavendamycin. A new antibiotic from Streptomyces lavendulae. J. Antibiot..

[50] Defant A., Mancini I. (2019). Design, Synthesis and Cancer Cell Growth Inhibition Evaluation of New Aminoquinone Hybrid Mol-ecules. Molecules.

[51] Egu S.A., Okoro U.C., Onoabedje E.A. (2017). New Aryl Derivatives of Quinolinedione and Related Heterocyclic Compounds. J. Heterocycl. Chem..

[52] Patani G.A., LaVoie E.J. (1996). Bioisosterism: A Rational Approach in Drug Design. Chem. Rev..

[53] Meanwell N.A. (2011). Synopsis of Some Recent Tactical Application of Bioisosteres in Drug Design. J. Med. Chem..

[54] Ibis C., Tuyun A.F., Ozsoy-Gunes Z., Bahar H., Stasevych M.V., Musyanovych R.Y., Komarovska-Porokhnyavets O., Novikov V. (2011). Synthesis and biological evaluation of novel nitrogen- and sulfur-containing hetero-1,4-naphthoquinones as potent antifungal and antibacterial agents. Eur. J. Med. Chem..

[55] Tuyun A.F., Bayrak N.N., Yildirim H., Onul N., Kara E.M., Celik B.O. (2015). Synthesis and In Vitro Biological Evaluation of Aminonaphthoquinones and Benzo[b]phenazine-6,11-dione Derivatives as Potential Antibacterial and Antifungal Compounds. J. Chem..

[56] Yıldırım H., Bayrak N., Tuyun A.F., Kara E.M., Çelik B., Gupta G.K. (2017). 2,3-Disubstituted-1,4-naphthoquinones containing an arylamine with trifluoromethyl group: Synthesis, biological evaluation, and computational study. RSC Adv..

[57] Tuyun A.F., Yıldız M., Bayrak N., Yıldırım H., Kara E.M., Jannuzzi A.T., Celik B.O. (2019). Discovery of a new family of heterocyclic amine linked plastoquinone analogs for antimicrobial evaluation. Drug Dev. Res..

[58] Kara E.M., Bayrak N., Yildirim H., Yildiz M., Celik B.O., Tuyun A.F. (2020). Chlorinated plastoquinone analogs that inhibit Staphy-lococcus epidermidis and Candida albicans growth. Folia Microbiol..

[59] Mataracı-Kara E., Bayrak N., Yıldırım H., Yıldız M., Ataman M., Ozbek-Celik B., Tuyun A.F. (2021). Plastoquinone analogs: A po-tential antimicrobial lead structure intensely suppressing Staphylococcus epidermidis and Candida albicans growth. Med. Chem. Res..

[60] Yıldız M., Bayrak N., Yıldırım H., Mataracı-Kara E., Shilkar D., Jayaprakash V., Tuyun A.F. (2021). Exploration of brominated Plastoquinone analogs: Discovery and structure-activity relationships of small antimicrobial lead molecules. Bioorg. Chem..

[61] Bayrak N., Yıldız M., Yıldırım H., Kara E.M., Celik B.O., Tuyun A.F. (2020). Brominated plastoquinone analogs: Synthesis, structural characterization, and biological evaluation. J. Mol. Struct..

[62] Mataraci-Kara E., Bayrak N., Yildiz M., Yildirim H., Ozbek-Celik B., Tuyun A.F. (2020). Discovery and structure-activity relationships of the quinolinequinones: Promising antimicrobial agents and mode of action evaluation. Drug Dev. Res..

[63] Bayrak N., Yıldız M., Yıldırım H., Mataracı-Kara E., Tuyun A.F. (2021). Novel plastoquinone analogs containing benzocaine and its analogs: Structure-based design, synthesis, and structural characterization. Res. Chem. Intermed..

[64] Ramasamy S.K., Clemons W.M. (2009). Structure of the twin-arginine signal-binding protein DmsD fromEscherichia coli. Acta Crystallogr. Sect. F Struct. Biol. Cryst. Commun..

[65] Cheng Q., Sandalova T., Lindqvist Y., Arnér E. (2009). Crystal Structure and Catalysis of the Selenoprotein Thioredoxin Reductase 1. J. Biol. Chem..

[66] Wang X., Wang C., Wu M., Tian T., Cheng T., Zhang X., Zang J. (2017). Enolase binds to RnpA in competition with PNPase in Staphylococcus aureus. FEBS Lett..

[67] Shaikh I.A., Johnson F., Grollman A.P. (1987). Streptonigrin. 1. Structure-Activity Relationships Among Simple Bicyclic Analogues. Rate Dependence of DNA Degradation on Quinone Reduction Potential. J. Med. Chem..

[68] Yoon E.Y., Choi H.Y., Shin K.J., Yoo K.H., Chi D.Y., Kim D.J. (2000). The regioselectivity in the reaction of 6,7-dihaloquinoline-5,8-diones with amine nucleophiles in various solvents. Tetrahedron Lett..

[69] Kim Y.-S., Park S.-Y., Lee H.-J., Suh M.-E., Schollmeyer D., Lee C.-O. (2003). Synthesis and cytotoxicity of 6,11-Dihydro-pyrido- and 6,11-Dihydro-benzo [2,3-b]phenazine-6,11-dione derivatives. Bioorg. Med. Chem..

[70] Lee H.-J., Kim J.S., Park S.-Y., Suh M.-E., Kim H.J., Seo E.-K., Lee C.-O. (2004). Synthesis and cytotoxicity evaluation of 6,11-dihydro-pyridazo- and 6,11-dihydro-pyrido[2,3-b]phenazine-6,11-diones. Bioorg. Med. Chem..

[71] Ryu C.-K., Choi J.-A., Kim S.-H. (1998). Synthesis and antifungal evaluation of 6-(N-arylamino)-7-methylthio-5,8-quinolinediones. Arch. Pharmacal Res..

[72] Kadela-Tomanek M., Jastrzębska M., Bębenek E., Chrobak E., Latocha M., Kusz J., Tarnawska D., Boryczka S. (2017). New Acetylenic Amine Derivatives of 5,8-Quinolinediones: Synthesis, Crystal Structure and Antiproliferative Activity. Crystals.

[73] Kara E.M., Celik B.O. (2018). Investigation of the effects of various antibiotics against Klebsiella pneumoniae biofilms on in vitro catheter model. J. Chemother..

[74] Costerton J.W., Lewandowski Z., Caldwell D.E., Korber D.R., Lappin-Scott H.M. (1995). Microbial biofilms. Annu. Rev. Microbiol..

[75] Bruker (2014). APEX2, Version 2014.1-1.

[76] Bruker (2013). SAINT, Version 8.34A.

[77] Bruker (2012). SADABS, Version 2012/2.

[78] Bruker (2000). SHELXTL, Version 6.14.

[79] Spek A.L. (2009). Structure validation in chemical crystallography. Acta Crystallogr. Sect. D Biol. Crystallogr..

[80] Macrae C.F., Edgington P.R., McCabe P., Pidcock E., Shields G.P., Taylor R., Towler M., van de Streek J. (2006). Mercury: Visualization and analysis of crystal structures. J. Appl. Crystallogr..

[81] Clinical and Laboratory Standards Institute (2020). Clinical and Laboratory Standards Institute (CLSI), Performance Standards for Antimicrobial Susceptibility Testing.

[82] Clinical and Laboratory Standards Institute (1997). Clinical and Laboratory Standards Institute (CLSI), Reference Method for Broth Dilution Antifungal Susceptibility Testing of Yeasts.

[83] Pettersen E.F., Goddard T.D., Huang C.C., Couch G.S., Greenblatt D.M., Meng E.C., Ferrin T.E. (2004). UCSF Chimera—A visualization system for exploratory research and analysis. J. Comput. Chem..

[84] Morris G.M., Huey R., Lindstrom W., Sanner M.F., Belew R.K., Goodsell D.S., Olson A.J. (2009). AutoDock4 and AutoDockTools4: Automated docking with selective receptor flexibility. J. Comput. Chem..

[85] Salentin S., Schreiber S., Haupt V.J., Adasme M.F., Schroeder M. (2015). PLIP: Fully automated protein-ligand interaction profiler. Nucleic Acids Res..

