# Supplementary material for: Highly Active Small Aminated Quinolinequinones against Drug-Resistant Staphylococcus aureus and Candida albicans
Source: Molecules. 2022 May 3;27(9):2923. doi: 10.3390/molecules27092923 (PMC9104734; doi:10.3390/molecules27092923)
Supplement: Supplementary file 1 [file molecules-27-02923-s001.zip › molecules-1696305-supplementary.pdf]

Highly Active Small Aminated Quinolinequinones against Drug-Resistant *Staphylococcus aureus* and *Candida albicans*

Hatice Yıldırım [1], Nilüfer Bayrak [1], Mahmut Yıldız [2], Fatıma Nur Yılmaz [3], Emel Mataracı-Kara [3], Deepak Shilkar [4], Venkatesan Jayaprakash [4] and Amaç Fatih TuYuN *,[5]

[1] *Department of Chemistry, Engineering Faculty, Istanbul University-Cerrahpasa, Avcilar, Istanbul 34320, Turkey*

[2] *Department of Chemistry, Gebze Technical University, Gebze, Kocaeli 41400, Turkey*

[3] *Department of Pharmaceutical Microbiology, Pharmacy Faculty, Istanbul University, Beyazit, Istanbul 34116, Turkey*

[4] *Department of Pharmaceutical Sciences & Technology, Birla Institute of Technology, Mesra, Ranchi 835 215, Jharkhand, India*

[5] *Department of Chemistry, Faculty of Science, Istanbul University, Fatih, Istanbul 34116, Turkey*

* Author to whom correspondence should be addressed; E-Mail: aftuyun@gmail.com or aftuyun@istanbul.edu.tr; Tel.: +90-212-440-0000.

Contents

The details of all crystallographic data of the **AQQ7** and **AQQ10** S3–S4

Purity chromatograms of the aminated QQs (**AQQ1–16**) S5–S12

^{1}H and ^{13}C spectra of the aminated QQs (**AQQ1–16**) S13–S28

Table S1. Selected bond lengths (Å) with e.s.d in parantheses for the **AQQ7** and **AQQ10**.

**AQQ7**				**AQQ10**			
Cl1-C9	1.7304(18)	C5-C6	1.382(3)	Cl1-C8	1.726(6)	C17-C19	1.291(17)
O2-C10	1.225(2)	C16-C17	1.495(3)	N1-C10	1.321(9)	C19-H19A	0.96
N1-C5	1.420(2)	C17-H17A	0.96	C10-C5	1.379(9)	O1-C9	1.205(8)
N2-C11	1.329(2)	O2-C13	1.219(2)	O2-C6	1.221(8)	N1-C2	1.354(9)
C1-H1B	0.96	N2-C16	1.334(3)	N2-C11	1.432(9)	C8-C7	1.364(9)
C3-C4	1.380(3)	C12-C11	1.394(2)	C11-C16	1.354(10)	N2-C7	1.341(9)
C15-C16	1.395(3)	C8-C9	1.374(2)	C18-C17	1.408(18)	C4-C3	1.366(12)

Table S2. Selected bond angles (°) with e.s.d in parantheses for the **AQQ7** and **AQQ10**.

**AQQ7**				**AQQ10**			
H1A-C1-H1B	109.5	O2-C10-C9	122.89(17)	C10-N1-C2	118.3(7)	C19-C17-H17	91.0
C15-C14-C12	118.70(19)	C9-C10-C11	118.08(15)	C9-C8-Cl1	114.1(5)	C5-C10-C9	119.9(6)
C4-C5-C6	120.20(18)	C2-C7-C6	121.4(2)	C10-C5-C6	121.6(6)	C7-C8-Cl1	121.1(5)
N1-C8-C13	112.70(15)	C11-N2-C16	118.01(18)	O1-C9-C8	121.7(6)	C10-C5-C4	118.1(7)
C5-C6-C7	119.4(2)	C5-C4-C3	119.6(2)	C5-C6-C7	119.3(6)	C4-C5-C6	120.4(7)
O2-C13-C8	119.26(17)	C4-C5-N1	118.83(17)	C16-C11-C12	120.9(7)	O1-C9-C10	121.3(6)
N2-C16-C15	121.98(19)	C9-C8-C13	118.24(16)	C12-C11-N2	120.9(7)	C7-N2-C11	131.1(6)
C11-C12-C13	120.79(16)	O2-C13-C12	121.72(17)	C11-C16-C15	119.1(8)	C14-C15-C16	121.2(8)
C7-C2-C3	118.02(19)	C14-C12-C13	121.35(17)	N2-C7-C6	112.3(6)	N1-C2-C3	120.4(8)
C8-C9-Cl1	121.71(14)	O2-C10-C11	119.02(17)	H1B-C1-H1C	109.5	C8-C7-C6	117.2(6)
H17A-C17-17B	109.5	C12-C11-C10	119.71(16)	C17-C18-H18B	109.5	H18A-C18-H18C	109.5

Table S3. Selected torsion angles (°) with e.s.d in parantheses for the **AQQ7** and **AQQ10**.

AQQ7				AQQ10			
C2-C3-C4-C5	0.1(4)	C8-C9-C10-O2	176.43(17)	C2-N1-C10-C5	-3.0(9)	C12-C11-C16-C15	1.7(11)
N1-C8-C13-O2	-1.6(2)	C16-N2-C11-C12	0.6(3)	N1-C10-C5-C4	3.7(9)	C14-C15-C16-C11	0.1(12)
N1-C8-C13-C12	177.66(15)	O2-C10-C11-C12	176.46(17)	N1-C10-C5-C6	-176.3(6)	C6-C5-C4-C3	179.1(6)
C11-N2-C16-C15	-3.3(3)	C3-C4-C5-N1	-177.8(2)	C7-C8-C9-O1	177.0(7)	Cl1-C8-C7-N2	-3.9(10)
C14-C15-C16-N2	3.4(3)	C13-C8-C9-Cl1	-169.66(12)	C7-C8-C9-C10	-0.8(9)	Cl1-C8-C7-C6	170.8(5)
C15-C14-C12-C11	-1.6(3)	Cl1-C9-C10-O2	-5.6(2)	N1-C10-C9-O1	2.1(9)	C1-C2-C3-C4	-175.6(8)
O1-C13-C12-C11	177.16(18)	C16-N2-C11-C10	-178.64(16)	N1-C10-C9-C8	180.0(6)	C2-N1-C10-C9	179.9(6)
N1-C8-C9-C10	-174.65(17)	C1-C2-C7-C6	178.9(2)	C10-C5-C6-O2	174.4(6)	N2-C11-C16-C15	176.2(7)
C9-C10-C11-N2	176.99(15)	C9-C10-C11-N2	176.99(15)	C7-N2-C11-C16	144.6(7)	C16-C15-C14-C13	-1.8(12)
C13-C12-C11-C10	5.5(2)	C13-C12-C11-N2	-173.78(16)	C10-N1-C2-C3	-0.5(11)	O2-C6-C7-C8	-172.8(6)
C5-N1-C8-C9	21.7(3)	N1-C8-C9-Cl1	7.5(3)	Cl1-N2-C7-C8	-23.4(12)	Cl1-C8-C9-O1	1.7(9)
C8-N1-C5-C6	44.8(3)	O2-C10-C11-N2	-4.2(2)	C11-N2-C7-C6	161.7(6)	O2-C6-C7-N2	2.9(9)

Table S4. Hydrogen bond distances (Å) and angles (°) for the **AQQ7** and **AQQ10**.

AQQ7					AQQ10				
	**Donor-H**	**Acceptor-H**	**Donor-Acceptor**	**Angle**		**Donor-H**	**Acceptor-H**	**Donor-Acceptor**	**Angle**
N1-H1...O2	0.86	2.10	2.878(2)	150.9	C16-H16...O1	0.93	2.64	3.472(10)	148.6
N1-H1...Cl1	0.86	2.89	3.5412(16)	134.2					

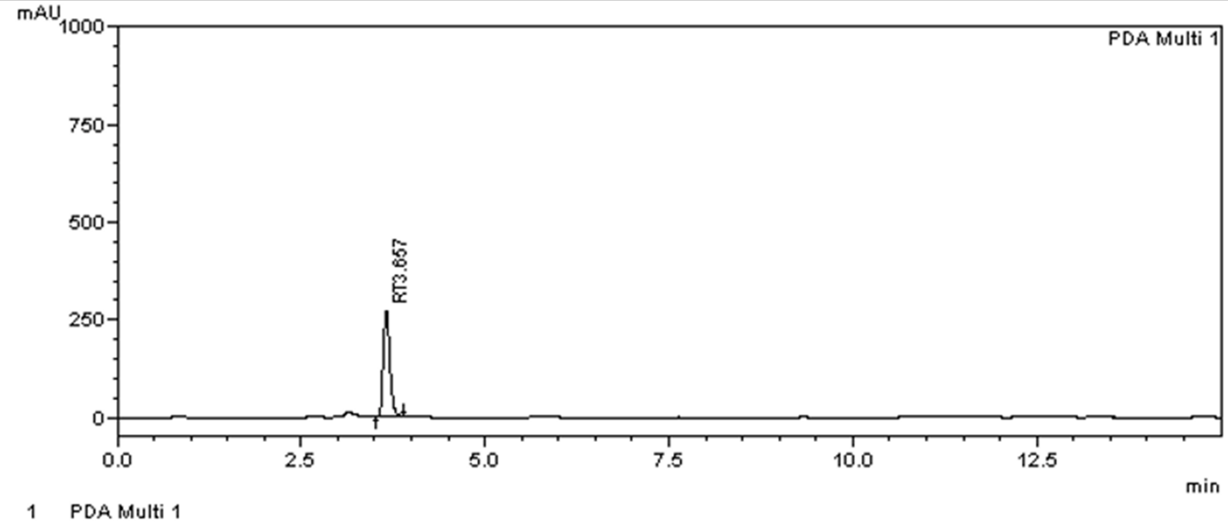

<Results>

PDA Ch1 215nm 4nm

Peak#	Ret. Time	Area	Height	Area %	Height %	Resolution
1	3.657	1708587	271221	100.000	100.000	0.000
Total		1708587	271221	100.000	100.000	

Figure S1. Purity chromatogram of the **AQQ1**.

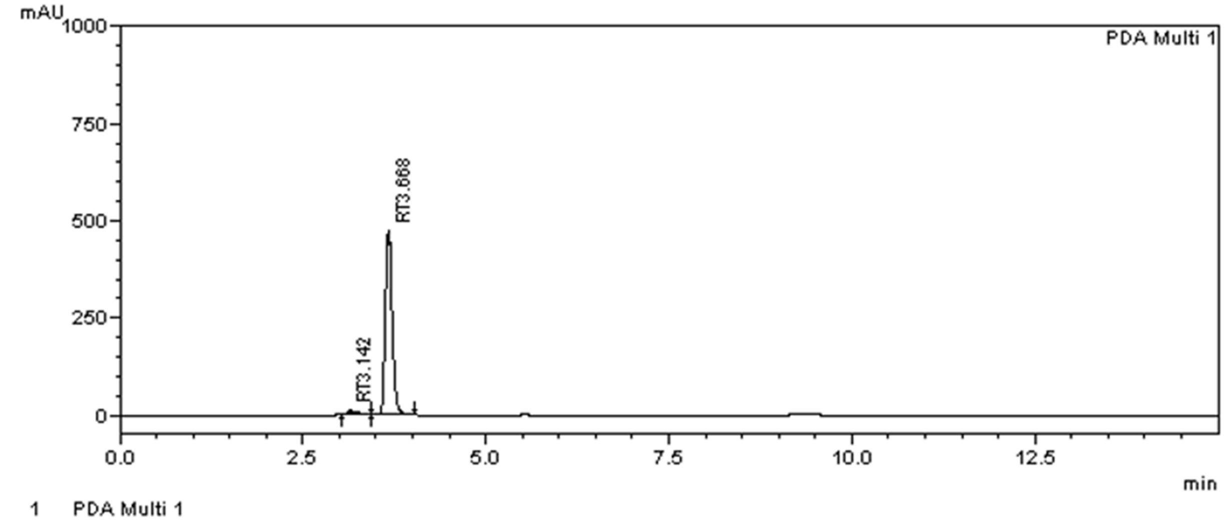

<Results>

PDA Ch1 215nm 4nm

Peak#	Ret. Time	Area	Height	Area %	Height %	Resolution
1	3.142	109217	10900	3.401	2.239	0.000
2	3.668	3101916	476029	96.599	97.761	2.506
Total		3211134	486929	100.000	100.000	

Figure S2. Purity chromatogram of the **AQQ2**.

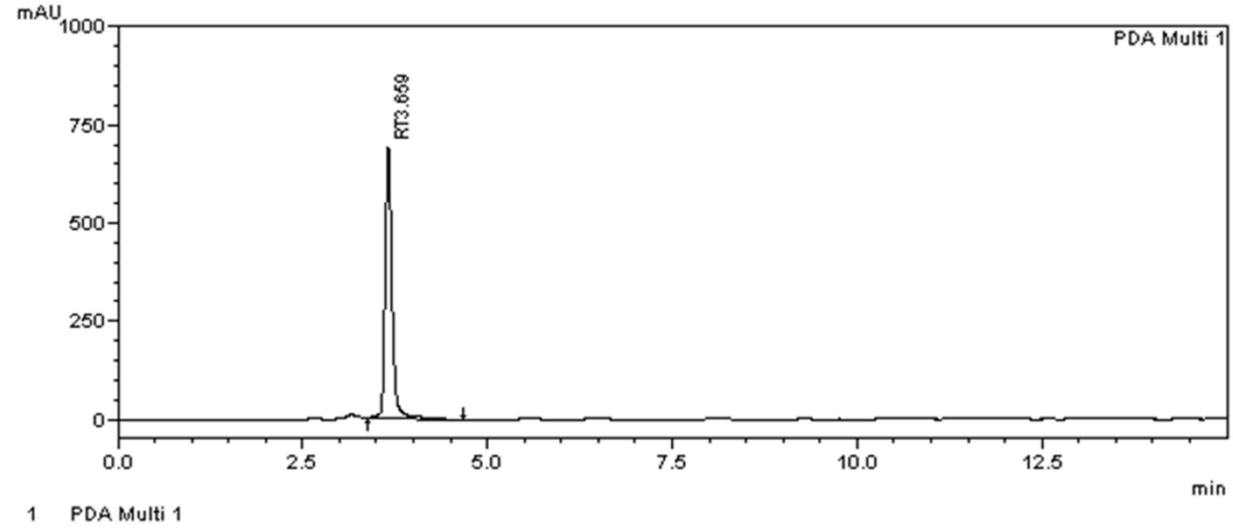

<Results>

PDA Ch1 215nm 4nm

Peak#	Ret. Time	Area	Height	Area %	Height %	Resolution
1	3.659	4498963	688023	100.000	100.000	0.000
Total		4498963	688023	100.000	100.000	

Figure S3. Purity chromatogram of the **AQQ3**.

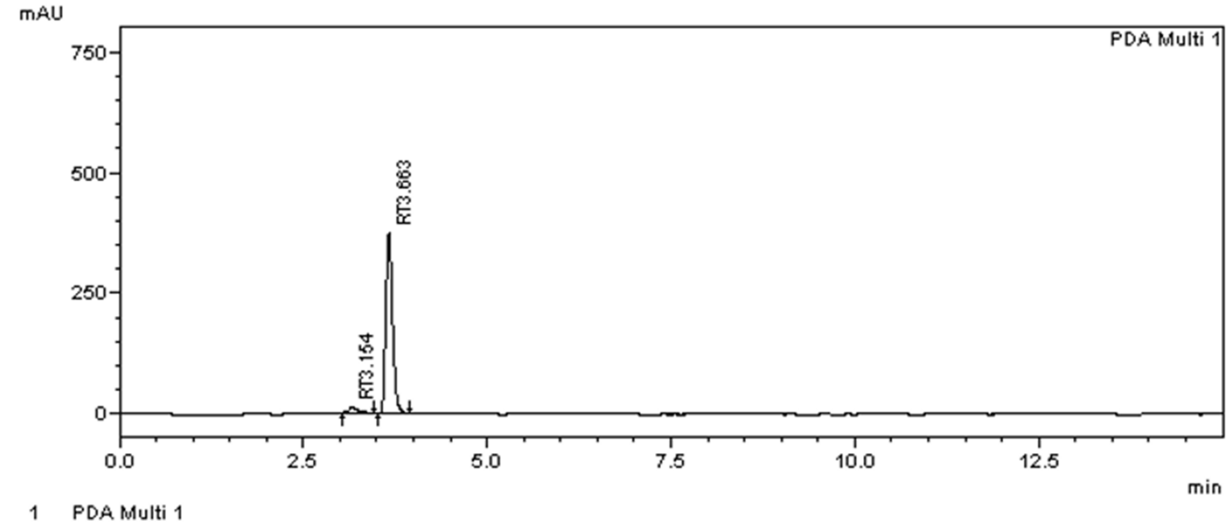

<Results>

PDA Ch1 215nm 4nm

Peak#	Ret. Time	Area	Height	Area %	Height %	Resolution
1	3.154	103425	10854	4.140	2.839	0.000
2	3.663	2394651	371446	95.860	97.161	2.532
Total		2498076	382299	100.000	100.000	

Figure S4. Purity chromatogram of the **AQQ4**.

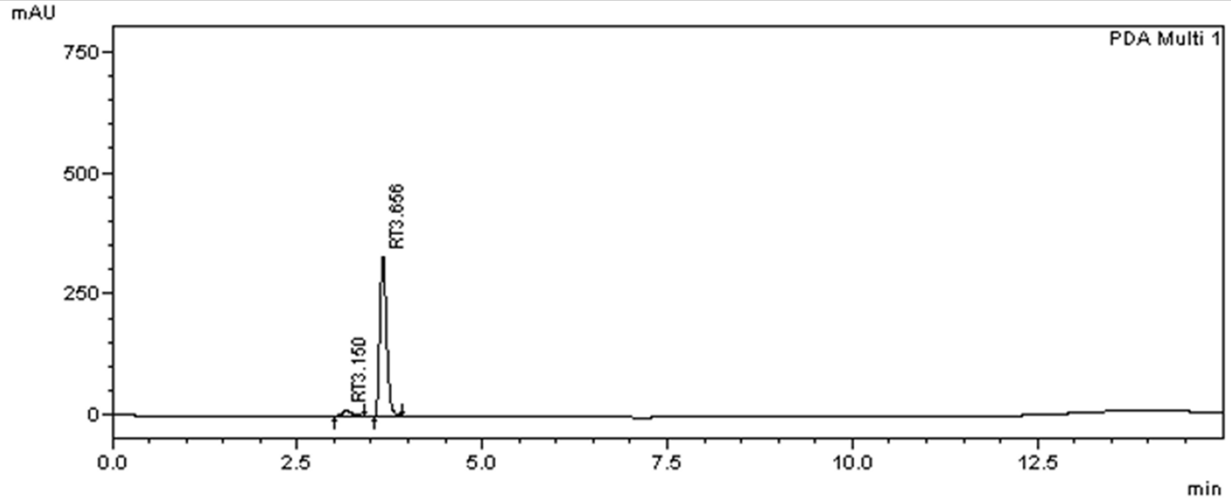

1 PDA Multi 1

<Results>

PDA Ch1 215nm 4nm

Peak#	Ret. Time	Area	Height	Area %	Height %	Resolution
1	3.150	101019	10482	4.615	3.099	0.000
2	3.656	2087673	327748	95.385	96.901	2.472
Total		2188692	338230	100.000	100.000	

Figure S5. Purity chromatogram of the **AQQ5**.

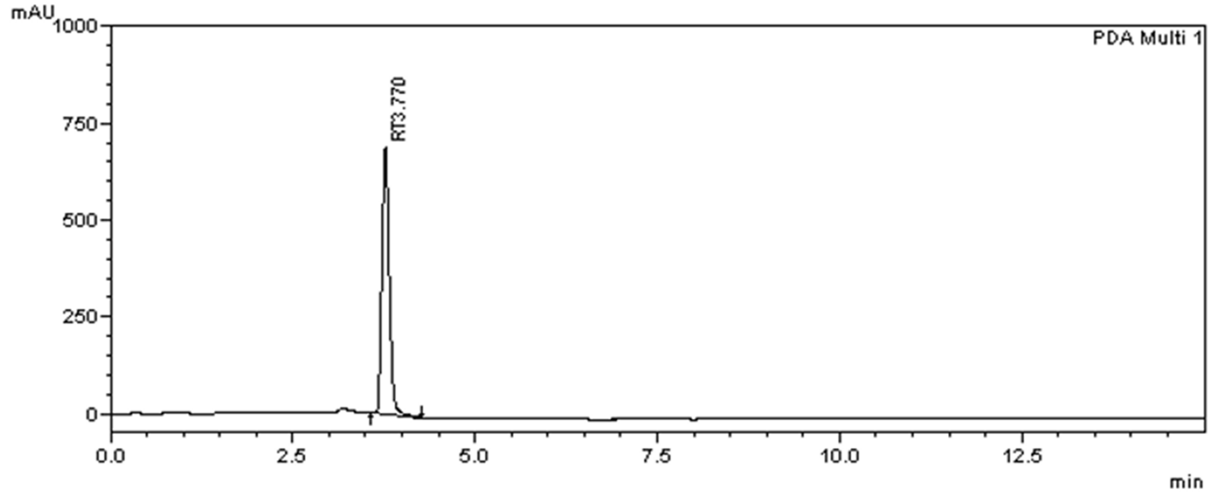

1 PDA Multi 1

<Results>

PDA Ch1 215nm 4nm

Peak#	Ret. Time	Area	Height	Area %	Height %	Resolution
1	3.770	4823931	686013	100.000	100.000	0.000
Total		4823931	686013	100.000	100.000	

Figure S6. Purity chromatogram of the **AQQ6**.

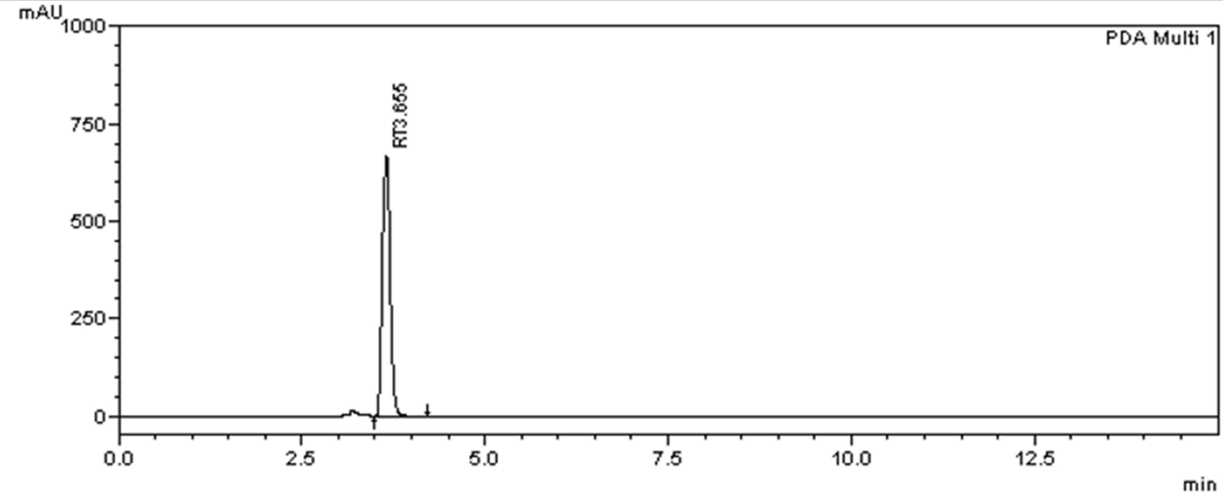

1 PDA Multi 1

<Results>

PDA Ch1 215nm 4nm

Peak#	Ret. Time	Area	Height	Area %	Height %	Resolution
1	3.655	5026476	665375	100.000	100.000	0.000
Total		5026476	665375	100.000	100.000	

Figure S7. Purity chromatogram of the **AQQ7**.

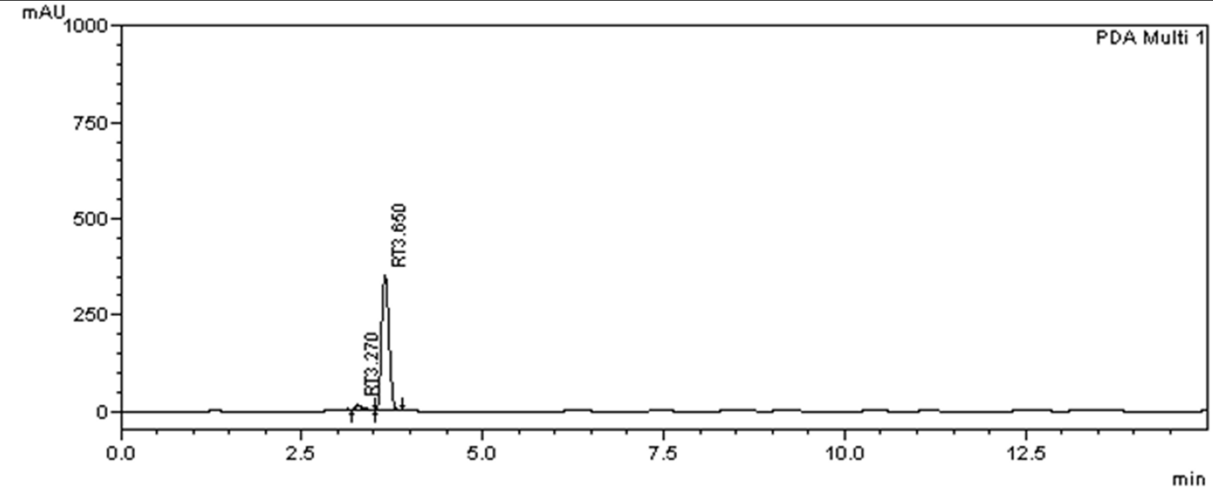

1 PDA Multi 1

<Results>

PDA Ch1 215nm 4nm

Peak#	Ret. Time	Area	Height	Area %	Height %	Resolution
1	3.270	119116	14086	4.587	3.864	0.000
2	3.650	2477654	350454	95.413	96.136	1.855
Total		2596770	364540	100.000	100.000	

Figure S8. Purity chromatogram of the **AQQ8**.

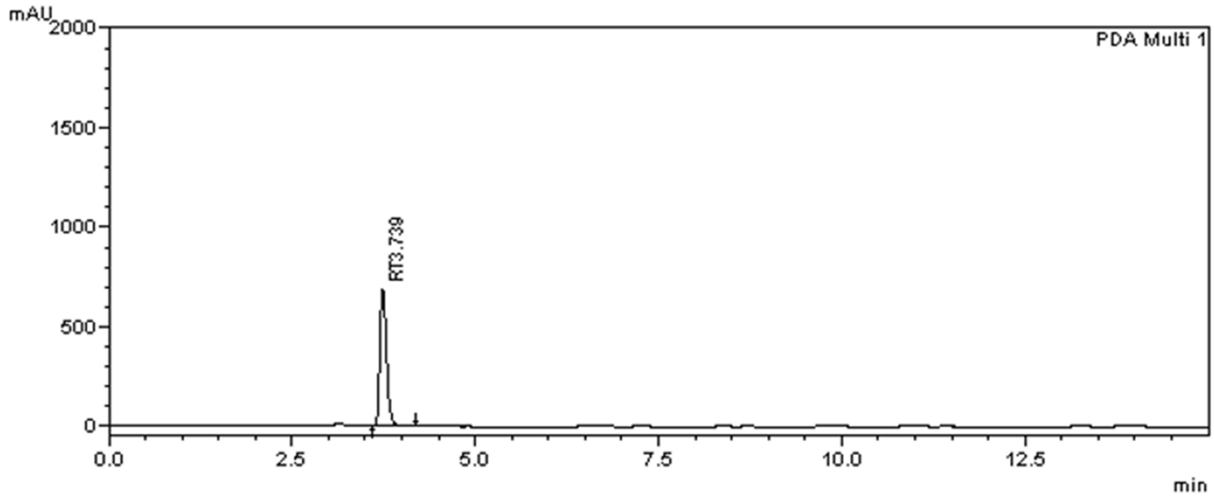

1 PDA Multi 1

<Results>

PDA Ch1 215nm 4nm

Peak#	Ret. Time	Area	Height	Area %	Height %	Resolution
1	3.739	4381385	683342	100.000	100.000	0.000
Total		4381385	683342	100.000	100.000	

Figure S9. Purity chromatogram of the **AQQ9**.

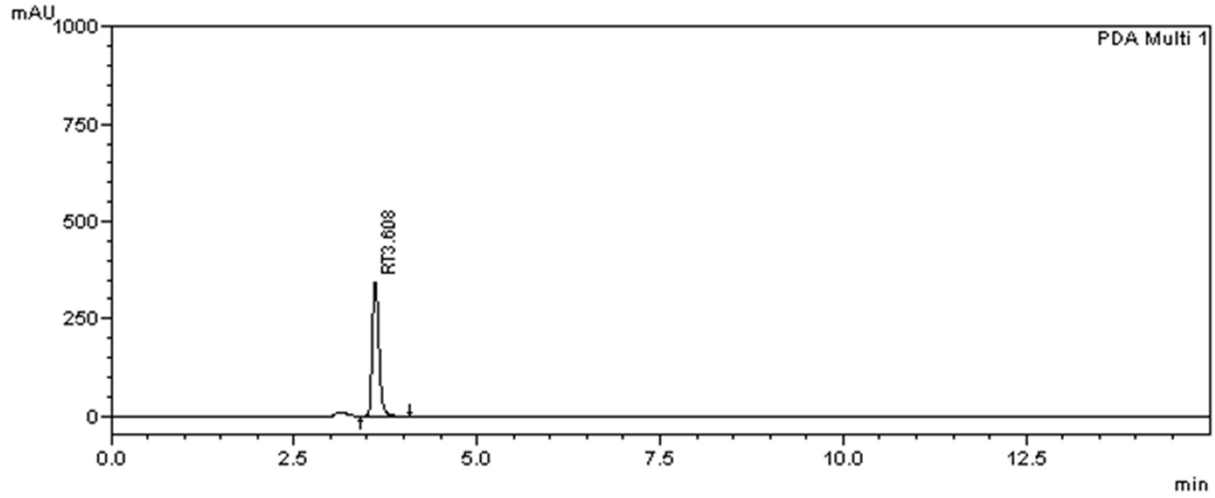

1 PDA Multi 1

<Results>

PDA Ch1 215nm 4nm

Peak#	Ret. Time	Area	Height	Area %	Height %	Resolution
1	3.608	2179427	343735	100.000	100.000	0.000
Total		2179427	343735	100.000	100.000	

Figure S10. Purity chromatogram of the **AQQ10**.

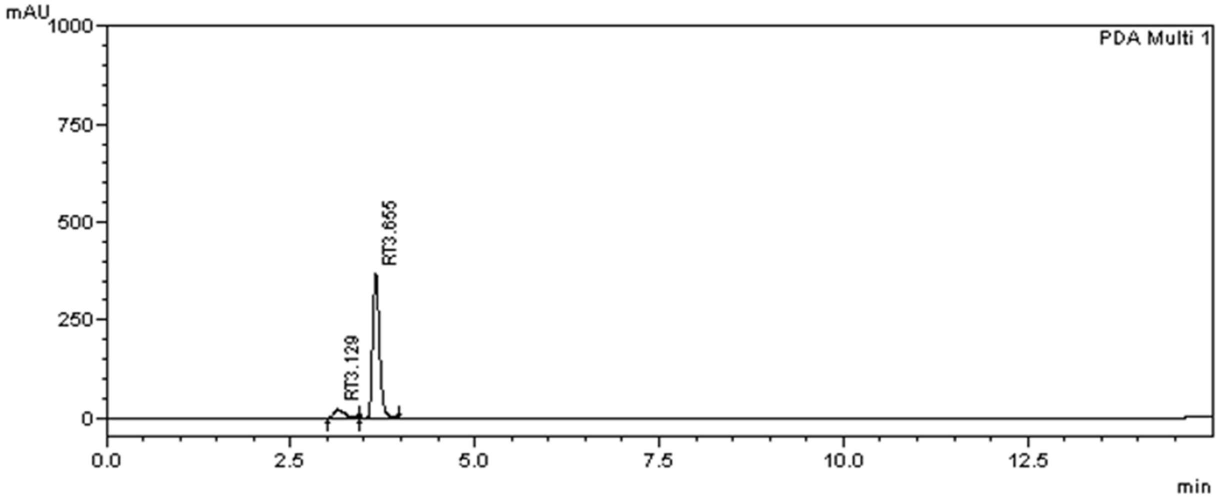

1 PDA Multi 1

<Results>

PDA Ch1 215nm 4nm

Peak#	Ret. Time	Area	Height	Area %	Height %	Resolution
1	3.129	196855	20450	7.743	5.264	0.000
2	3.655	2345427	368013	92.257	94.736	2.562
Total		2542282	388463	100.000	100.000	

Figure S11. Purity chromatogram of the **AQQ11**.

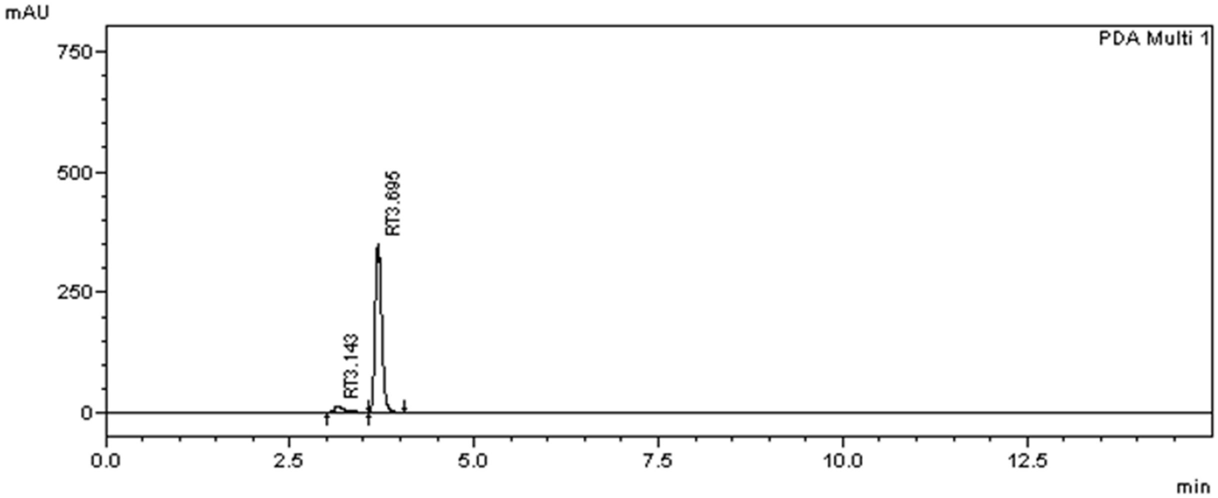

1 PDA Multi 1

<Results>

PDA Ch1 215nm 4nm

Peak#	Ret. Time	Area	Height	Area %	Height %	Resolution
1	3.143	111531	11282	4.861	3.159	0.000
2	3.695	2182918	345871	95.139	96.841	2.746
Total		2294449	357153	100.000	100.000	

Figure S12. Purity chromatogram of the **AQQ12**.

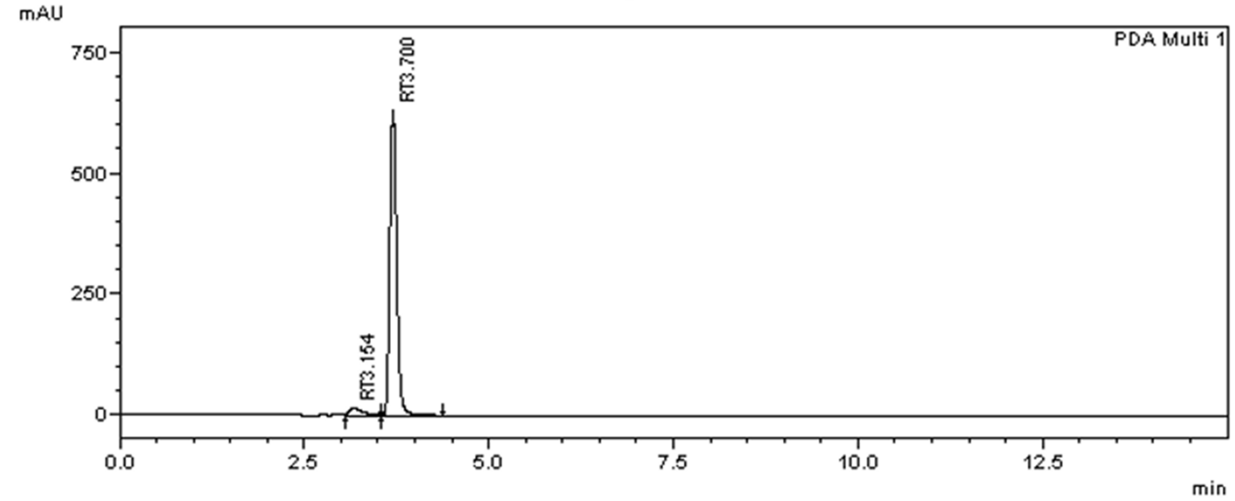

1 PDA Multi 1

<Results>

PDA Ch1 215nm 4nm

Peak#	Ret. Time	Area	Height	Area %	Height %	Resolution
1	3.154	190088	16142	4.395	2.500	0.000
2	3.700	4134779	629490	95.605	97.500	2.506
Total		4324867	645632	100.000	100.000	

Figure S13. Purity chromatogram of the **AQQ13**.

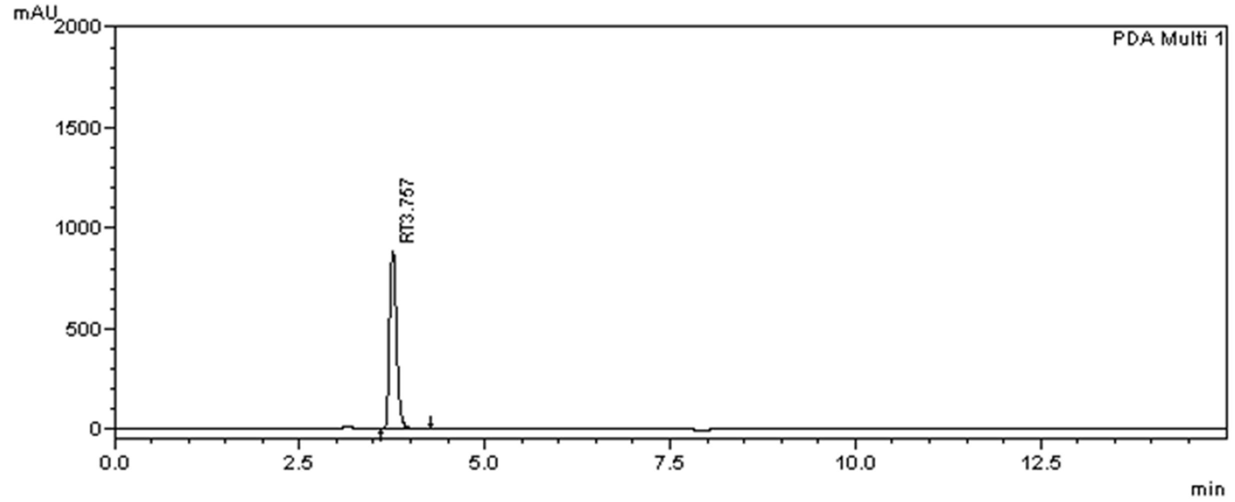

1 PDA Multi 1

<Results>

PDA Ch1 215nm 4nm

Peak#	Ret. Time	Area	Height	Area %	Height %	Resolution
1	3.757	5864654	885606	100.000	100.000	0.000
Total		5864654	885606	100.000	100.000	

Figure S14. Purity chromatogram of the **AQQ14**.

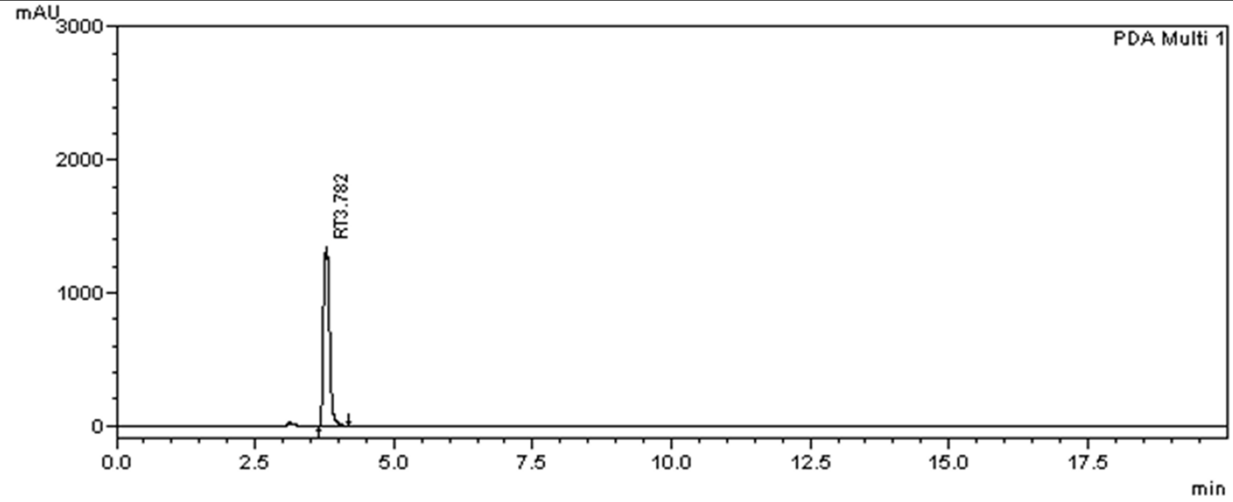

1 PDA Multi 1

<Results>

PDA Ch1 215nm 4nm

Peak#	Ret. Time	Area	Height	Area %	Height %	Resolution
1	3.782	10222472	1341256	100.000	100.000	0.000
Total		10222472	1341256	100.000	100.000	

Figure S15. Purity chromatogram of the **AQQ15**.

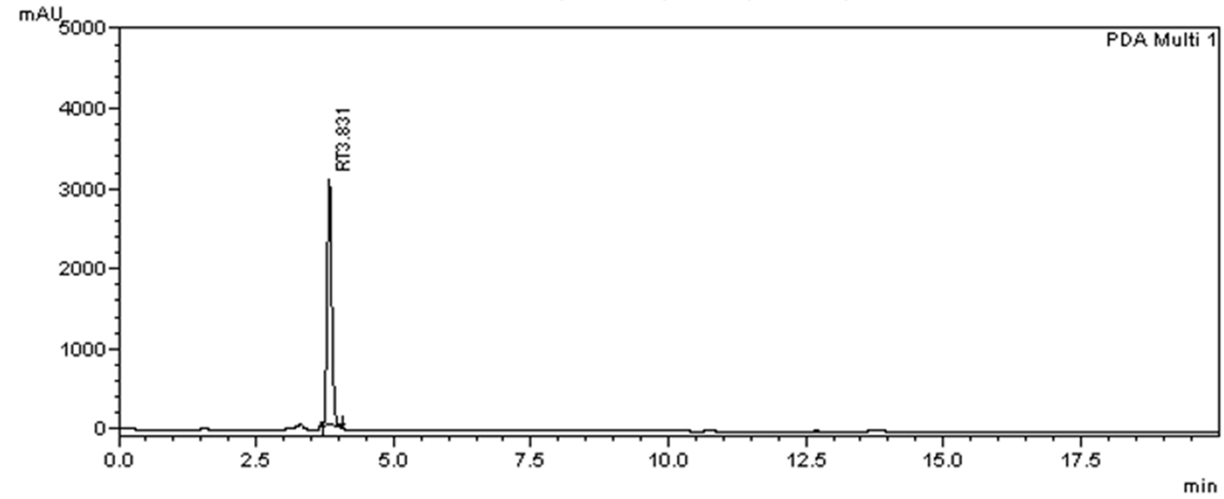

1 PDA Multi 1

<Results>

PDA Ch1 215nm 4nm

Peak#	Ret. Time	Area	Height	Area %	Height %	Resolution
1	3.831	17450189	3036307	100.000	100.000	0.000
Total		17450189	3036307	100.000	100.000	

Figure S16. Purity chromatogram of the **AQQ16**.

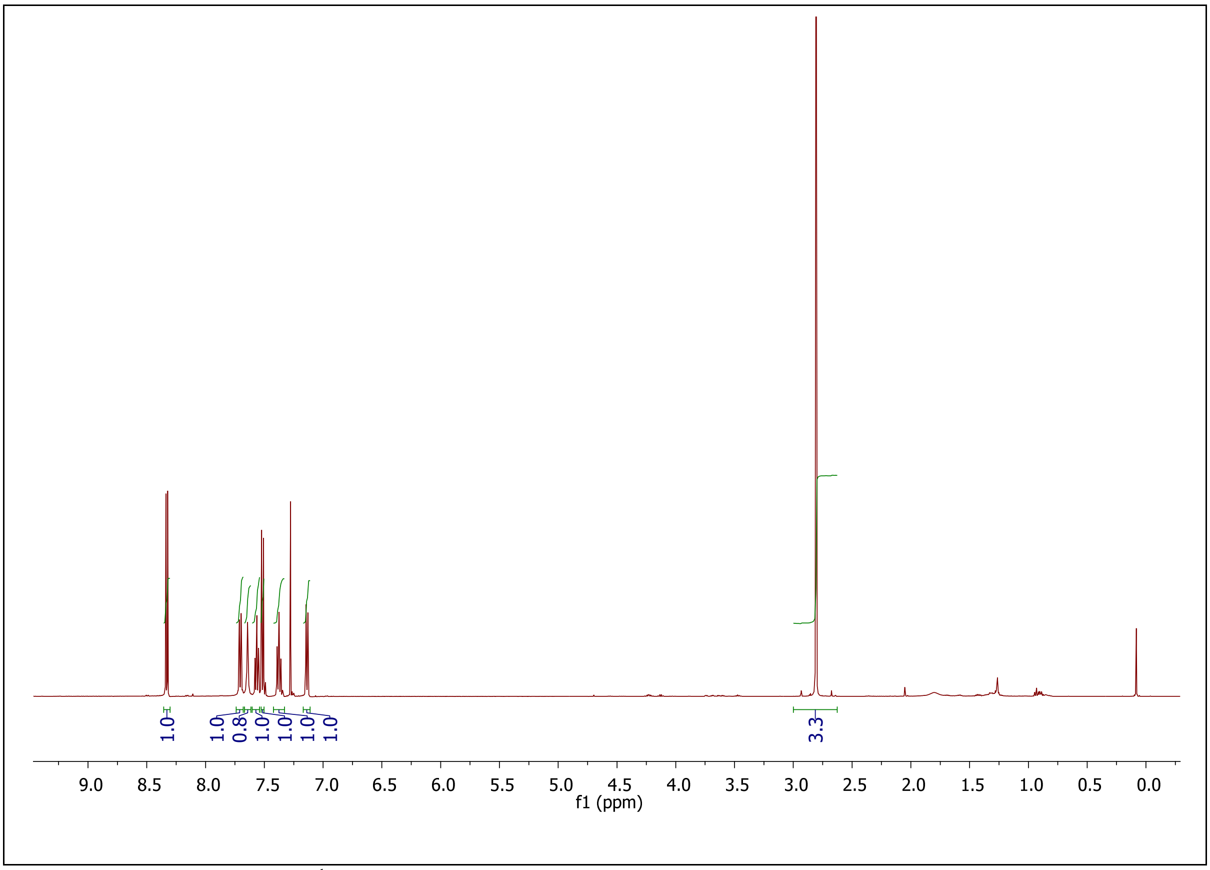

Figure S17. ^{1}H NMR (500 MHz) spectrum of the **AQQ1** in $CDCl_3$-d_1.

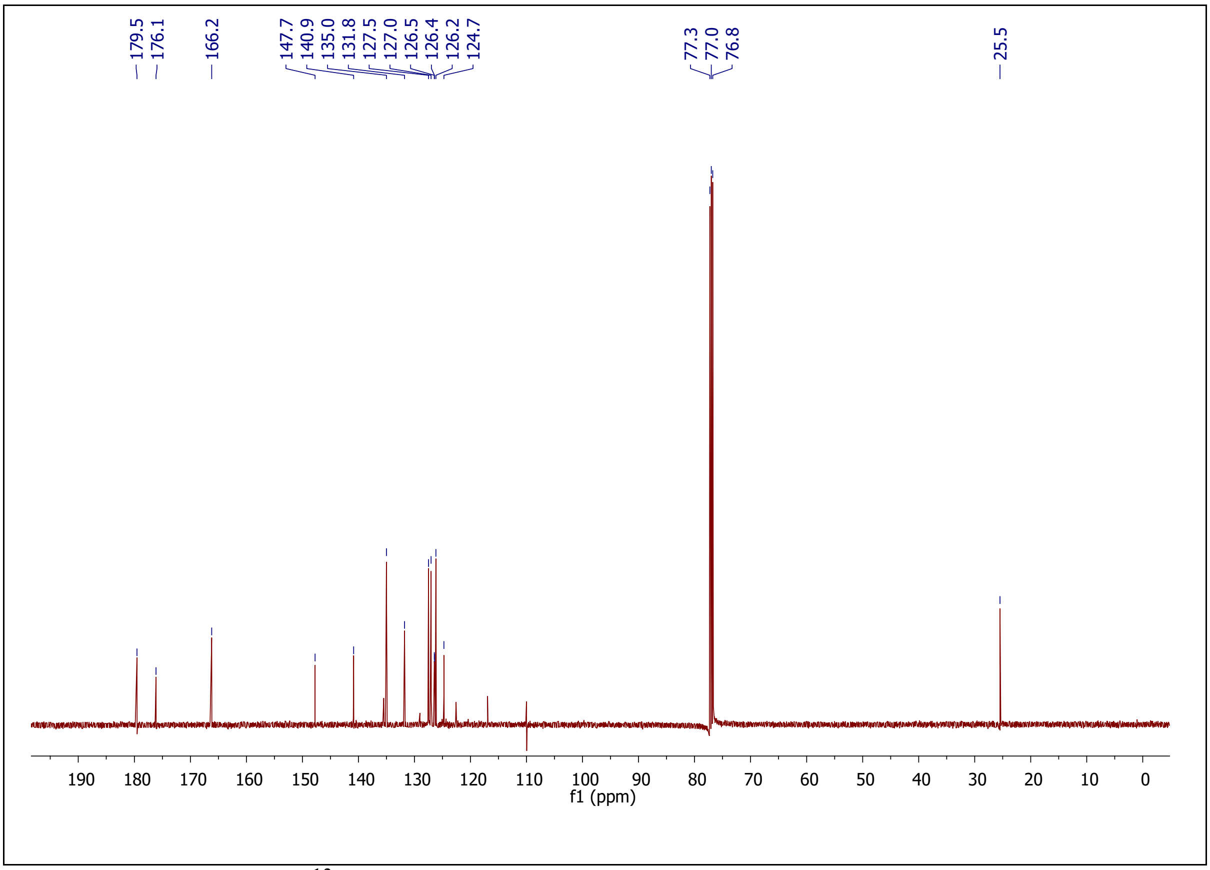

Figure S18. ^{13}C NMR (125 MHz) spectrum of the **AQQ1** in $CDCl_3$-d_1.

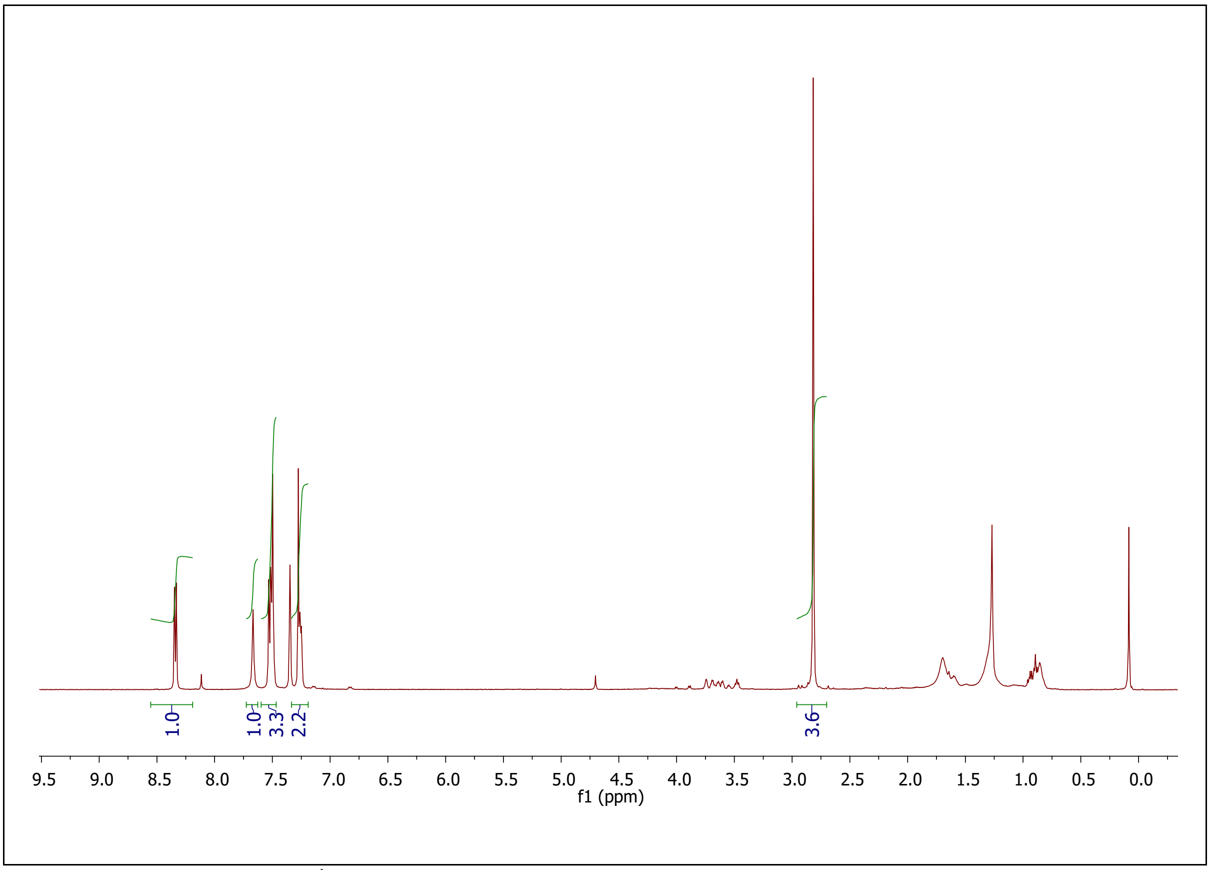

Figure S19. ^{1}H NMR (500 MHz) spectrum of the **AQQ2** in $CDCl_3$-d_1.

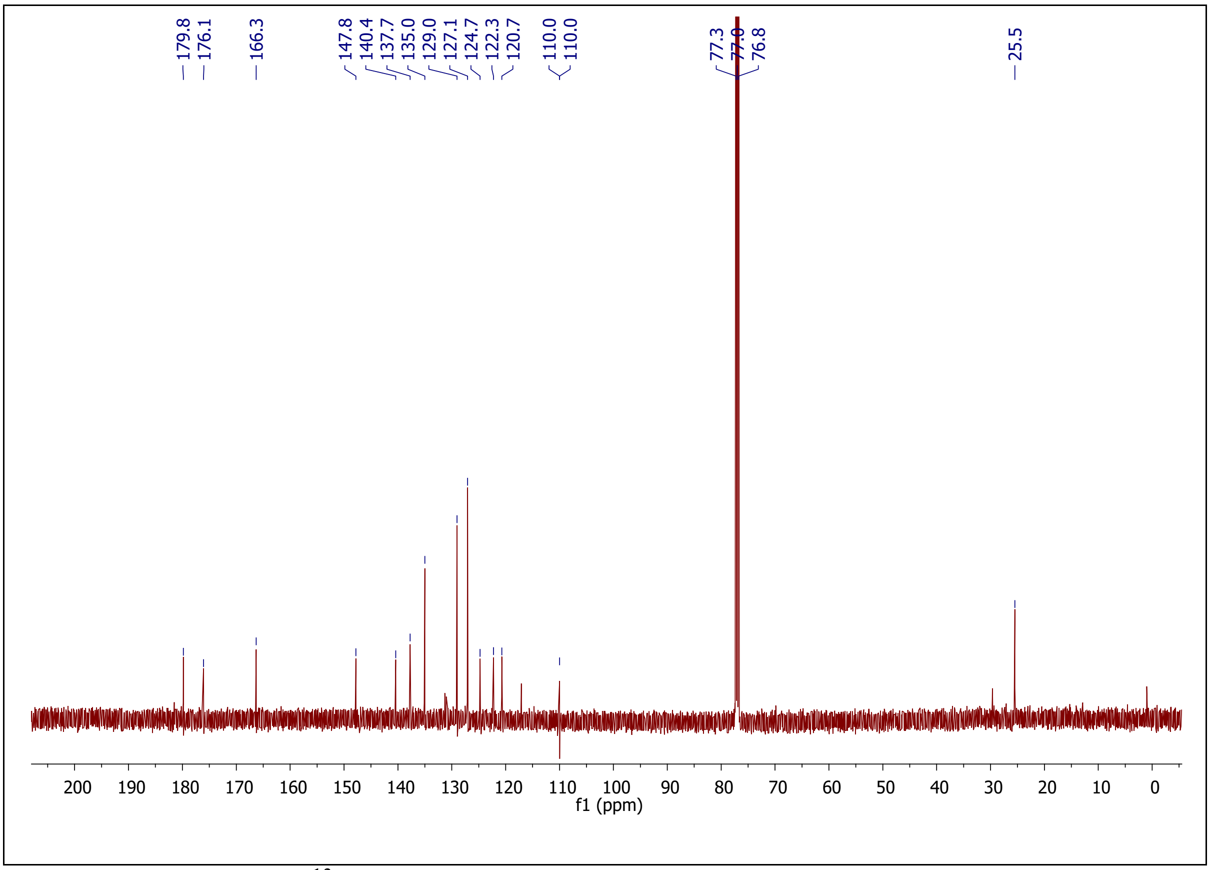

Figure S20. ^{13}C NMR (125 MHz) spectrum of the **AQQ2** in $CDCl_3$-d_1.

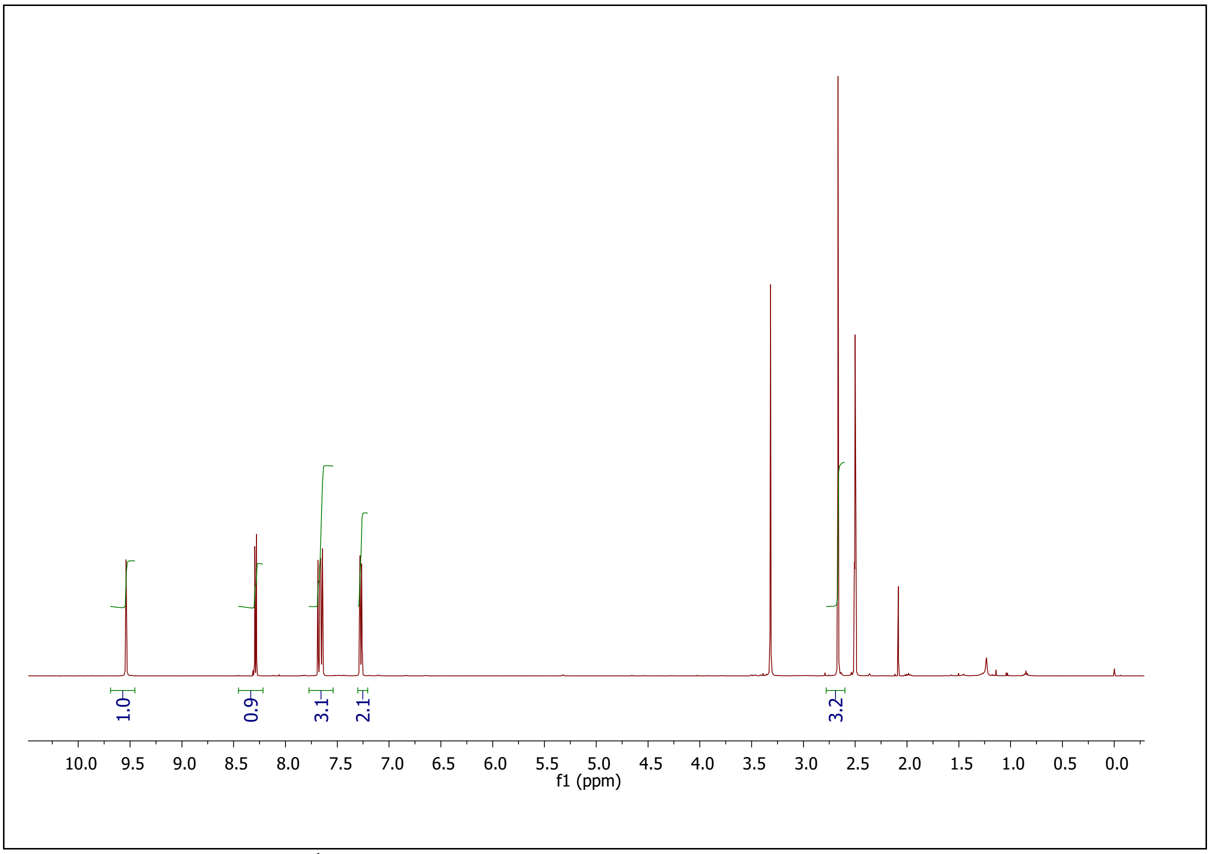

Figure S21. ^{1}H NMR (500 MHz) spectrum of the **AQQ3** in DMSO-d_6.

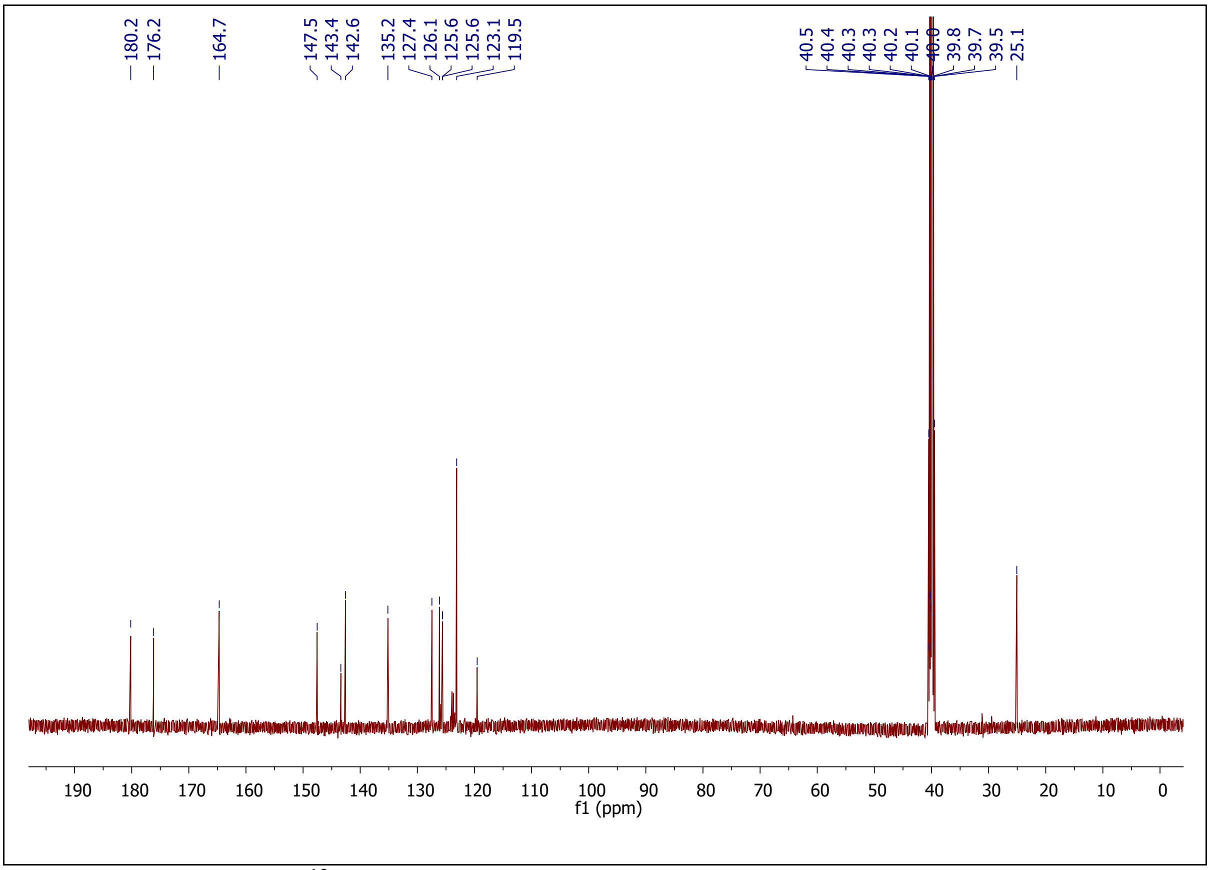

Figure S22. ^{13}C NMR (125 MHz) spectrum of the **AQQ3** in DMSO-d_6.

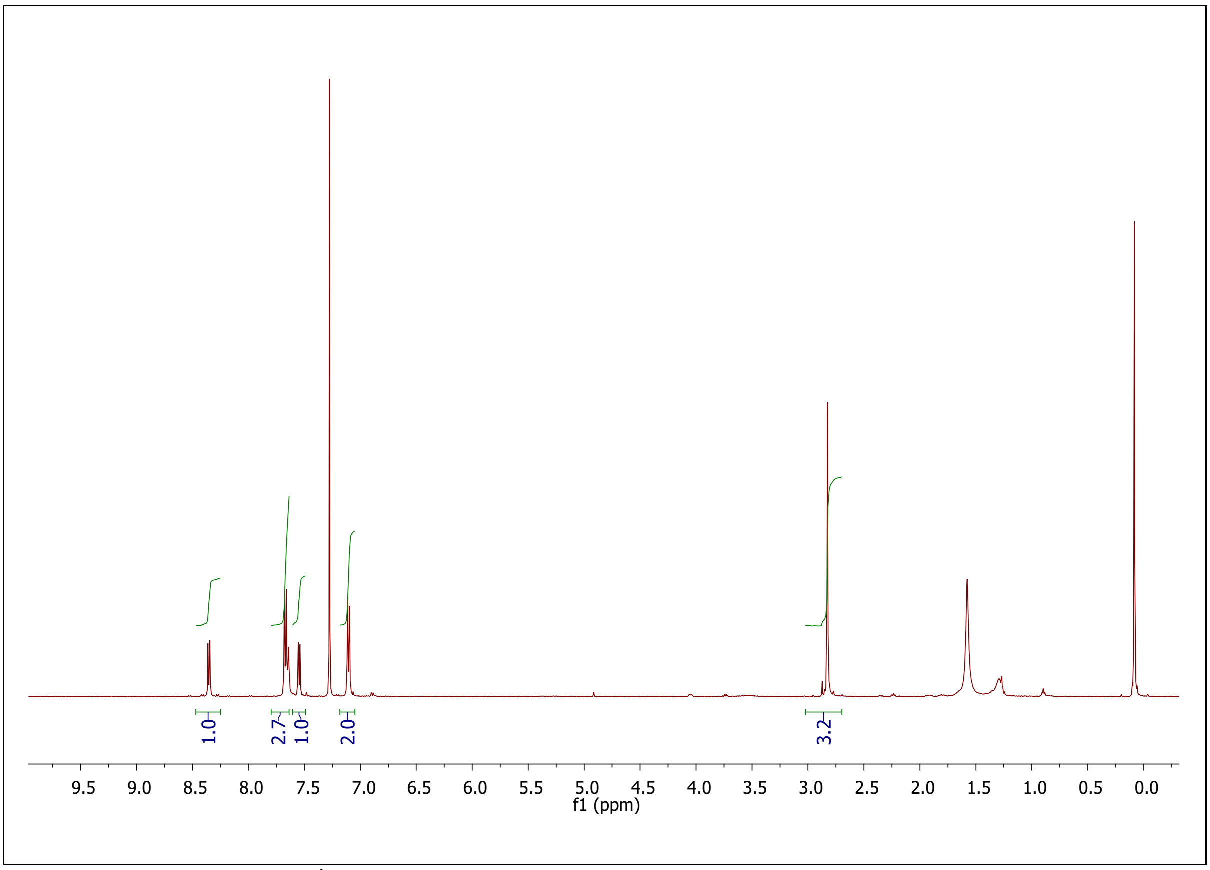

Figure S23. ^{1}H NMR (500 MHz) spectrum of the **AQQ4** in $CDCl_3$-d_1.

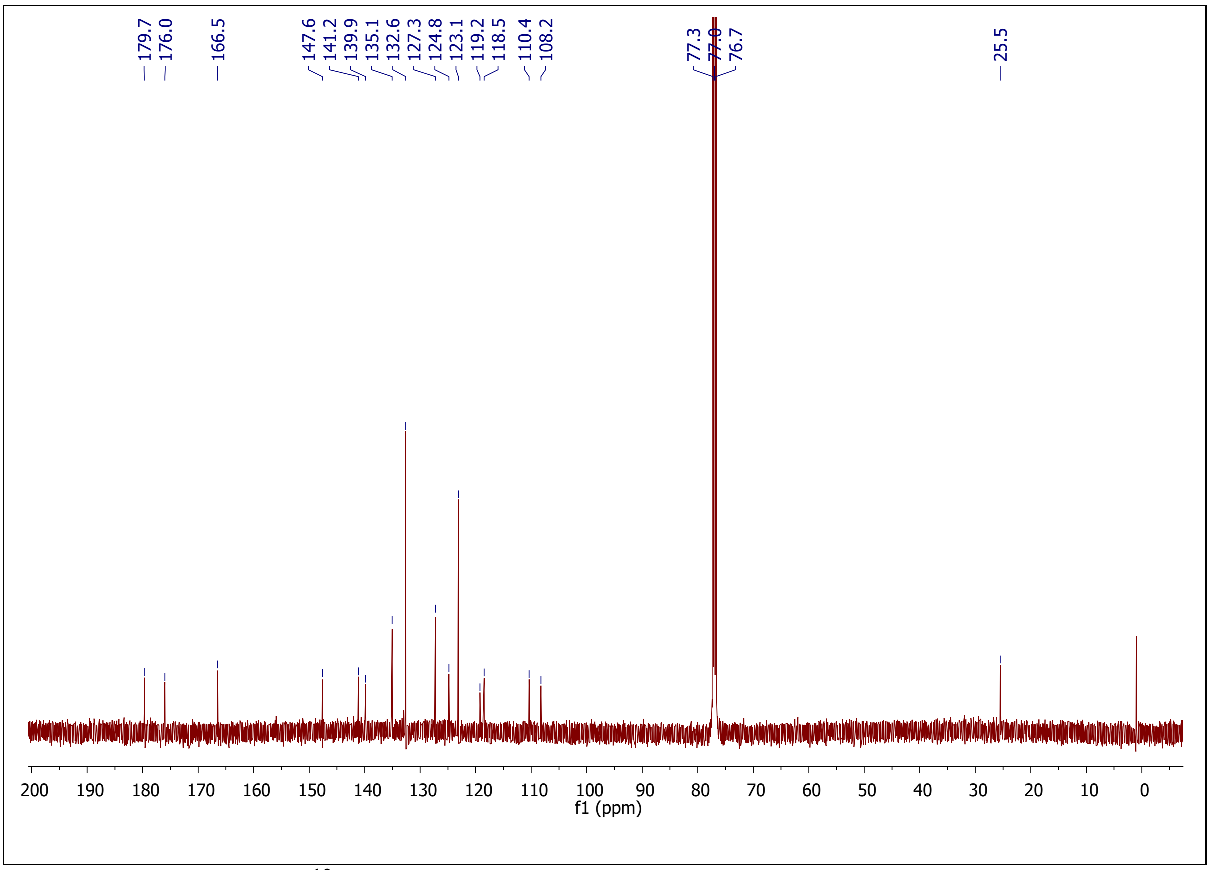

Figure S24. ^{13}C NMR (125 MHz) spectrum of the **AQQ4** in $CDCl_3$-d_1.

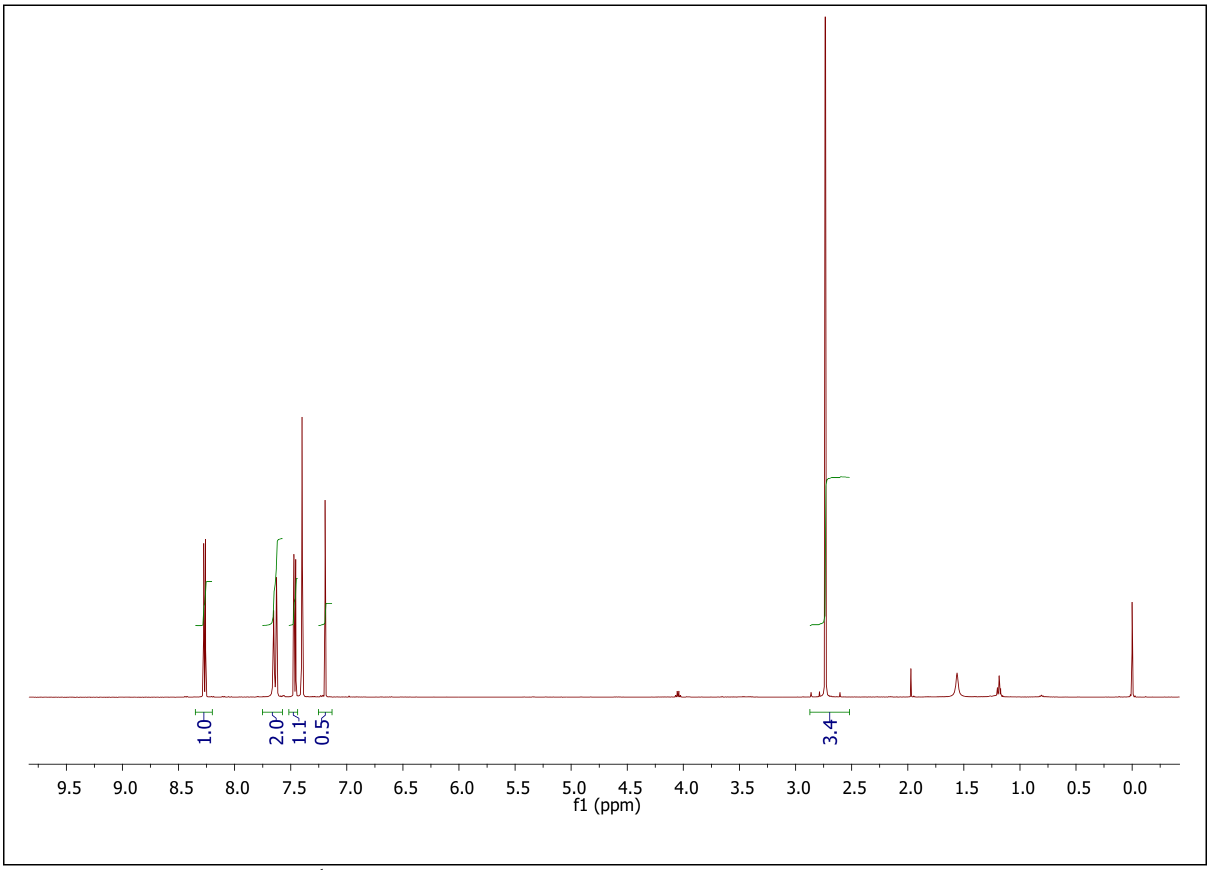

Figure S25. ^{1}H NMR (500 MHz) spectrum of the **AQQ5** in $CDCl_3$-d_1.

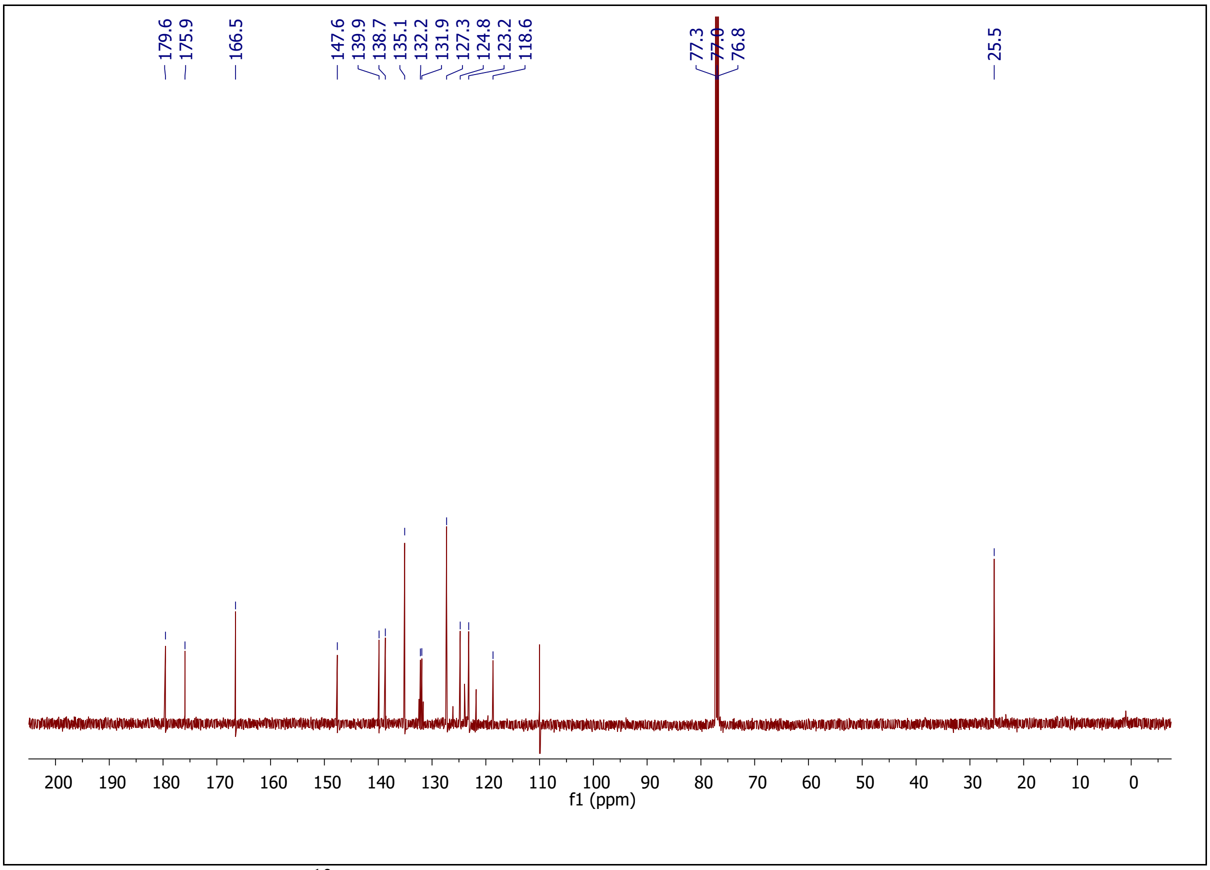

Figure S26. ^{13}C NMR (125 MHz) spectrum of the **AQQ5** in $CDCl_3$-d_1.

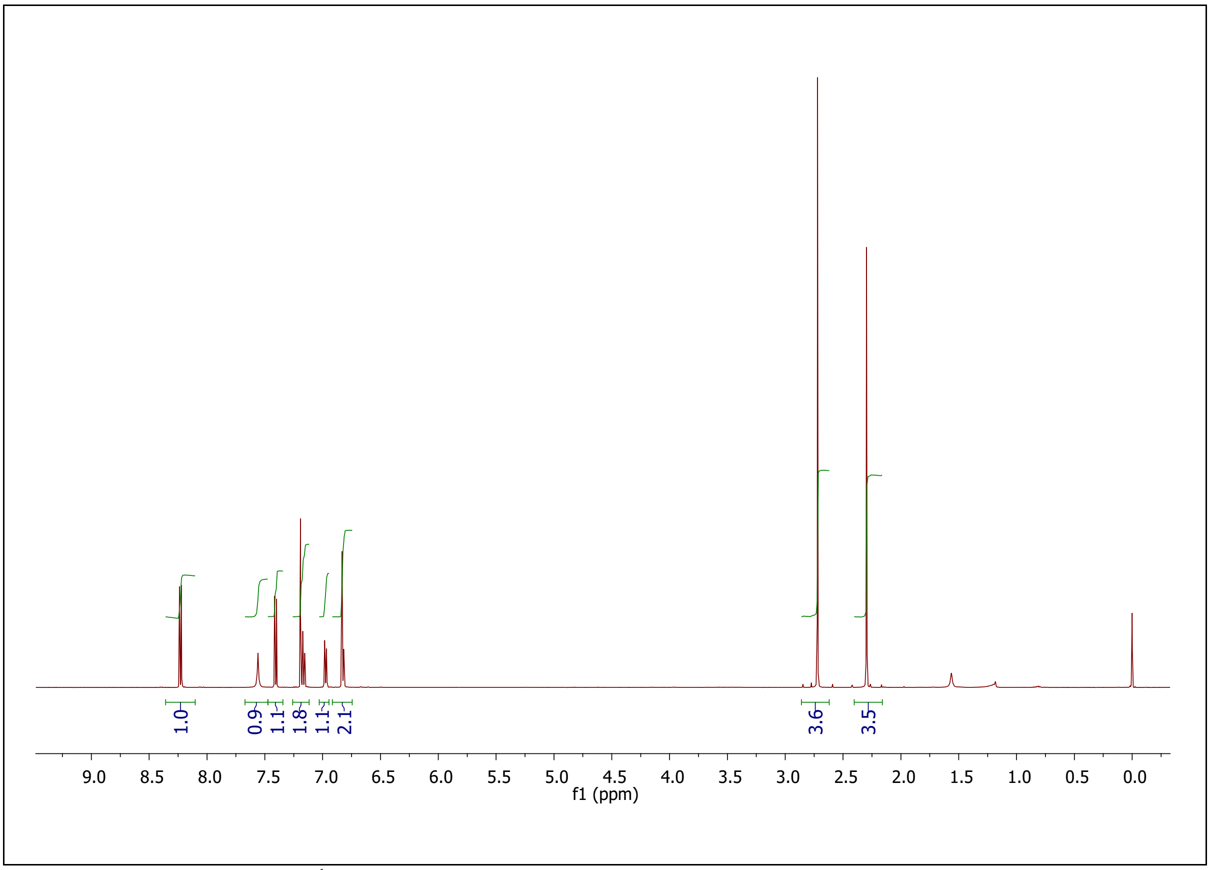

Figure S27. ^{1}H NMR (500 MHz) spectrum of the **AQQ6** in $CDCl_3$-d_1.

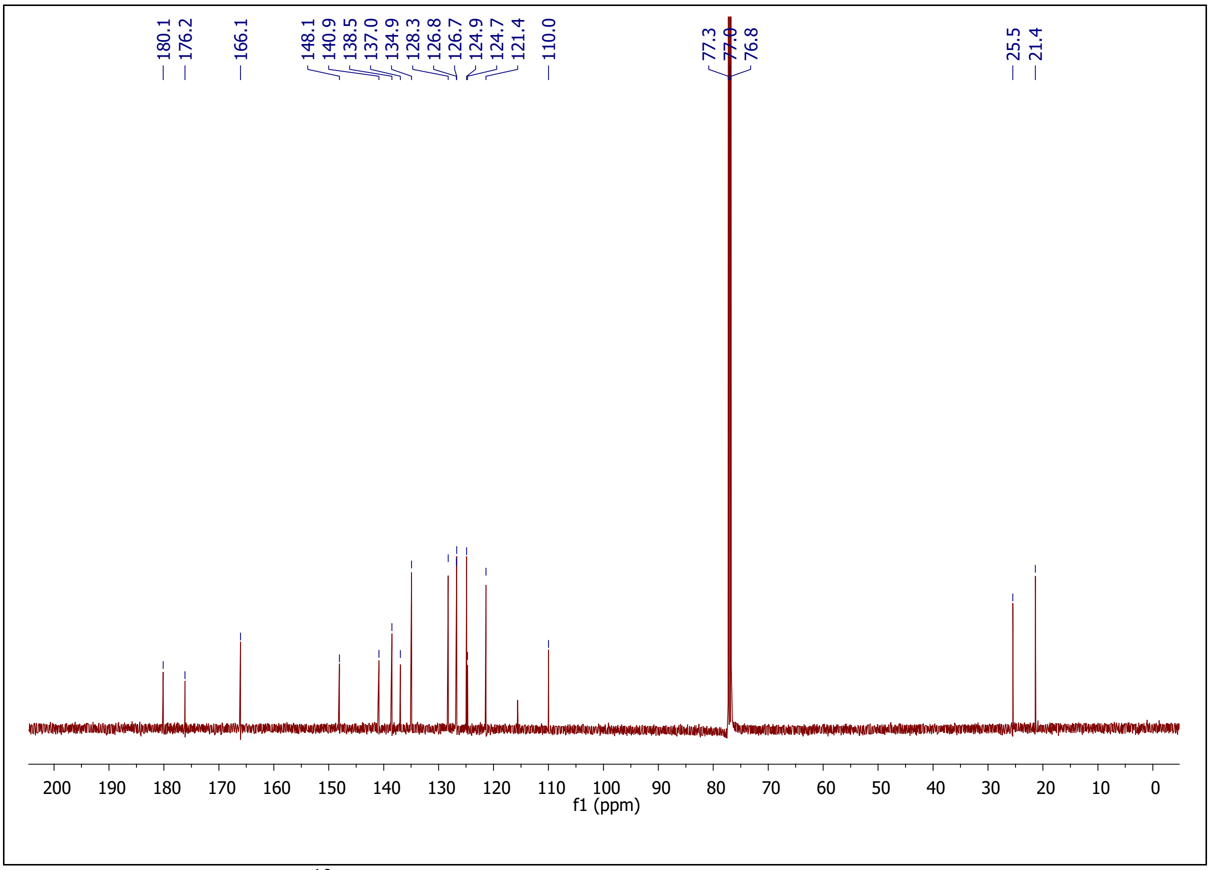

Figure S28. ^{13}C NMR (125 MHz) spectrum of the **AQQ6** in $CDCl_3$-d_1.

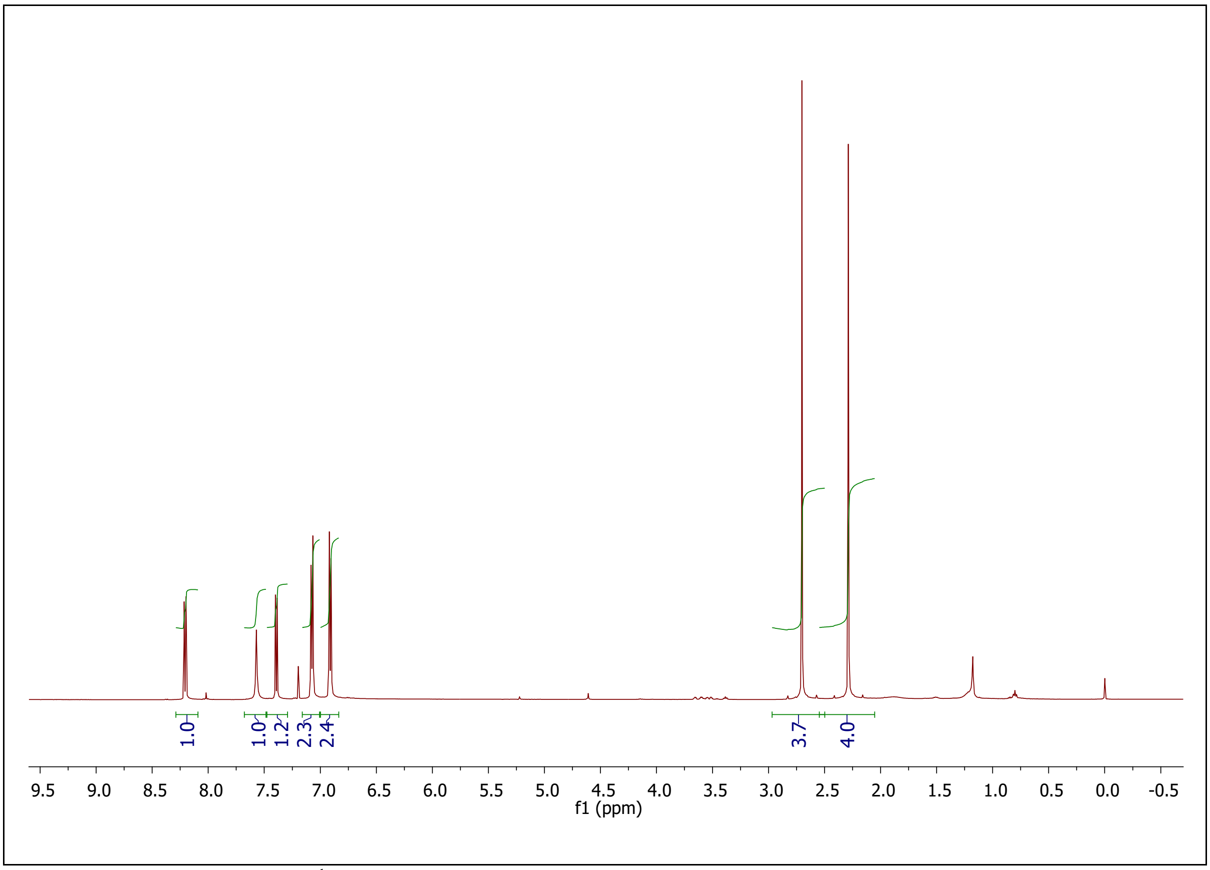

Figure S29. ^{1}H NMR (500 MHz) spectrum of the **AQQ7** in $CDCl_3$-d_1.

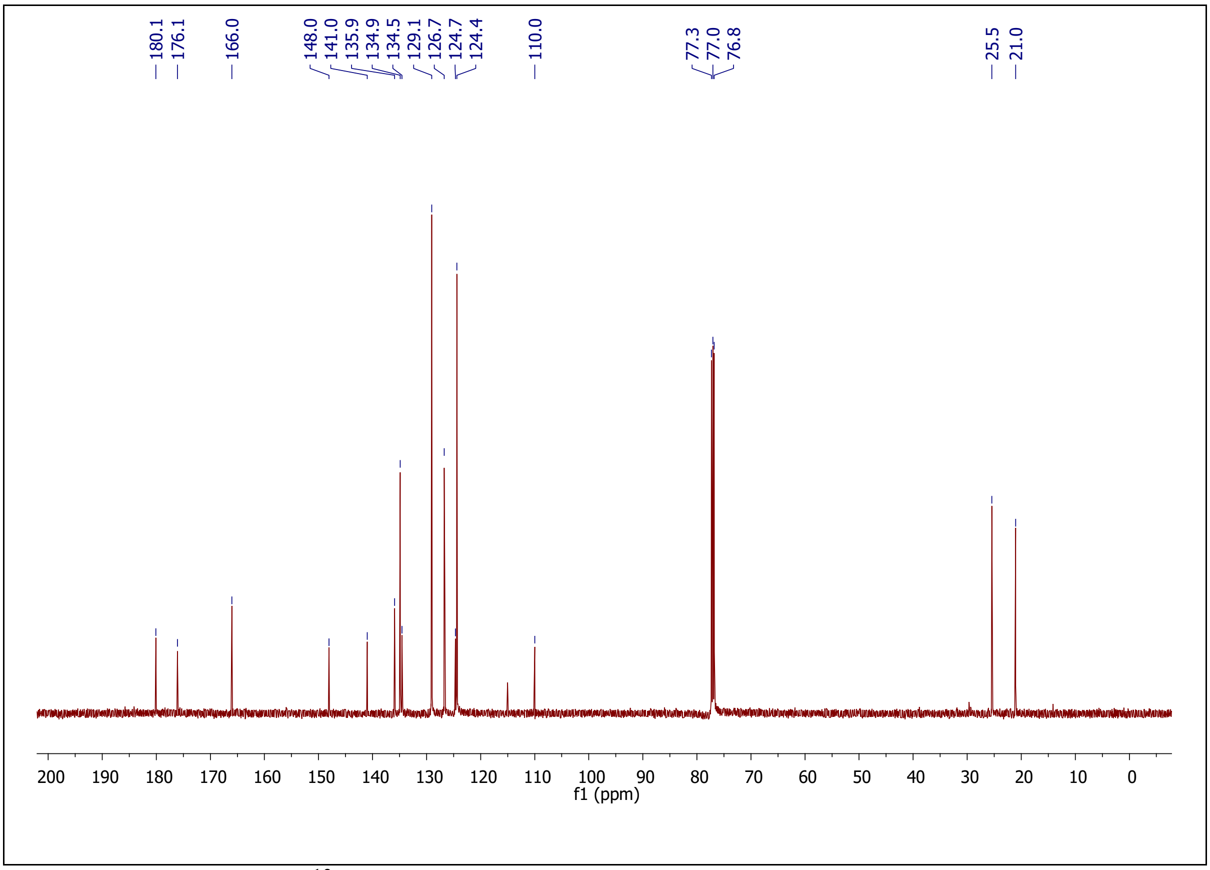

Figure S30. ^{13}C NMR (125 MHz) spectrum of the **AQQ7** in $CDCl_3$-d_1.

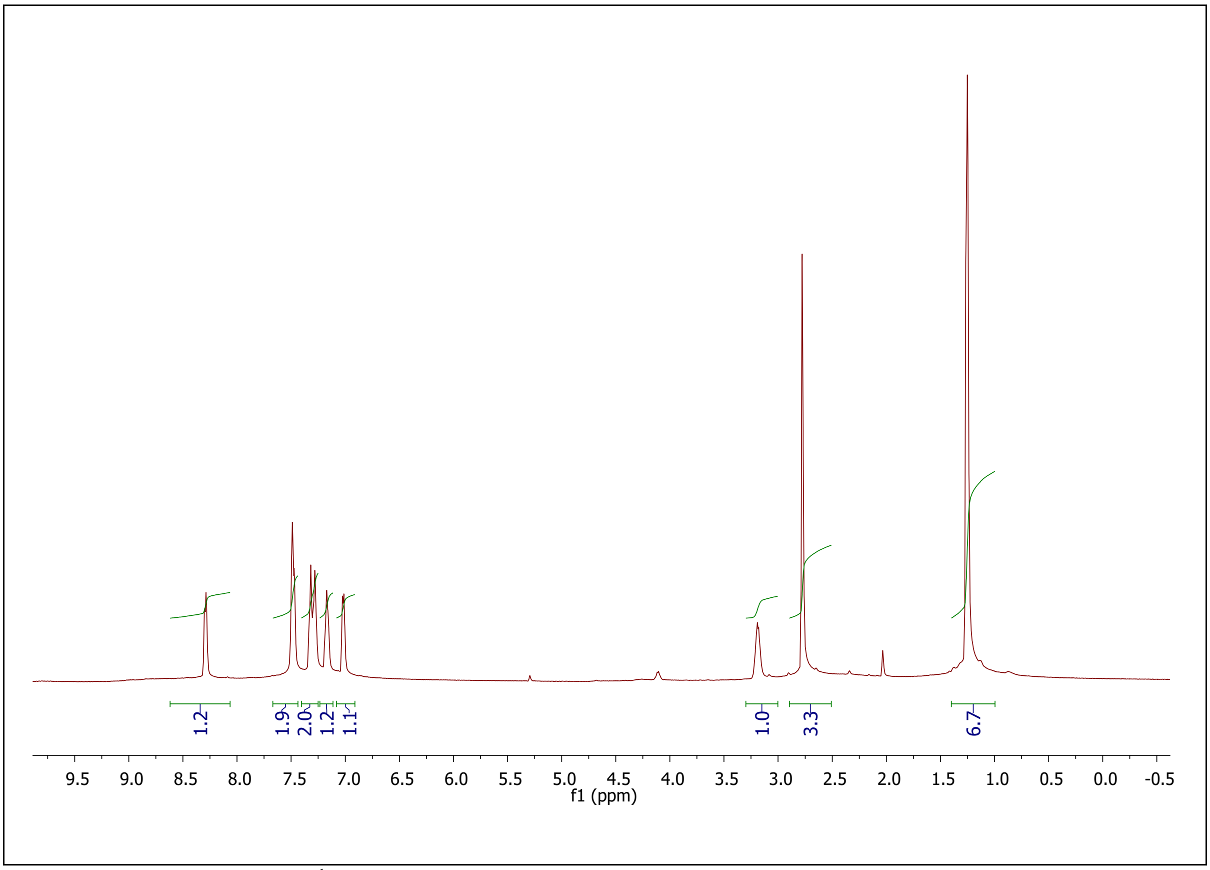

Figure S31. ^{1}H NMR (500 MHz) spectrum of the **AQQ8** in $CDCl_3$-*d*$_1$.

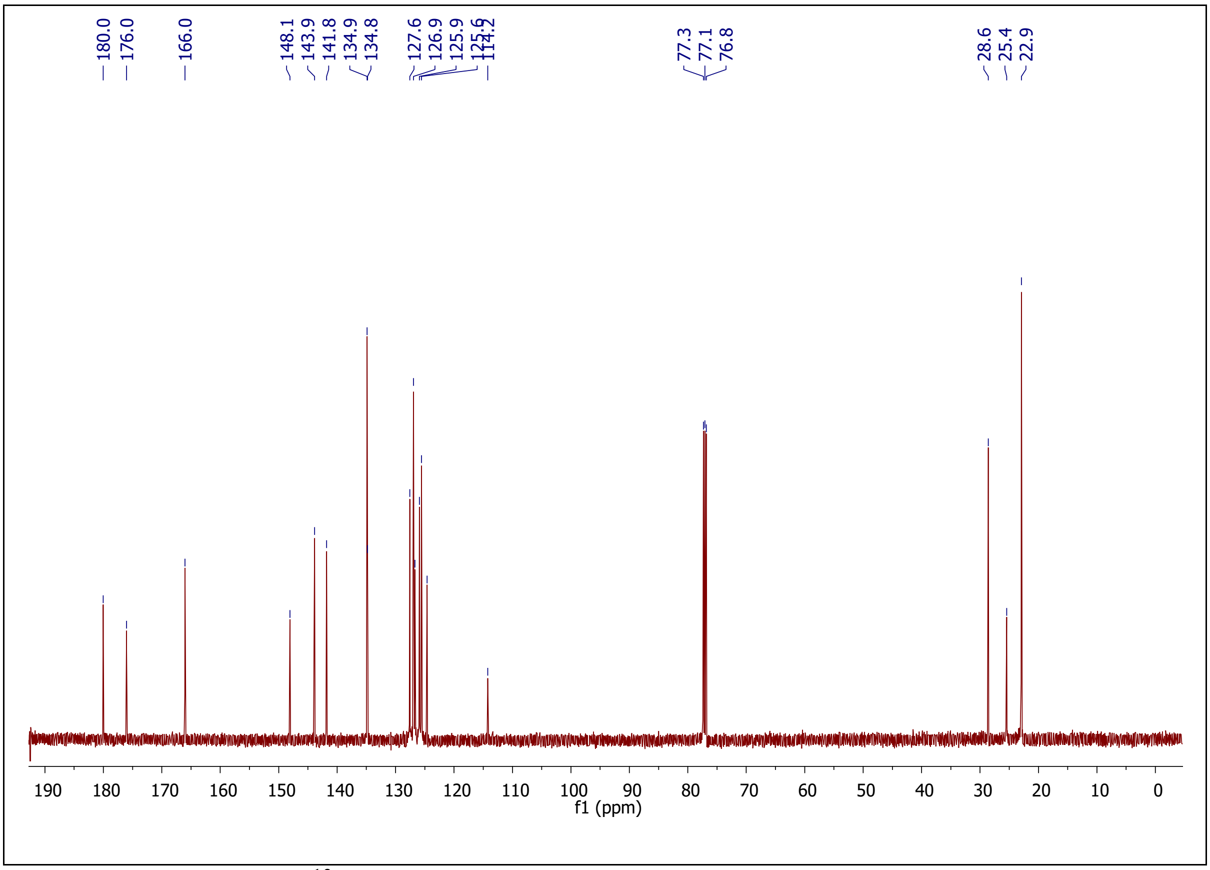

Figure S32. ^{13}C NMR (125 MHz) spectrum of the **AQQ8** in $CDCl_3$-*d*$_1$.

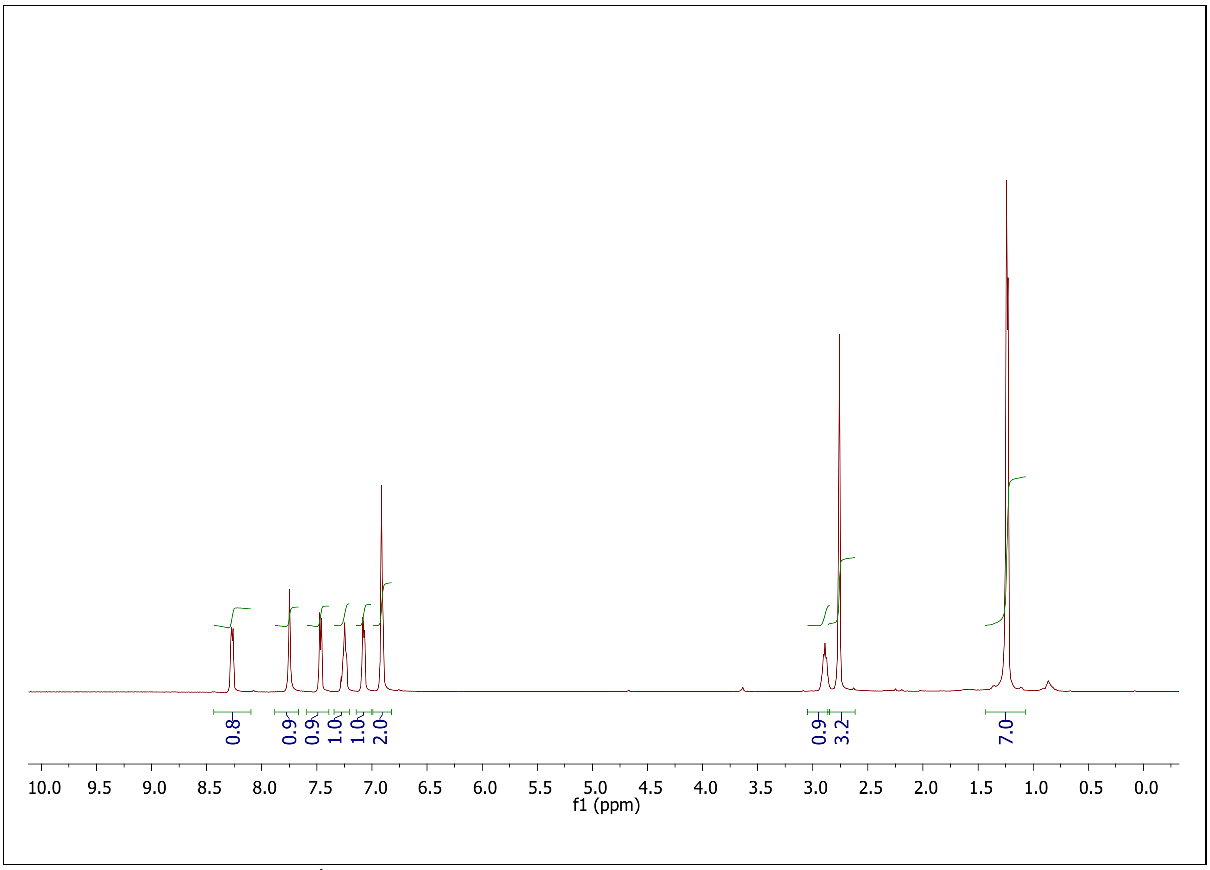

Figure S33. ^{1}H NMR (500 MHz) spectrum of the **AQQ9** in $CDCl_3$-*d*$_1$.

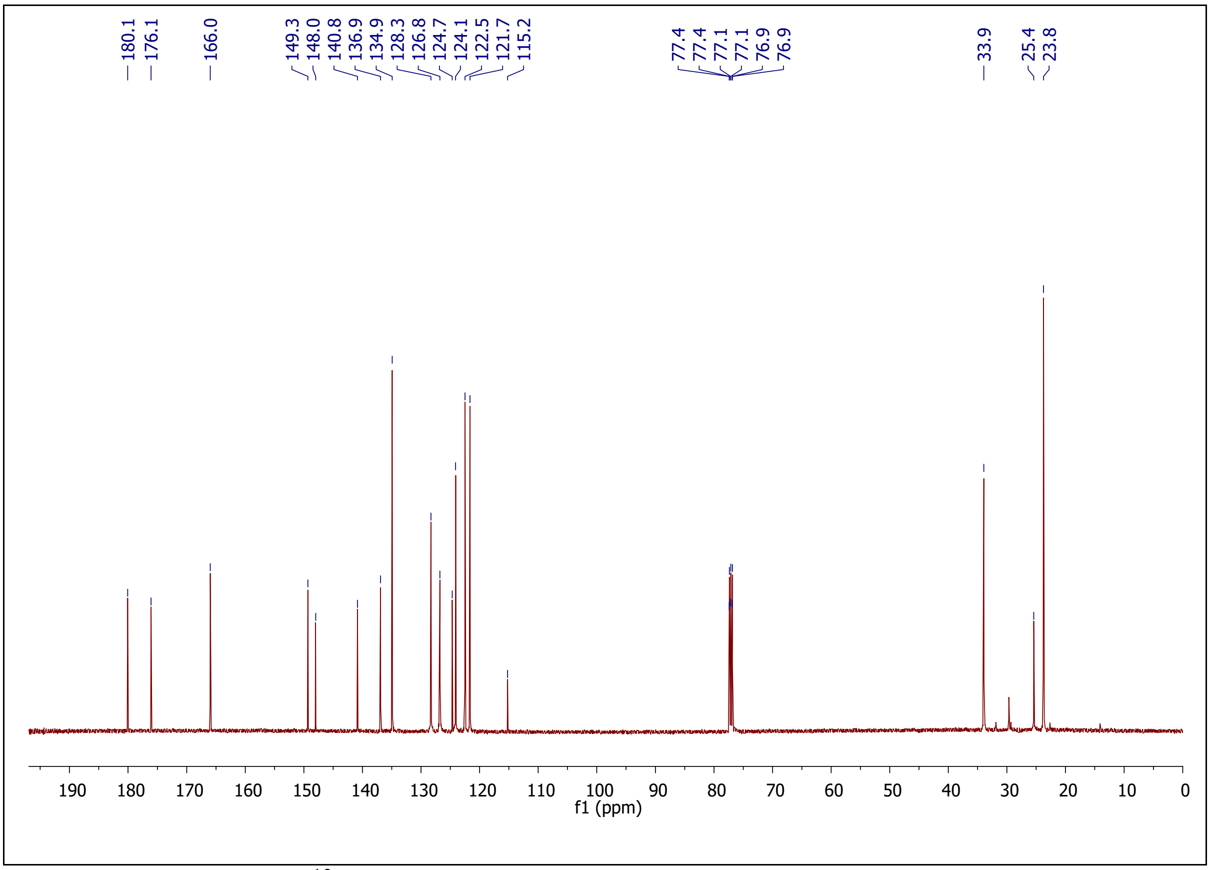

Figure S34. ^{13}C NMR (125 MHz) spectrum of the **AQQ9** in $CDCl_3$-*d*$_1$.

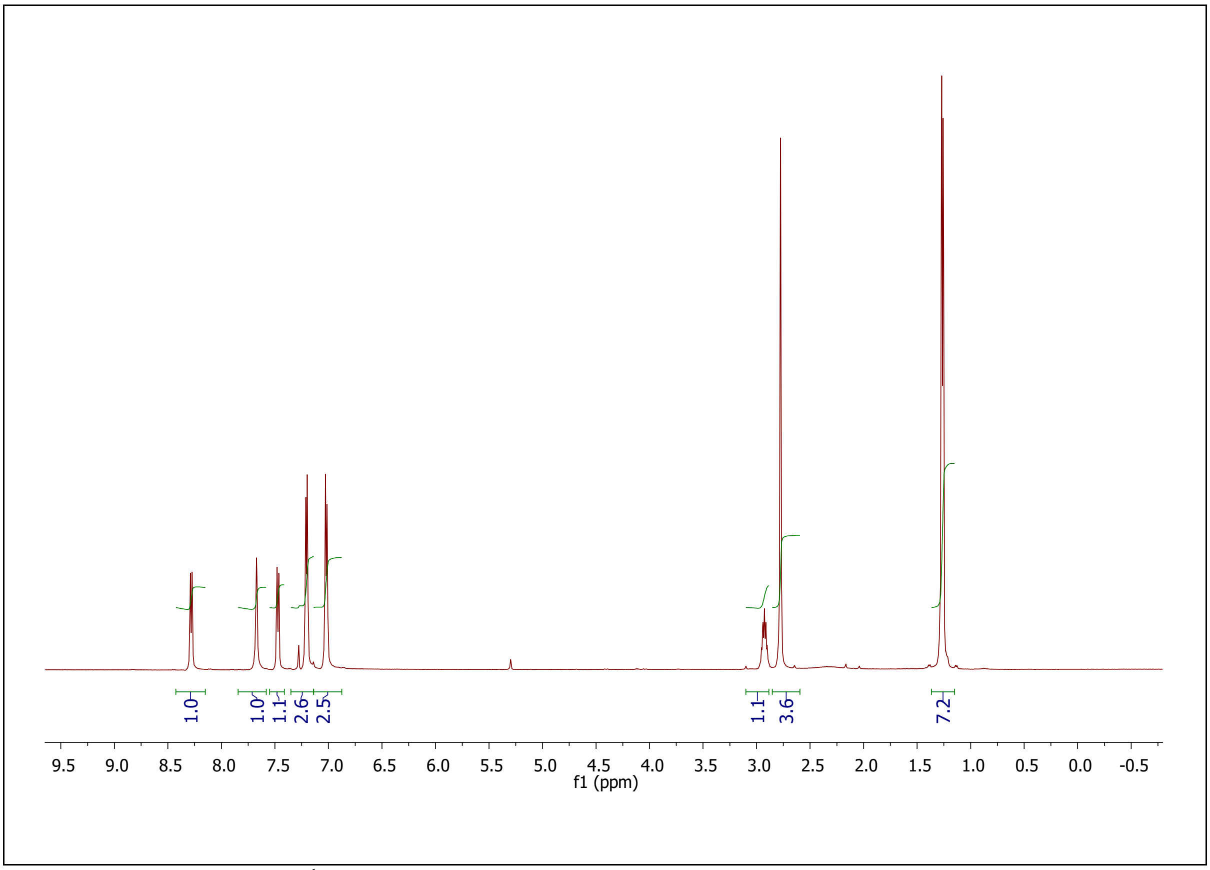

Figure S35. ^{1}H NMR (500 MHz) spectrum of the **AQQ10** in $CDCl_3$-d_1.

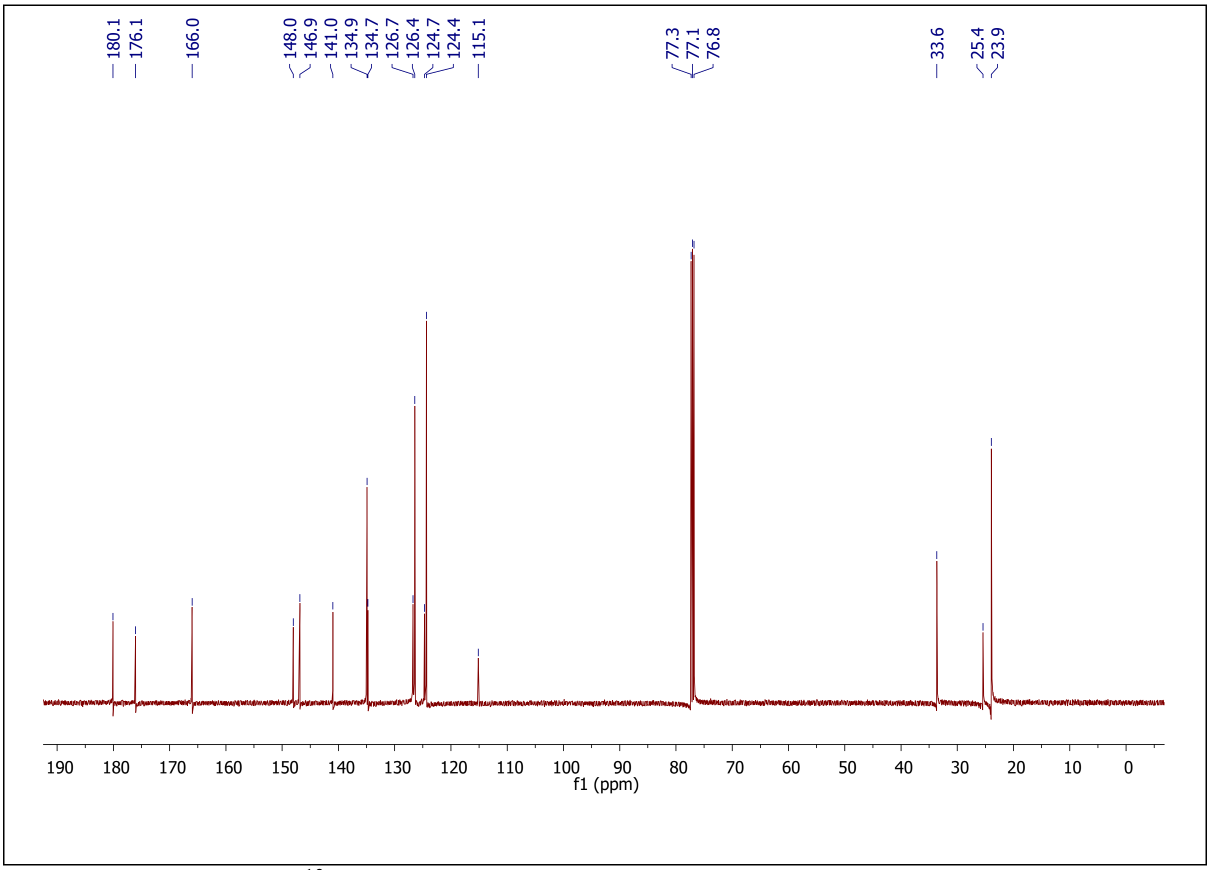

Figure S36. ^{13}C NMR (125 MHz) spectrum of the **AQQ10** in $CDCl_3$-d_1.

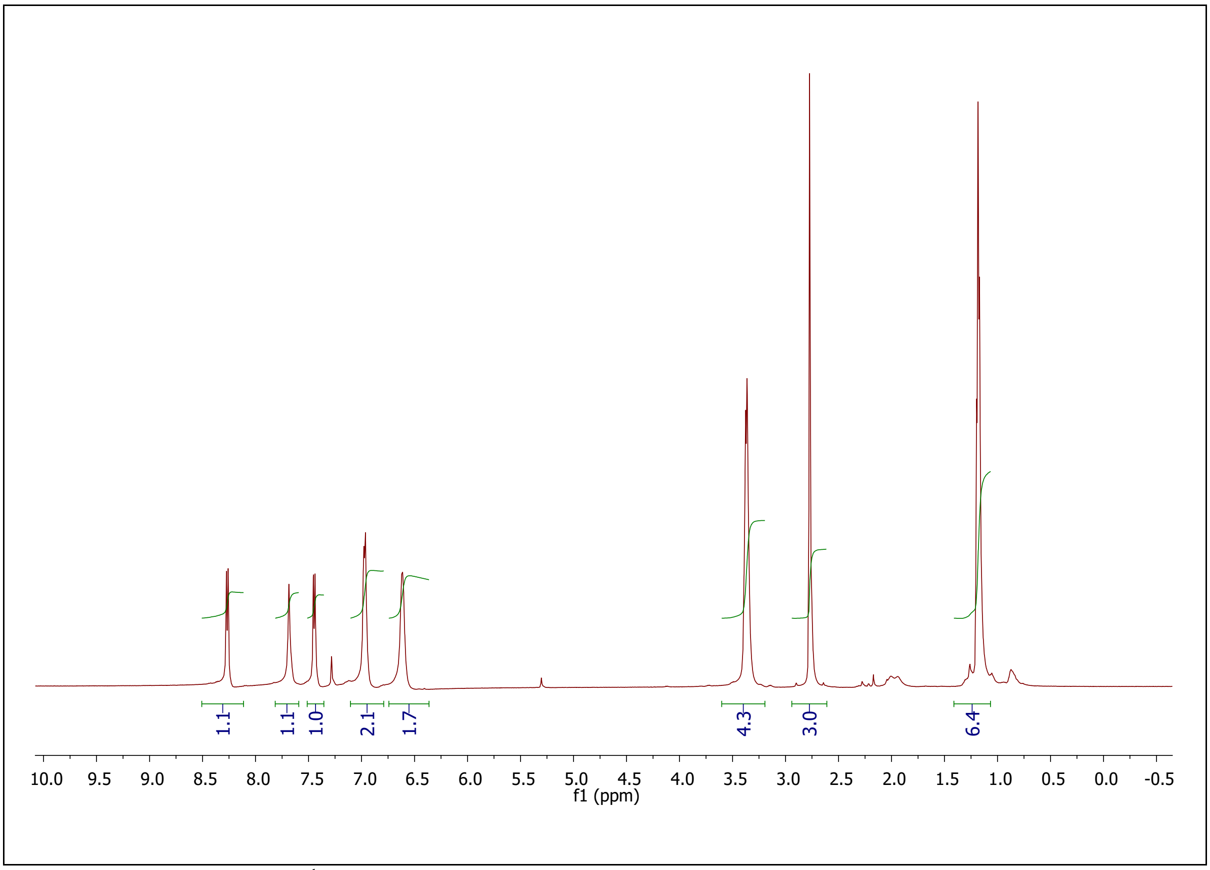

Figure S37. ^{1}H NMR (500 MHz) spectrum of the **AQQ11** in $CDCl_3$-d_1.

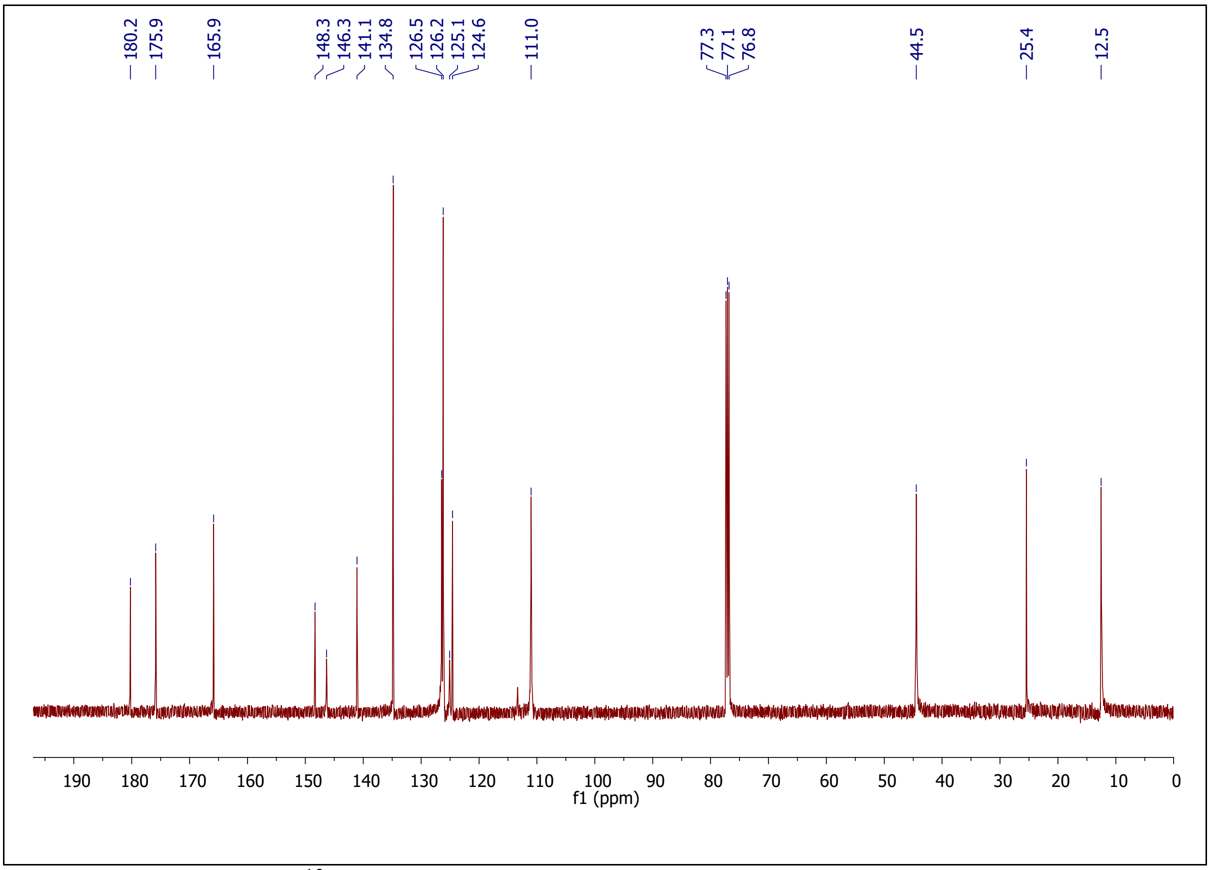

Figure S38. ^{13}C NMR (125 MHz) spectrum of the **AQQ11** in $CDCl_3$-d_1.

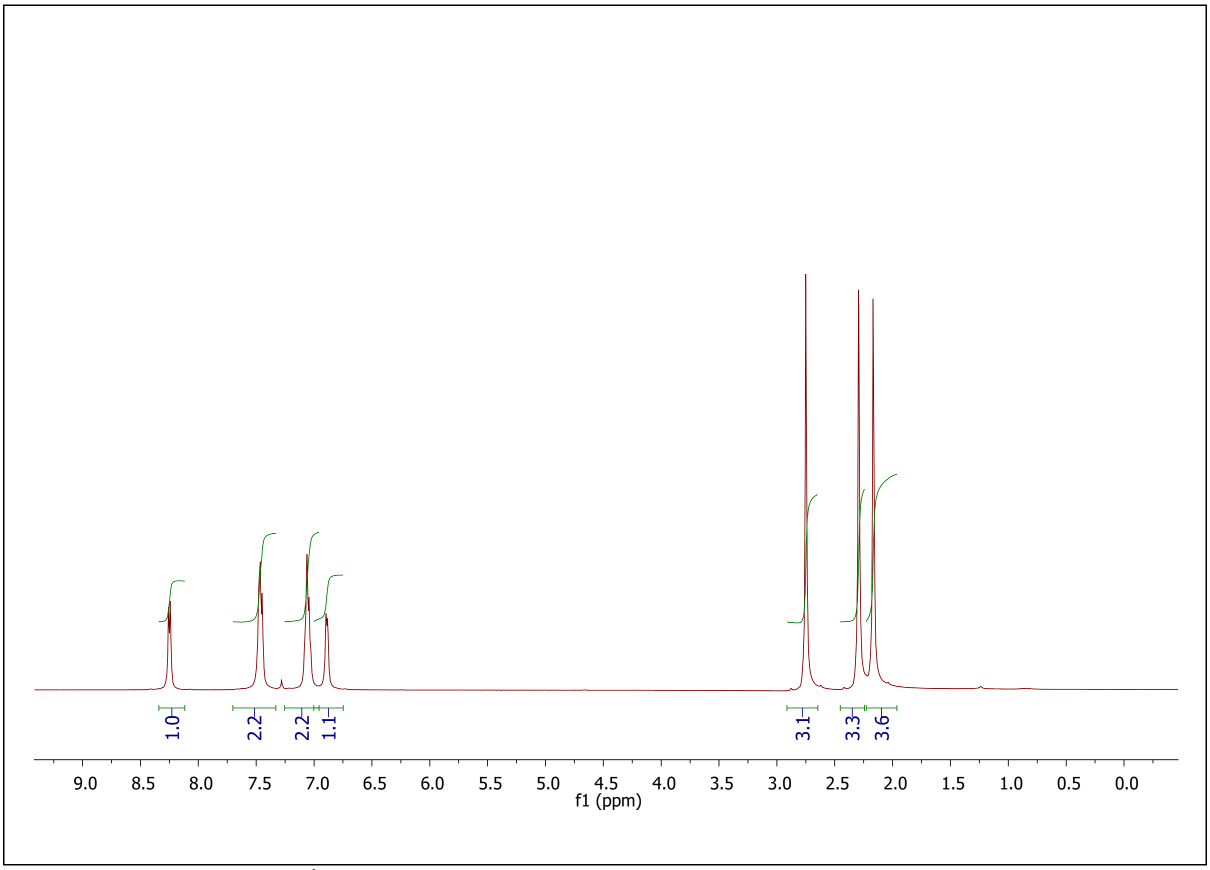

Figure S39. ^{1}H NMR (500 MHz) spectrum of the **AQQ12** in $CDCl_3$-d_1.

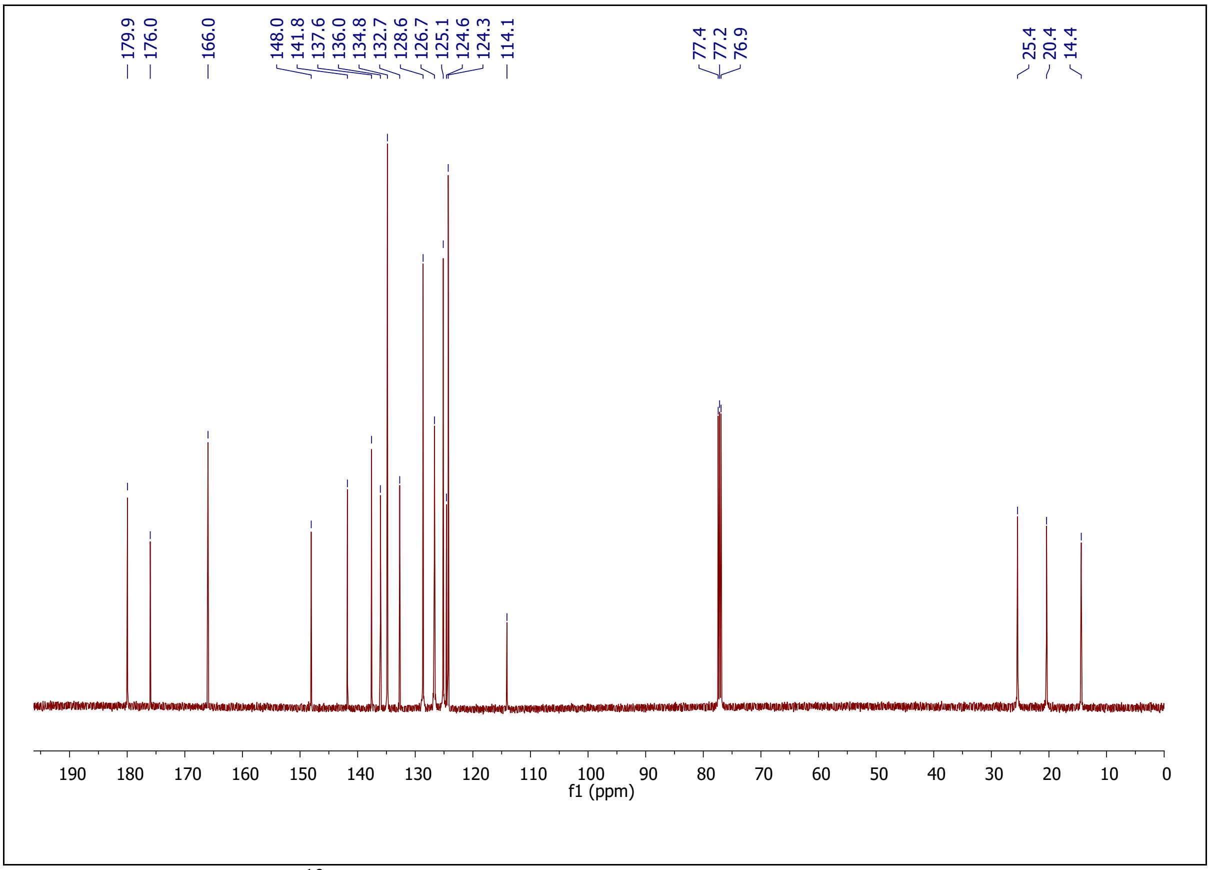

Figure S40. ^{13}C NMR (125 MHz) spectrum of the **AQQ12** in $CDCl_3$-d_1.

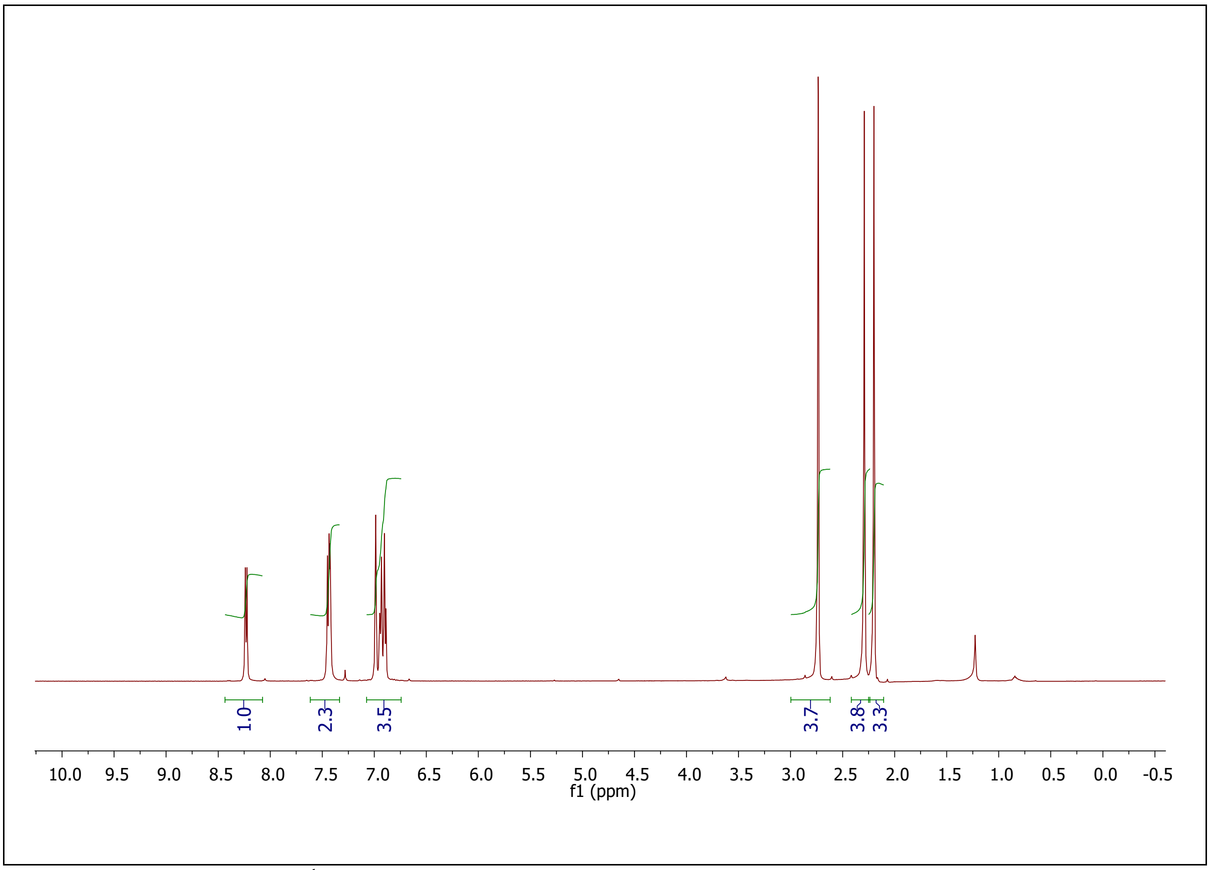

Figure S41. ^{1}H NMR (500 MHz) spectrum of the **AQQ13** in $CDCl_3$-d_1.

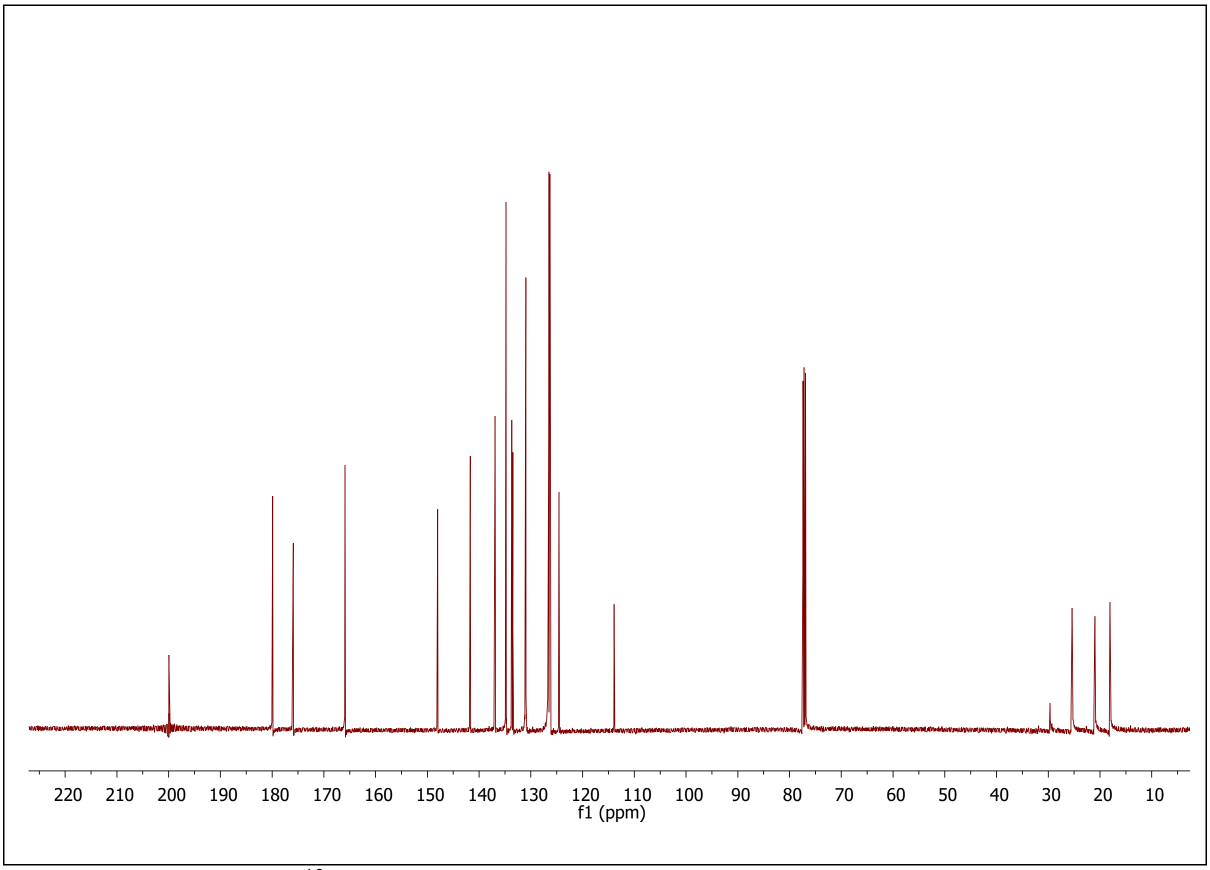

Figure S42. ^{13}C NMR (125 MHz) spectrum of the **AQQ13** in $CDCl_3$-d_1.

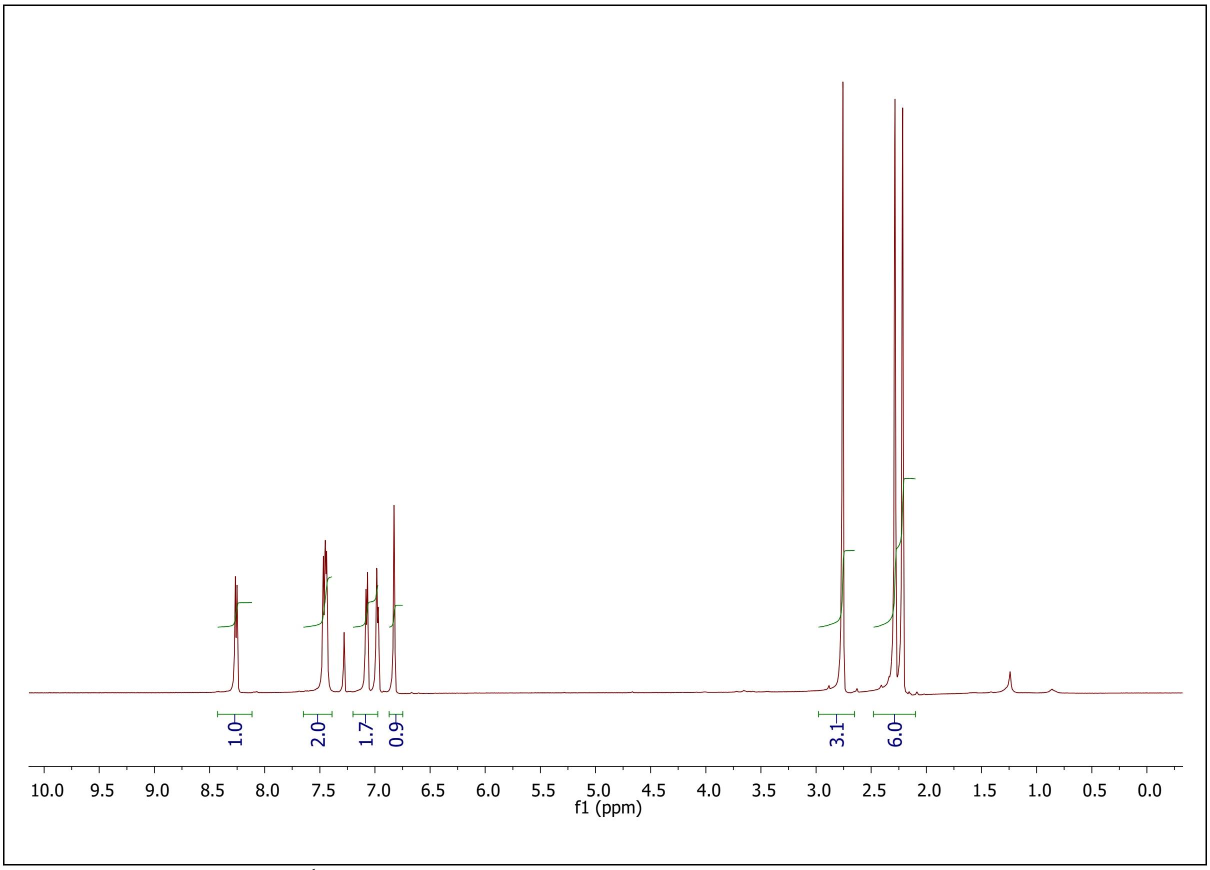

Figure S43. ^{1}H NMR (500 MHz) spectrum of the **AQQ14** in $CDCl_3$-d_1.

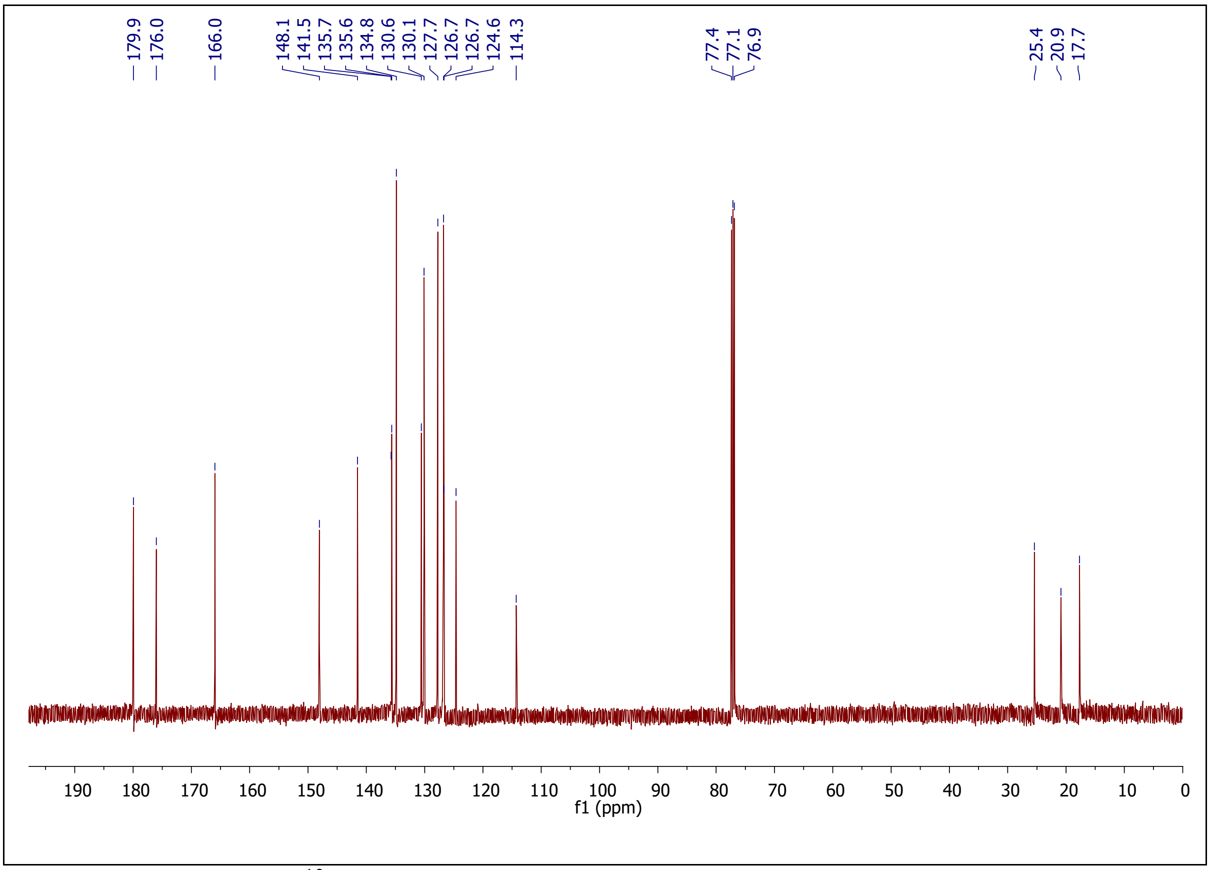

Figure S44. ^{13}C NMR (125 MHz) spectrum of the **AQQ14** in $CDCl_3$-d_1.

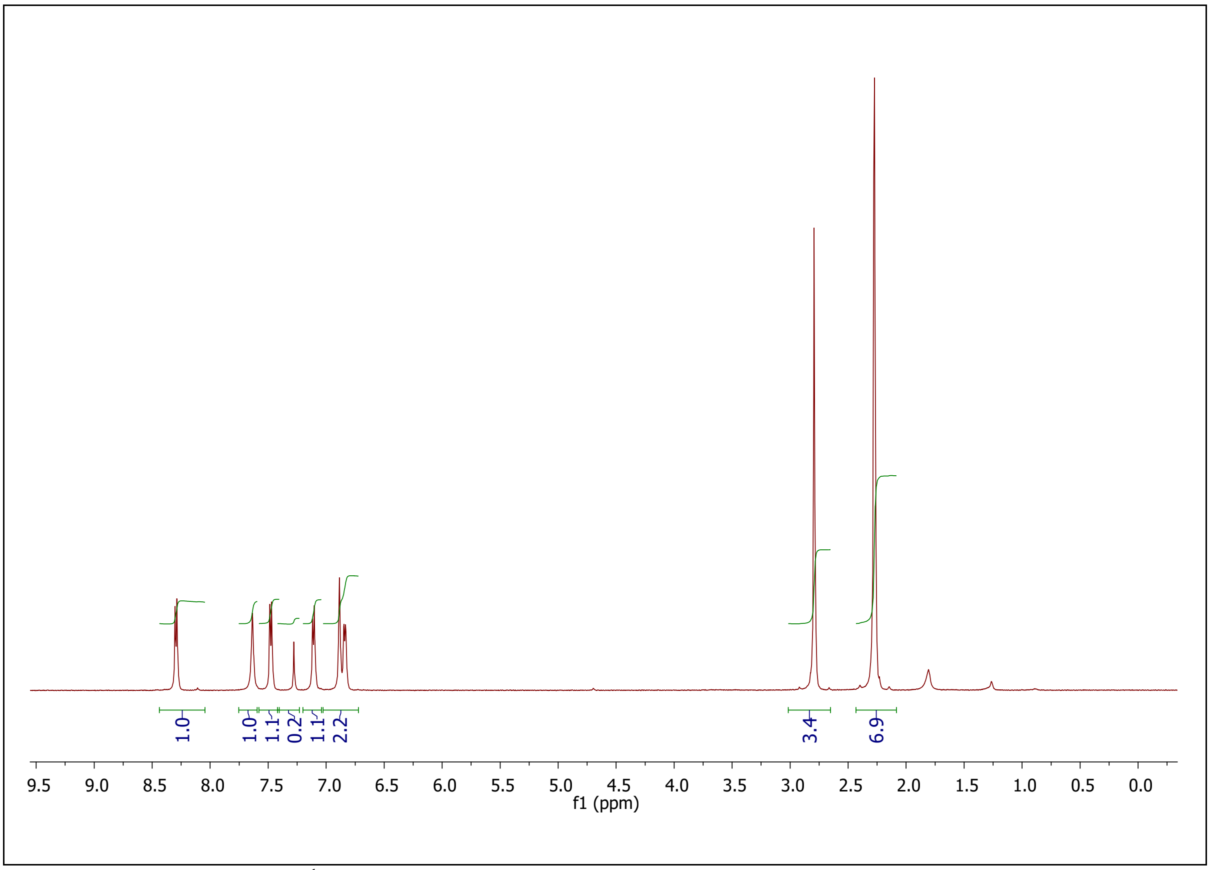

Figure S45. ^{1}H NMR (500 MHz) spectrum of the **AQQ15** in $CDCl_3$-*d*$_1$.

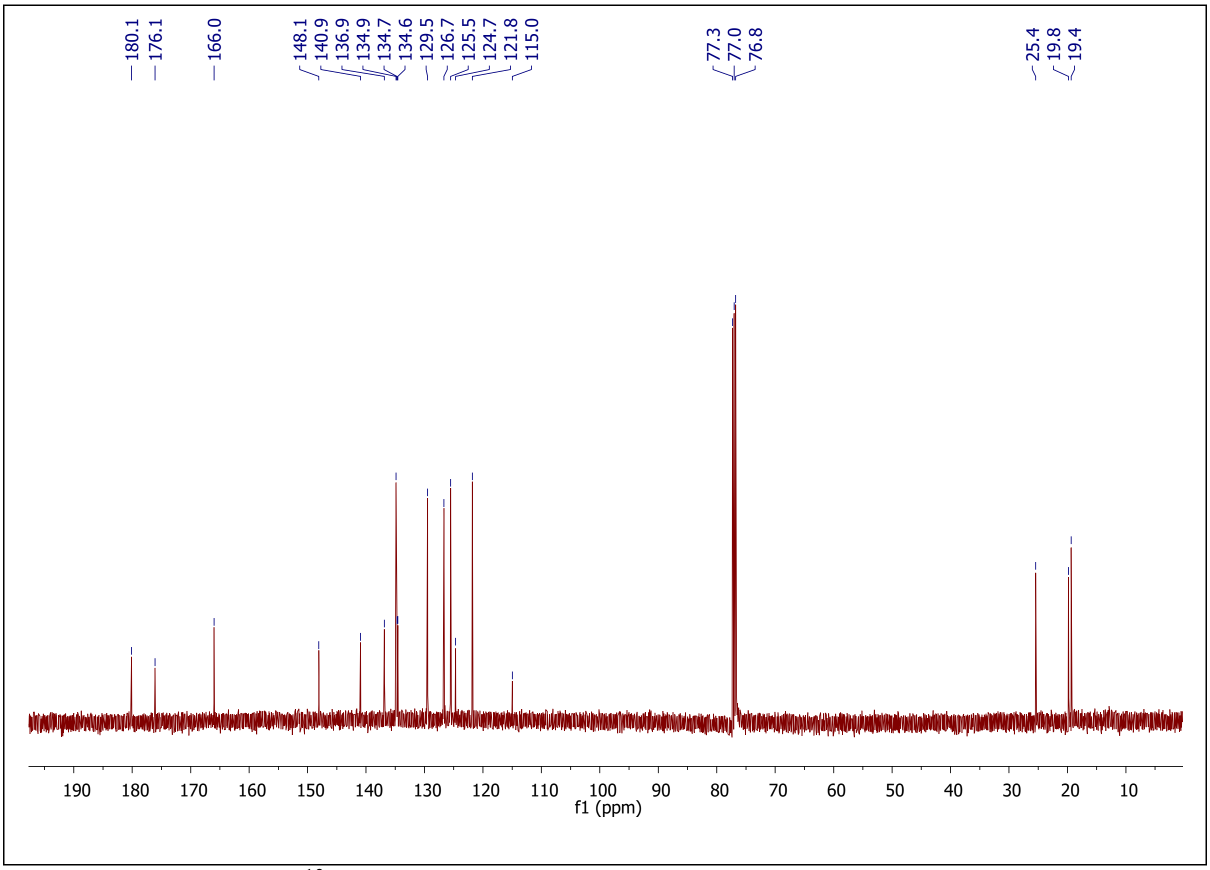

Figure S46. ^{13}C NMR (125 MHz) spectrum of the **AQQ15** in $CDCl_3$-*d*$_1$.

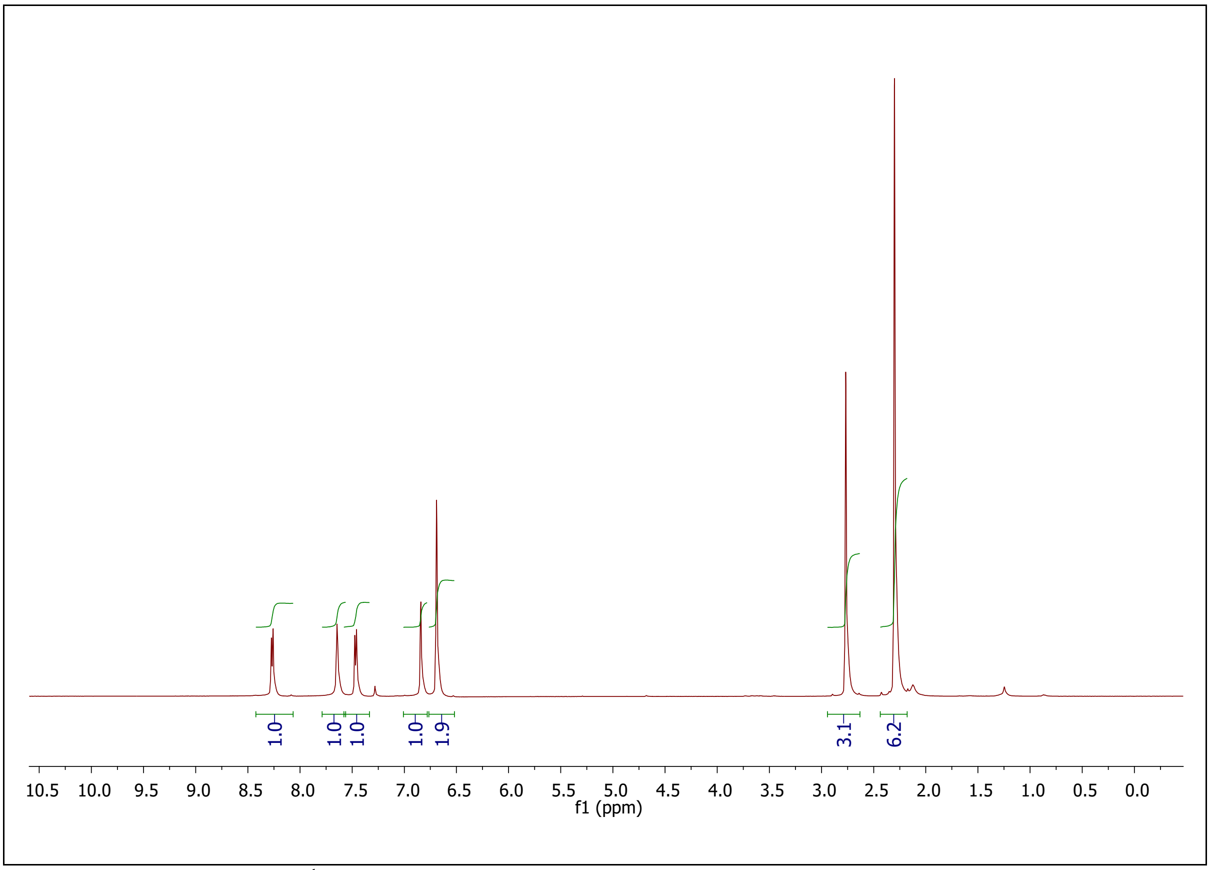

Figure S47. ^{1}H NMR (500 MHz) spectrum of the **AQQ16** in $CDCl_3$-d_1.

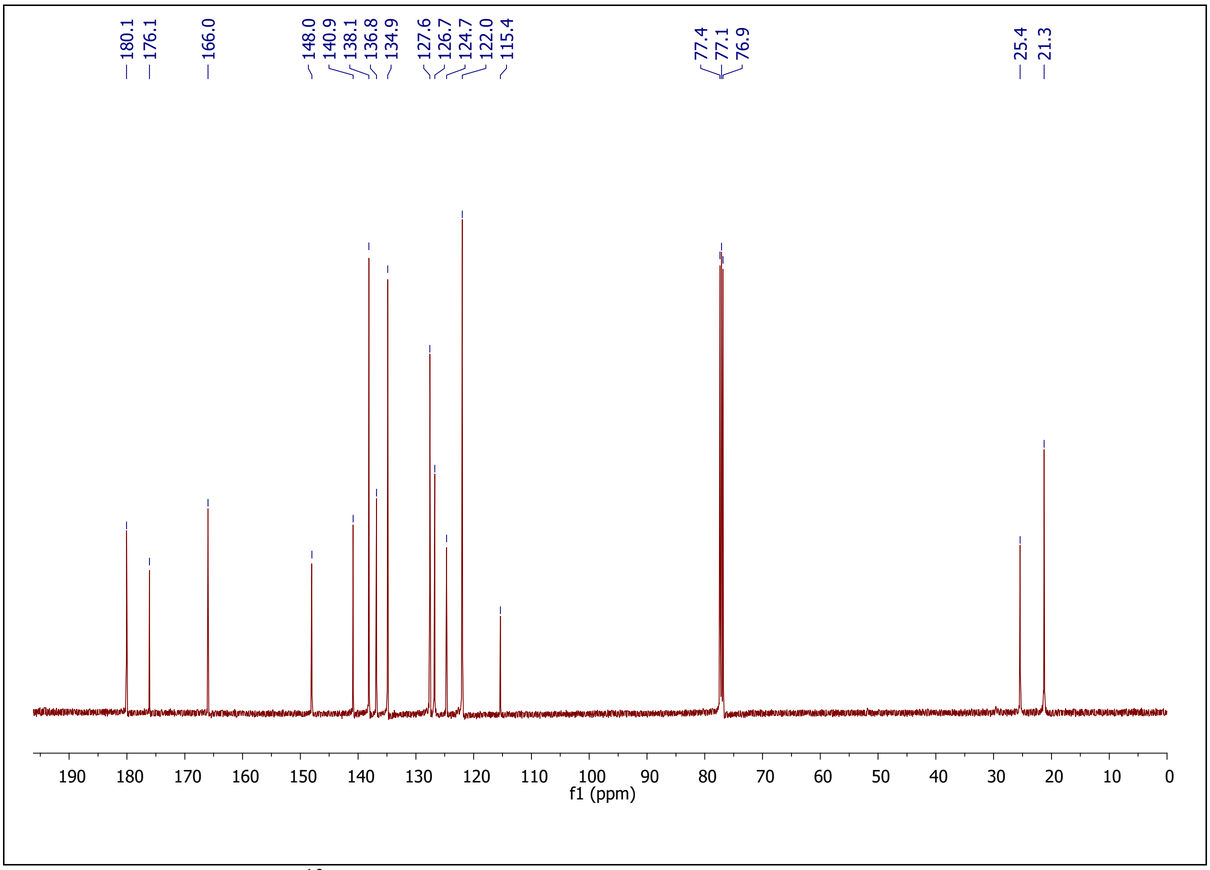

Figure S48. ^{13}C NMR (125 MHz) spectrum of the **AQQ16** in $CDCl_3$-d_1.
